# Green Ultrasound versus Conventional Synthesis and Characterization of Specific Task Pyridinium Ionic Liquid Hydrazones Tethering Fluorinated Counter Anions: Novel Inhibitors of Fungal Ergosterol Biosynthesis

**DOI:** 10.3390/molecules22111532

**Published:** 2017-11-07

**Authors:** Nadjet Rezki, Salsabeel A. Al-Sodies, Sheikh Shreaz, Rayees Ahmad Shiekh, Mouslim Messali, Vaseem Raja, Mohamed R. Aouad

**Affiliations:** 1 Department of Chemistry, Faculty of Sciences, Taibah University, P.O. Box 344, Al-Madinah Al-Munawarah 30002, Saudi Arabia; s7l_88@hotmail.com (S.A.A.-S.); rayeeschem@gmail.com (R.A.S.); aboutasnim@yahoo.fr (M.M.); 2 Laboratoire de Chimie et Electrochimie des Complexes Métalliques (LCECM) USTO-MB, Department of Chemistry, Faculty of Sciences, University of Sciences and Technology Mohamed Boudiaf, P.O. Box 1505, El M`nouar, Oran 31000, Algeria; 3 Environment and Life Sciences Research Center, Kuwait Institute for Scientific Research, P.O. Box 24885, Safat 13109, Kuwait; shreazresearch@gmail.com; 4 Government Degree College Pulwama, University of Kashmir, Srinagar 192301, India; 5 Department of Applied Sciences & Humanities, Faculty of Engineering & Technology, Jamia Millia Islamia, Central University, New Delhi 110025, India; vaseemchem99@gmail.com

**Keywords:** ionic liquids, hydrazones, fluorine, ultrasound, *Candida*, fungal ergosterol, confocal microscopy

## Abstract

A series of specific task ionic liquids (ILs) based on a pyridiniumhydrazone scaffold in combination with hexafluorophosphate (PF_6_^−^), tetrafluoroboron (BF_4_^−^) and/or trifluoroacetate (CF_3_COO^−^) counter anion, were designed and characterized by IR, NMR and mass spectrometry. The reactions were conducted under both conventional and green ultrasound procedures. The antifungal potential of the synthesized compounds **2**–**25** was investigated against 40 strains of *Candida* (four standard and 36 clinical isolates). Minimum inhibitory concentrations (MIC_90_) of the synthesized compounds were in the range of 62.5–2000 μg/mL for both standard and oral *Candida* isolates. MIC_90_ results showed that the synthesized 1-(2-(4-chlorophenyl)-2-oxoethyl)-4-(2-(4-fluorobenzylidene)hydrazinecarbonyl)-pyridin-1-ium hexafluorophosphate (**11**) was found to be most effective, followed by 4-(2-(4-fluorobenzylidene)hydrazinecarbonyl)-1-(2-(4-nitrophenyl)-2-oxoethyl)-pyridin-1-ium hexafluorophosphate (**14**) and 1-(2-ethoxy-2-oxoethyl)-4-(2-(4-fluorobenzylidene)hydrazinecarbonyl)pyridin-1-ium hexafluorophosphate (**8**). All the *Candida* isolates showed marked sensitivity towards the synthesized compounds. Ergosterol content was drastically reduced by more active synthesized compounds, and agreed well with MIC_90_ values. Confocal scanning laser microscopy (CLSM) results showed that the red colored fluorescent dye enters the test agent treated cells, which confirms cell wall and cell membrane damage. The microscopy results obtained suggested membrane-located targets for the action of these synthesized compounds. It appears that the test compounds might be interacting with ergosterol in the fungal cell membranes, decreasing the membrane ergosterol content and ultimately leading to membrane disruption as visible in confocal results. The present study indicates that these synthesized compounds show significant antifungal activity against *Candida* which forms the basis to carry out further in vivo experiments before their clinical use.

## 1. Introduction

*Candida* species are pleomorphic diploid organisms existing within the normal microflora of skin, oral cavity and gastrointestinal tract [1]. Pathogenic *Candida* species are responsible for mild cutaneous infections of the human body as well as life threatening systemic diseases [2]. As per the available reports, the last two decades have shown dramatic increase in the number of patients with deep fungal infections due to the increasing magnitude of acquired immune deficiency syndrome (AIDS) patients, patients with organ transplantations and increased use of intravenous lines or urinary catheters [3,4]. At present, infections due to *Candida* species are the leading cause of hematogenous infections worldwide [5]. It is well-known that most of the available antifungal drugs like azoles, allylamines and morpholines target ergosterol in the fungal cell membranes or inhibit the ergosterol biosynthetic pathway. Most of these antifungals, like amphotericin B and some commonly used azoles, are linked to severe toxicity issues [6,7], and in addition, most of the emerging fungal strains have developed resistance to the present antifungal agents [8,9]. 

During the last decades, the amazing properties associated with ionic liquids/salts that include low vapor pressure, high thermal stability, non volatility and inflammability have made them promising green solvents as alternatives to well-known organic solvents in organic chemistry [10,11,12,13,14,15]. They have also found applications in analytical chemistry, biotechnology and medicine [16,17,18]. Furthermore, the introduction of one or more functional groups was found to provide specific task ionic liquids (ILs) endowed with fascinating medical and industrial applications [19,20]. The synthesis of new fluorine-containing organic molecules has emerged as one of the hottest topics in medicinal research [21,22,23,24]. Therefore, fluorine-containing substituents were often incorporated to organic molecules during drug design to decrease toxicity [25], and increase lipophilicity, metabolitic stability and binding affinity [26]. In our recent work, some N-C2/C7 alkyl pyridinium hydrazones carrying fluorinated counteranions were synthesized and found to exhibit noteworthy antimicrobial activities against different pathogenic Gram-positive and Gram-negative bacteria [27], which stimulated us to investigate further research in this area. Inspired by these results, and in view of the promising bioactivity associated with the presence of fluorine substitution in organic molecules, we present here another significant synthetic contribution which aimed to preserve the basic structural skeleton of pyridinium hydrazone and fluorinated counter anions of lead compounds by changing the alkyl chain on the pyridine ring to a functionalized alkyl group. The antifungal activities of the synthesized compounds against different standard and various *Candida* species have been extensively investigated. 

Previous studies by our research group have shown that chemically synthesized molecules have shown promising antifungal activities against a diverse range of *Candida* species [28,29,30,31,32,33]. In this study, anticandidal activity of the synthesized compounds in liquid medium has been investigated through minimum inhibitory concentration (MIC_90_). Insights into the mechanism of antifungal action have been gained by examining the effect of these synthesized compounds on total ergosterol content and membrane integrity.

During the last years, ultrasonic irradiation has been extensively adopted in our laboratory as an eco-friendly energy source for the construction of several novel heterocyclic scaffolds [34,35,36]. Because of the promising features of ultrasound-assisted organic synthesis, namely the selectivity, ease of experimental set-up, enhanced reaction times and yields, as well as the environmental impact [37,38,39], a protocol in this current study has been directed to use this green technique for the synthesis of target ionic liquids tagged with Schiff bases in a comparative study with classical methods.

## 2. Results and Discussion

### 2.1. Chemistry

**Scheme 1:**
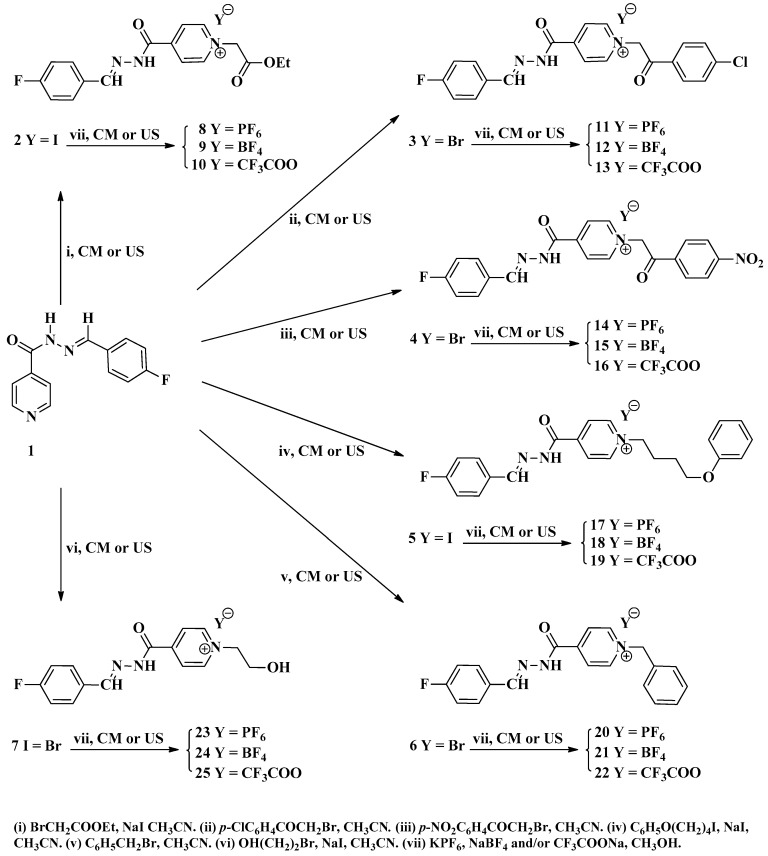
Conventional versus ultrasound synthesis of halogenated and fluorinated specific task ILs **2**–**25**.

The preparation and structure of fluorinated pyridinium hydrazones tailored functionalized alkyl side chains encountered with three fluorinated anions are illustrated in Scheme 1. Briefly, the precursor 4-fluorophenylpyridine hydrazone was treated by several functionalized alkyl halides to afford the halogenated specific task ionic liquid intermediates **2**–**7**, which then reacted under metathetical conditions with KPF_6_, NaBF_4_ and/or CF_3_COONa to give the target fluorinated specific task ILs **8**–**25**. The alkylation reaction required irradiation under ultrasound for 4–6 h to afford excellent yields (88–96%) of ILs **2**–**7**, which were alternatively synthesized in 80–93% yields after thermal heating for 20–48 h (Table 1). Bromide and/or iodide anion exchange has been achieved successfully by thermal treatment of the halogenated ionic liquids **2**–**7** for 16 h, and furnished the desired specific task ILs **8**–**25** in 83–94% yields. When these reactions were carried out under ultrasonic conditions, only 4–5 h were required and no changes in yields were noticed (Table 2).

The structure of the reaction products **2**–**7** was established by their spectral data (IR, ^1^H-NMR, ^13^C-NMR, ^19^F-NMR and mass spectroscopy in supporting information). In their IR spectra, the characteristic aliphatic hydrogens (CH_2_) appeared as a broad band around 2900 cm^−1^.

**Figure 1:**
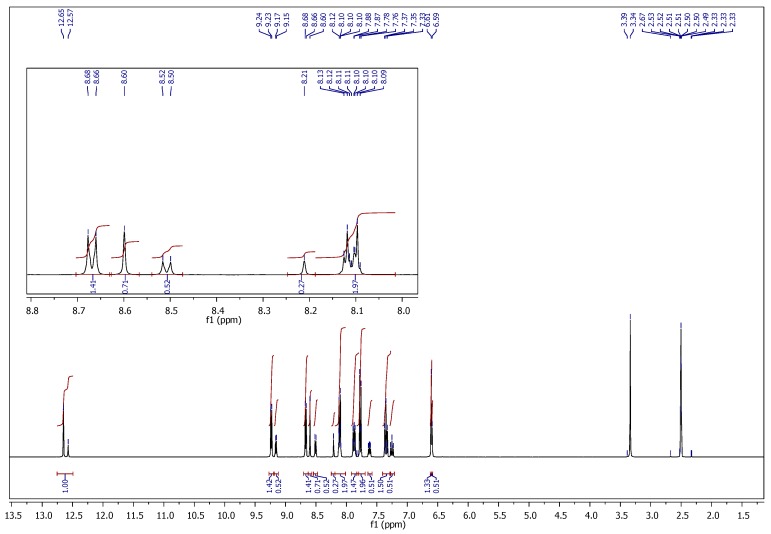
^1^H-NMR spectrum of compound **3**.

As an example, NMR of compound **3** will be described. The ^1^H-NMR spectrum displayed two characteristic singlets at δ_H_ 6.59 and 6.61 ppm with a ratio of 1:3, and integrated totally for two protons assigned to the NC**H**_2_ group, which confirmed the presence of such compound in their anti and syn conformers. As reported previously [40], the **H**-C=N and N**H** protons appeared as two sets of singlets at δ_H_ 8.21, 8.60 and 12.57, 12.65 ppm, respecively, presumaly due to E/cis and E/trans diastereomers (Figure 1). In addition, four extra aromatic protons were observed in the aromatic area confirming the presence of chlorophenyl ring in this compound.

**Figure 2:**
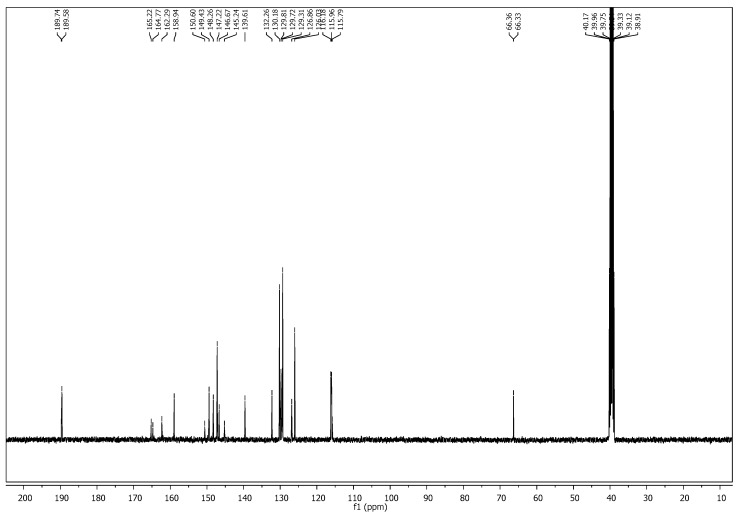
^13^C-NMR spectrum of compound **3**.

The presence of compound **3** as a mixture of E/cis and E/trans diastereomers was further supported by the ^13^C-NMR experiment, which revealed the appearance of each signal as double peaks. Thus, the methylene carbon split into two distinguished signals at δ_C_ 66.33 and 66.36 ppm, presumably due to the hydrazone stereoisomerism. In the downfield region, the hydrazone C=N and C=O groups of E/cis and E/trans diastereomers resonated as two sets of signals at δ_C_ 158.94, 162.29 and 164.77, 165.22 ppm, respectively. In addition, the aromatic carbons were also recorded in their appropriate region and the two signals at 189.58, 189.74 ppm were attributed to the ketone carbonyl (C=O) group (Figure 2).

**Figure 3:**
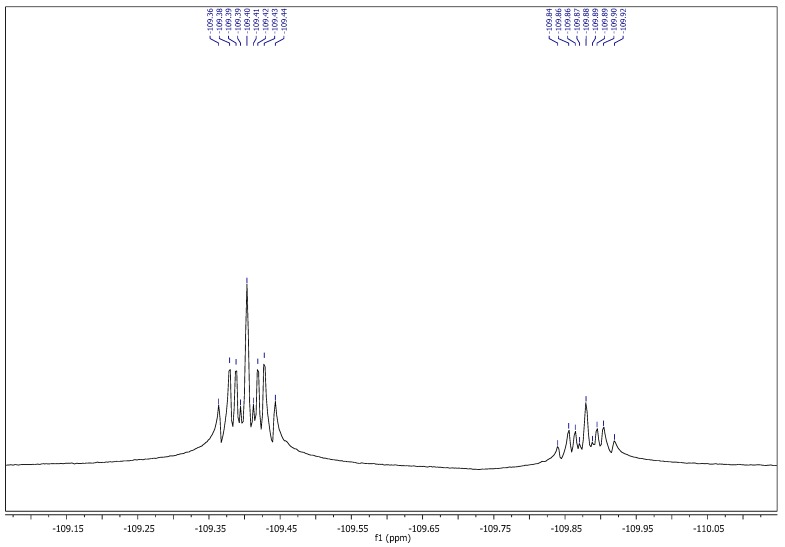
^19^F-NMR spectrum of compound **3**.

The ^19^F-NMR spectrum was also used in the establishment of such structure. The spectrum exhibited two signals which resonated as two multiplets at δ_F_ (−109.92 to −109.84) ppm and (−109.44 to −109.36) ppm assigning to aromatic fluorine (Figure 3).

The structures of the fluorinated specific task ILs **8**–**25** have been established on the basis of their spectral data using different NMR experiments, including ^1^H-NMR, ^13^C-NMR, ^19^F-NMR, ^31^P-NMR and ^11^B-NMR. No change in the protons and carbons assignment has been observed in the ^1^H- and ^13^C-NMR spectra of compounds **8**–**25** after the metathesis reactions, while their ^19^F-NMR, ^31^P-NMR and ^11^B-NMR facilitated the anion assignment.

**Figure 4:**
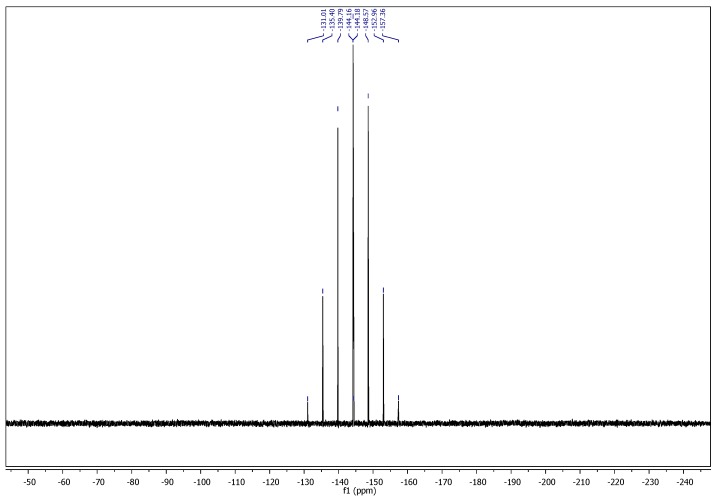
^31^P-NMR spectrum of compound **11.**

**Figure 5:**
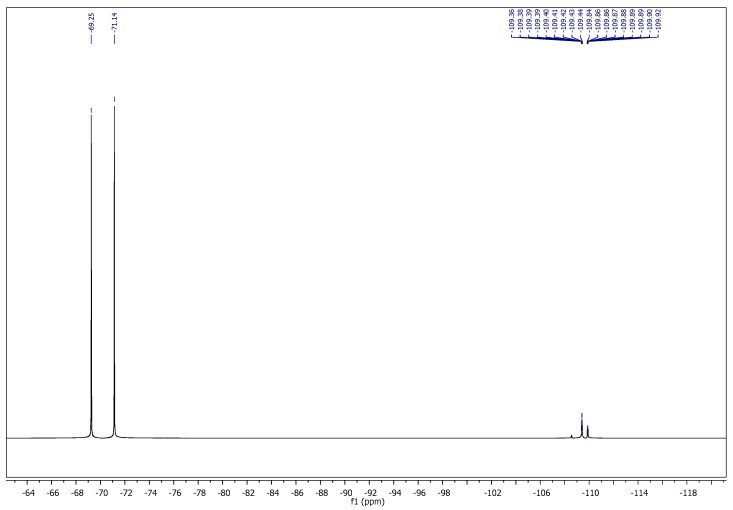
^19^F-NMR spectrum of compound **11**.

The structure of **11** was deduced from its ^31^P-NMR spectrum which clearly showed the presence of a phosphorus atom that resonated as a multiplet at δ_P_ −157.36 to −131.01 ppm (Figure 4). The signals corresponding to fluorine atoms were characterized by their ^19^F-NMR spectrum as two singlets at δ_F_ −71.14 and −69.25 ppm confirming the presence of PF_6_^−^ anion in the anion head group of compound **11**. The spectrum also revealed the presence of aromatic fluorine (Figure 5).

The presence of BF_4_^−^ anion in compound **12** as an anion head has been confirmed by ^11^B- and ^19^F-NMR spectra. The boron atom resonated as a doublet at δ_B_ −1.28 ppm, and the fluorine atom was characterized as two doublets at δ_F_ −148.36 and −148.30 ppm, respectively. The fluorine phenyl atom was observed as two multiplets at δ_F_ (−109.94 to −109.77) and (−109.47 to −109.35) ppm. 

Another metathesis reaction has been achieved through the displacement of the bromide anion by trifluoroacetate and resulted in the formation of IL **13**. Its structure has been established based on the ^19^F-NMR by the appearance of the characteristic singlet peak at δ_F_ −73.50 ppm. The two muliplet signals related to the aromatic fluorine appeared at δ_F_ (−109.98 to −109.90) and (−109.52 to −109.44) ppm.

### 2.2. Antifungal Screening

#### 2.2.1. Determination of MIC_90_

This is a standard and valid parameter to check the antifungal susceptibility of chemically synthesized molecules. Table 3 shows the *Candida* isolates used in this study. Table 4, shows the range of minimum inhibitory concentration of the synthesized compounds in μg/mL against 40 strains of *Candida* (four standard and 36 clinical isolates). MIC_90_ results showed that synthesized compound **11** was found to be more effective, followed by compound **14** and compound **8**. As per the results, varying degrees of antifungal activities were shown by the synthesized compounds. Generally, all the *Candida* isolates investigated were found to be sensitive to these synthesized compounds. The MIC results reflected the antifungal potential of each synthesized compound. A closer look at the structures of the most active compounds (**8**, **11** and **14**) indicates that the presence of the PF_6_^−^ counter ion has significantly enhanced the activity of these compounds. It may be suggested that the PF_6_^−^ ion in these compounds might have created a favorable environment for these compounds to interact with the target, ergosterol. PF_6_^−^ is more effective (owing to the presence of six electronegative fluorine atoms around a phosphorus atom) to interact with the positive portions/sites (possibilities of non-covalent interactions such as formation of hydrogen bonds and salt bridges) of the cell membrane; thus enabling the positively charged **8**, **11** and **14** to escape the unnecessary repulsions/hurdles with the cell membranes. The ineffectiveness of BF_4_^−^ and CH_3_COO^−^ might be attributed to the electron-deficient nature of boron, and the delocalization of the negative charge on the carboxylic group of the acetate anion. This may have helped these three compounds to reach ergosterol with ease and cause the death of the fungal cells. The higher activity of **11** and **14,** as compared to **8,** is probably due to the insertion of the terminal substituted aromatic moieties in **11** and **14**, which might have enabled these two compounds to interact better with ergosterol via π-π interactions/stacking. However, the decreased activity of **11** in comparison to **14** may be explained on the basis of the greater steric influence of the bulky NO_2_ group as compared to the relatively smaller chlorine atom.

#### 2.2.2. Effect of the Synthesized Compounds on Membrane Ergosterol Content

**Figure 6:**
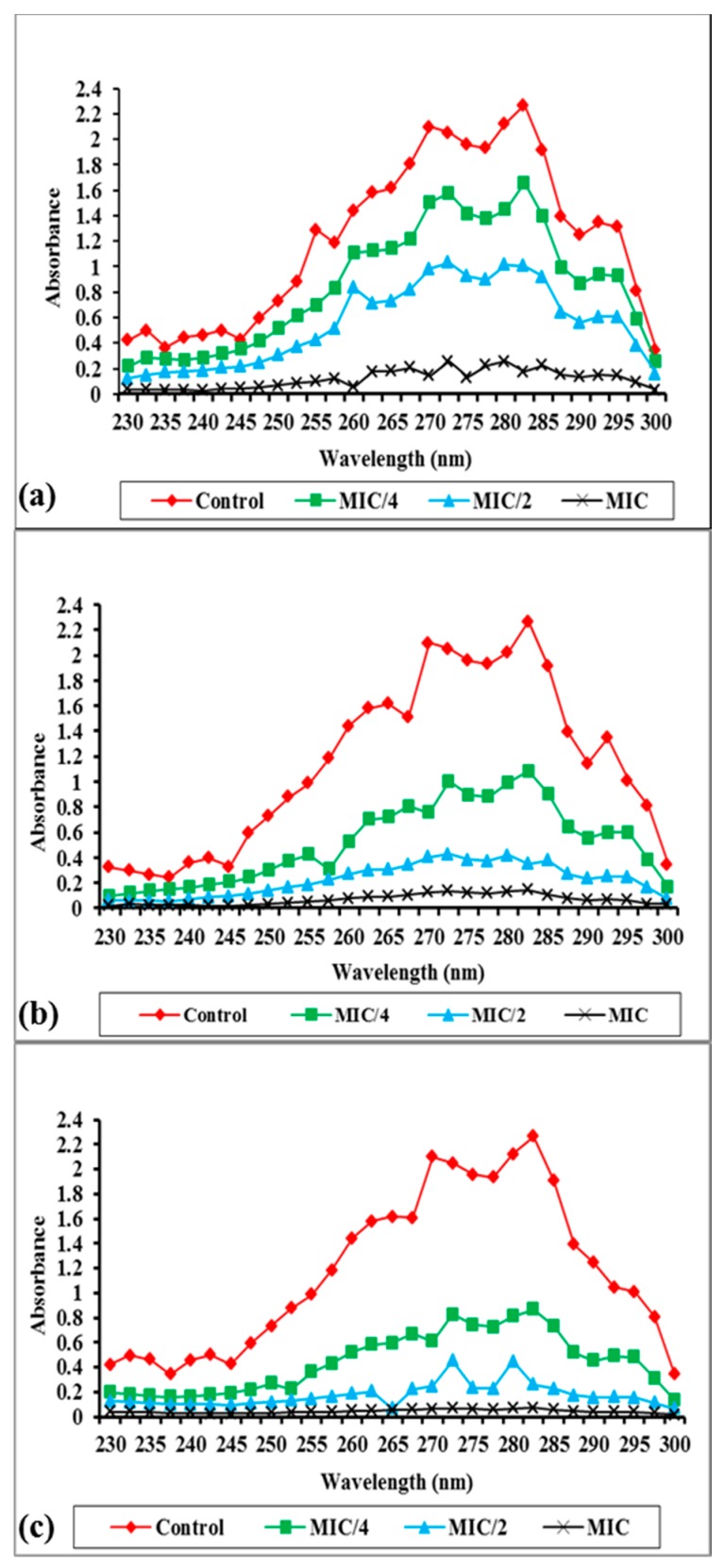
UV spectrophotometric sterol profile of (**a**) *C. albicans* ATCC 10261 (**b**) *C. albicans* CN 9 (**c**) *C. albicans* CN 38 grown for 16 h in YPD broth containing MIC/4, MIC/2 and MIC of compound **11**. UV- Spectral profiles of extracted sterols were read between 230–300 nm. Control implies without any treatment. In the cases of MIC/4 and MIC/2, cells were treated with one fourth and one half of MIC (μg/mL) value of test compound, respectively. In the MIC case, the sample was treated with MIC (μg/mL) value of test compound. The MIC_90_ for compound 11 against *C. albicans* ATCC 10261, *C. albicans* CN 9, and *C. albicans* CN 38 was 62.5, 31.25 and 62.5 μg/mL, respectively.

Figure 6a–c shows the effect of the highest active synthesized compound on the UV spectrophotometric sterol profiles of different *C. albicans* isolates (*C. albicans* ATCC 10261, *C. albicans* CN 9 and *C. albicans* CN 38) when grown for 16 h. In the graphs, control cells with normal ergosterol content show a characteristic four-peaked curve (red color). In case of cells treated with compound **11**, it was observed that as the concentration of the synthesized compound increases from MIC/4, MIC/2 to MIC, suppression of four peaks in the curve is observed which is denoted by green, blue and black curves. The results obtained show a visible effect of test compound concentrations on the sterol profile of three different *Candida* strains. In our previous study, the total ergosterol content decreased in the same fashion in the presence of MIC and sub-MIC values of the synthesized compounds [28]. In this study, the average percent decrease in total ergosterol content for three standard isolates and three clinical *Candida* isolates after exposure to MIC and sub-MIC values of compounds **8**, **14** and **11** have been summarized in Table 5. From the results, it is clear that as the concentration of the synthesized compounds increases, ergosterol content decreases and finally at MIC value, almost a straight line is seen that indicates the absence ergosterol in the sample.

The plasma membranes of fungi are almost similar to the plasma membranes of the mammals; the only difference is that fungi have non-polar sterol ergosterol and mammals have cholesterol as the principal sterol [41]. The plasma membrane being selectively permeable is responsible for transport of materials in and out of the cell. In addition, the control of some physiological events, modulation of membrane fluidity and membrane structure are provided and maintained by membrane sterols [42]. 

Yeast membrane ergosterol is the most common antifungal target studied so far, available reports show that ergosterol or its biosynthetic pathway are the sites of interaction for most antifungal agents [43]. In this study, an attempt was made to explore the possible target sites for the antifungal actions of the active synthesized compounds. It was observed that the synthesized compounds exposure resulted in dose dependent decrease in ergosterol content (Figure 6a–c). No systematic difference in ergosterol content was seen between standard and clinical *Candida* isolates used in this study.

#### 2.2.3. Confocal Scanning Laser Microscopy (CSLM)

**Figure 7:**
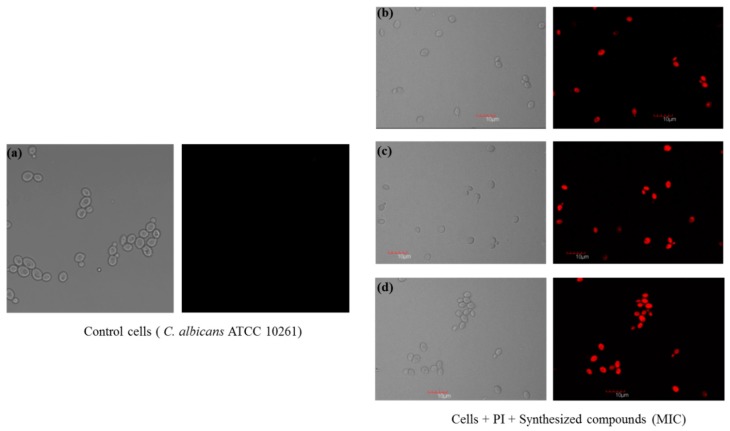
Laser confocal images of *C. albicans* ATCC 10261. To confirm membrane damage, cells were stained with propidium iodide (PI; red signals). (**a**) Control (**b**) Cells treated with compound **8** (**c**) Cells treated with compound **14** (**d**) Cells treated with compound **11**. Left panel contains *Candida* cells without any treatment; in this case only dye was added to the cells. No red color was seen in the confocal image in the left panel (control cells); this implies that the dye has not entered the cells. In the right panel (treated cells), the red color is quite visible, implying that cell membranes and cell walls have been disrupted.

This experiment was done to localize and confirm cell membrane damage in the presence of the highest active test compounds (**8**, **11** and **14**). Cells were incubated with and without the synthesized compounds in the presence of propidium iodide (PI). PI is a red dye employed to evaluate the effect of drugs on cell membranes. Compound treatment was given initially, and then the dye was added to cells after 16 h of incubation with the test compounds. The dye slowly gets dissolved in the liquid media, and when a drug causes breakage of the cell, the dye present within the media enters inside the broken cells. Cells with severe damage of membrane and cell wall will internalize PI which forms a red color inside resulting in an increased red fluorescence. The laser confocal images of stained *C. albicans* ATCC 10261 cells exposed to MIC values of compounds **8**, **14** and **11** are shown in Figure 7b–d, respectively. The panel on the left side shows the confocal microscopy results of stained *Candida* cells without any treatment (Figure 7a), while the panel on right side shows confocal results of cells exposed to PI and MIC values of compounds **8**, **14** and **11**. The results obtained confirm that the dye penetrates the cells treated with MIC values of the test compounds, implying that the cell membrane and cell wall were disrupted by the synthesized compounds. The results showed that in case of compound **11** treatments, most of the yeast cells appeared red which indicates that the antifungal effect is much superior when cells are exposed to the MIC value of compound **11**. In the present study, an attempt was made to assess the antifungal role of these synthesized compounds by studying their effect on membrane integrity. Permeation to PI, following incubation with MIC values of the synthesized compounds, indicates that these compounds directly target the cell membrane and form severe lesions on the membrane that ultimately leads to cell death.

## 3. Experimental Section

### 3.1. Chemistry

Melting points were recorded on a Stuart Scientific SMP1 apparatus and are uncorrected. The IR spectra were measured using an FTIR-8400s-Fourier transform infrared spectrophotometer-Shimadzu. The NMR spectra were determined on Advance Bruker (Fällanden, Switzerland) NMR spectrometer at 400 and 600 MHz with TMS as internal standard. The ESI mass spectra were measured by a Finnigan LCQ spectrometer. Reactions performed under ultrasounds were carried out using a Kunshan KQ-250B ultrasound cleaner (50 KHz, 240 W). All solvents and reagents were purchased from Sigma-Aldrich (Hamburg, Germany).

### 3.2. General Alkylation Procedure for the Synthesis of Halogenated Specific Task ILs Tagged Hydrazones ***2***–***7***

#### 3.2.1. Conventional Method (CM)

A mixture of compound **1** (1 mmol) in acetonitrile (30 mL) and functionalized alkyl halides (1.3 mmol) was refluxed for 20–48 h. The solvent was reduced by evaporation under reduced pressure; the precipitate formed was collected by filtration and washed with acetonitrile to afford the desired ionic liquids **2**–**7**.

#### 3.2.2. Ultrasound Method (US)

A mixture of compound **1** (1 mmol) in acetonitrile (30 mL) and functionalized alkyl halides (1.3 mmol) was irradiated by ultrasound irradiation. The reaction was processed as described above to give the same ionic liquids **2**–**7**.

*1-(2-Ethoxy-2-oxoethyl)-4-(2-(4-fluorobenzylidene)hydrazinecarbonyl)pyridin-1-ium iodide* (**2**). It was obtained as brown crystals; m.p.: 113–114 °C. FT-IR (KBr): ῡ =1310 (C-O), 1599 (C=N), 1688 (C=O), 2890 and 2930 (Al-H), 3065 cm^−1^ (Ar-H). ^1^H-NMR (400 MHz, DMSO-*d*_6_): δ_H_ = 1.28 (t, 3H, *J* = 8 Hz, CH_2_C**H**_3_), 4.25–4.31 (m, 2H, C**H**_2_CH_3_), 5.73 (s, 0.5H, NC**H**_2_), 5.74 (s, 1.5H, NC**H**_2_), 7.26 (dd, 0.5H, *J* = 8 Hz, 12 Hz, Ar-**H**), 7.35 (t, 1.5H, *J* = 8 Hz, Ar-**H**), 7.61 (dd, 0.5H, *J* = 4 Hz, 8 Hz, Ar-**H**), 7.90 (dd, 1.5H, *J* = 4 Hz, 8 Hz, Ar-**H**), 8.17 (s, 0.25H, **H**-C=N), 8.48 (d, 0.5H, *J* = 8 Hz, Ar-**H**), 8.49 (s, 0.75H, **H**-C=N), 8.60 (d, 1.5H, *J* = 8 Hz, Ar-**H**), 9.19 (d, 0.5H, *J* = 4 Hz, Ar-**H**), 9.26 (d, 1.5H, *J* = 4 Hz, Ar-**H**), 12.52 (s, 0.75H, CON**H**), 12.55 (s, 0.25H, CON**H**). ^13^C-NMR (100 MHz, DMSO-*d*_6_): δ_C_ =13.93 (CH_2_**C**H_3_), 60.49, 60.53 (**C**H_2_CH_3_), 62.42, 62.47 (N**C**H_2_), 115.77, 115.97, 116.19, 125.99, 126.83, 129.32, 129.41, 129.75, 129.84, 129.99, 130.18, 130.21, 145.26, 146.66, 147.24, 149.44, 150.90 (Ar-**C**), 158.86, 162.30, 164.78, 165.16 (**C**=N, **C**=O), 166.12, 166.19 (**C**=OO). ^19^F-NMR (377 MHz, DMSO-*d*_6_): δ_F_ = (−109.82 to −109.74), (−109.38 to −109.30) (2m, 1F, Ar-**F**). MS (ESI) *m*/*z* = 457.29 [M^+^].

*1-(2-(4-Chlorophenyl)-2-oxoethyl)-4-(2-(4-fluorobenzylidene)hydrazinecarbonyl)-pyridin-1-ium bromide* (**3**). It was obtained as yellow crystals; m.p.: 122–123 °C. FT-IR (KBr): ῡ = 1630 (C=N), 1655 (C=O), 2925 (Al-H), 3066 cm^−1^ (Ar-H). ^1^H-NMR (400 MHz, DMSO-*d*_6_): δ_H_ = 6.59 (s, 0.5H, NC**H**_2_), 6.61 (s, 1.5H, NC**H**_2_), 7.25 (t, 0.5H, *J* = 8 Hz, Ar-**H**), 7.35 (t, 1.5H, *J* = 8 Hz, Ar-**H**), 7.64 (dd, 0.5H, *J* = 4 Hz, 8 Hz, Ar-**H**), 7.78 (d, 2H, *J* = 8 Hz, Ar-**H**), 7.89 (dd, 1.5H, *J* = 4 Hz, 8 Hz, Ar-**H**), 8.12 (d, 2H, *J* = 8 Hz, Ar-**H**), 8.21 (s, 0.25H, **H**-C=N), 8.52 (d, 0.5H, *J* = 8 Hz, Ar-**H**), 8.60 (s, 0.75H, **H**-C=N), 8.68 (d, 1.5H, *J* = 8 Hz, Ar-**H**), 9.17 (d, 0.5H, *J* = 8 Hz, Ar-**H**), 9.24 (d, 1.5H, *J* = 4 Hz, Ar-**H**), 12.57 (s, 0.25H, CON**H**), 12.65 (s, 0.75H, CON**H**). ^13^C-NMR (100 MHz, DMSO-*d*_6_): δ_C_ = 66.33, 66.36 (N**C**H_2_), 115.79, 115.96, 116.18, 126.03, 126.86, 129.31, 129.44, 129.72, 129.81, 130.18, 130.24, 132.26, 132.31, 139.58, 139.61, 145.24, 146.67, 147.22, 148.26, 149.43, 150.60 (Ar-**C**), 158.94, 162.29, 164.77, 165.22 (**C**=N, **C**=O), 189.58, 189.74 (CH_2_**C**=O). ^19^F-NMR (377 MHz, DMSO-*d*_6_): δ_F_ = (−109.92 to −109.84), (−109.44 to −109.36) (2m, 1F, Ar-**F**). MS (ESI) *m*/*z* = 475.01 [M^+^].

*4-(2-(4-Fluorobenzylidene)hydrazinecarbonyl)-1-(2-(4-nitrophenyl)-2-oxoethyl)-pyridin-1-ium bromide* (**4**). It was obtained as orange crystals; m.p.: 109–110 °C. FT-IR (KBr): ῡ =1380 and 1550 (NO_2_), 1612 (C=N), 1640 (C=O), 2922 (Al-H), 3070 cm^−1^ (Ar-H). ^1^H-NMR (400 MHz, DMSO-*d*_6_): δ_H_ = 6.63 (s, 0.5H, NC**H**_2_), 6.65 (s, 1.5H, NC**H**_2_), 7.28 (dd, 0.5H, *J* = 8 Hz, 12 Hz, Ar-**H**), 7.38 (dd, 1.5H, *J* = 8 Hz, 12 Hz, Ar-**H**), 7.64 (dd, 0.5H, *J* = 4 Hz, 8 Hz, Ar-**H**), 7.90 (dd, 1.5H, *J* = 4 Hz, 8 Hz, Ar-**H**), 8.21 (s, 0.25H, **H**-C=N), 8.31–8.34 (m, 2H, Ar-**H**), 8.48–8.51 (m, 2H, Ar-**H**), 8.53 (d, 0.5H, *J* = 4 Hz, Ar-**H**), 8.57 (s, 0.75H, **H**-C=N), 8.68 (d, 1.5H, *J* = 8 Hz, Ar-**H**), 9.16 (d, 0.5H, *J* = 8 Hz, Ar-**H**), 9.23 (d, 1.5H, *J* = 4 Hz, Ar-**H**), 12.57 (s, 0.25H, CON**H**), 12.63 (s, 0.75H, CON**H**). ^13^C-NMR (100 MHz, DMSO-*d*_6_): δ_C_ = 66.61, 66.66 (N**C**H_2_), 115.79, 115.97, 116.19, 124.19, 126.09, 126.93, 129.35, 129.44, 129.75, 129.82, 130.23, 138.17, 138.22, 145.27, 146.69, 147.25, 148.43, 149.44, 150.69 (Ar-**C**), 158.96, 162.31, 164.78, 165.21 (**C**=N, **C**=O), 189.80, 189.95 (CH_2_**C**=O). ^19^F-NMR (377 MHz, DMSO-*d*_6_): δ_F_ = (−109.93 to −109.85), (−109.46 to −109.38) (2m, 1F, Ar-**F**). MS (ESI) *m*/*z* = 486.90 [M^+^].

*4-(2-(4-Fluorobenzylidene)hydrazinecarbonyl)-1-(4-phenoxybutyl)pyridin-1-ium iodide* (**5**). It was obtained as yellow crystals; m.p.: 105–106 °C. FT-IR (KBr): ῡ =1315 (C-O), 1616 (C=N), 1690 (C=O), 2935 (Al-H), 3070 cm^−1^ (Ar-H). ^1^H-NMR (400 MHz, DMSO-*d*_6_): δ_H_ = 1.75–1.83 (m, 2H, C**H**_2_CH_2_N), 2.11–2.21 (m, 2H, C**H**_2_CH_2_O), 4.01–4.05 (m, 2H, C**H**_2_N), 4.78 (t, 2H, *J* = 8 Hz, OC**H**_2_), 6.91–6.95 (m, 3H, Ar-**H**), 7.24 (dd, 0.5H, *J* = 8 Hz, 12 Hz, Ar-**H**), 7.29 (t, 2H, *J* = 8 Hz, Ar-**H**), 7.34 (t, 1.5H, *J* = 8 Hz, Ar-**H**), 7.62 (dd, 0.5H, *J* = 4 Hz, 8 Hz, Ar-**H**), 7.89 (dd, 1.5H, *J* = 4 Hz, 8 Hz, Ar-**H**), 8.16 (s, 0.25H, **H**-C=N), 8.41 (d, 0.5H, *J* = 8 Hz, Ar-**H**), 8.52 (s, 0.75H, **H**-C=N), 8.55 (d, 1.5H, *J* = 8 Hz, Ar-**H**), 9.28 (d, 0.5H, *J* = 8 Hz, Ar-**H**), 9.37 (d, 1.5H, *J* = 8 Hz, Ar-**H**), 12.46 (bs, 1H, CON**H**). ^13^C-NMR (100 MHz, DMSO-*d*_6_): δ_C_ = 24.55, 24.60 (**C**H_2_CH_2_N), 26.87, 26.96 (**C**H_2_CH_2_O), 59.98, 60.05 (**C**H_2_N), 65.88, 65.92 (O**C**H_2_), 113.72, 115.03, 115.23, 115.45, 119.84, 125.47, 126.46, 128.72, 129.01, 129.09, 129.53, 144.40, 145.02, 146.66, 148.69, 148.96 (Ar-**C**), 157.67, 158.02, 161.57, 164.04, 164.45 (**C**=N, **C**=O). ^19^F-NMR (377 MHz, DMSO-*d*_6_): δ_F_ = (−109.92 to −109.84), (−109.43 to −109.35) (2m, 1F, Ar-**F**). MS (ESI) *m*/*z* = 519.00 [M^+^].

*1-Benzyl-4-(2-(4-fluorobenzylidene)hydrazinecarbonyl)pyridin-1-ium bromide* (**6**). It was obtained as yellow crystals; m.p.: 94–95 °C. FT-IR (KBr): ῡ = 1590 (C=N), 1655 (C=O), 2935 (Al-H), 3080 cm^−1^ (Ar-H). ^1^H-NMR (400 MHz, DMSO-*d*_6_): δ_H_ = 6.00 (s, 2H, NC**H**_2_), 7.22 (t, 0.5H, *J* = 8 Hz, Ar-**H**), 7.33 (t, 1.5H, *J* = 8 Hz, Ar-**H**), 7.44–7.49 (m, 3H, Ar-**H**), 7.59 (dd, 0.5H, *J* = 4 Hz, 8 Hz, Ar-**H**), 7.60–7.62 (m, 2H, Ar-**H**), 7.86 (dd, 1.5H, *J* = 4 Hz, 8 Hz, Ar-**H**), 8.18 (s, 0.25H, **H**-C=N), 8.42 (d, 0.5H, *J* = 4 Hz, Ar-**H**), 8.59 (s, 0.75H, **H**-C=N), 8.60 (d, 1.5H, *J* = 4 Hz, Ar-**H**), 9.39 (d, 0.5H, *J* = 4 Hz, Ar-**H**), 9.51 (d, 1.5H, *J* = 8 Hz, Ar-**H**), 12.52 (s, 0.25H, CON**H**), 12.59 (s, 0.75H, CON**H**). ^13^C-NMR (100 MHz, DMSO-*d*_6_): δ_C_ = 63.34, 63.39 (N**C**H_2_), 115.75, 115.94, 116.16, 126.63, 127.51, 128.93, 128.97, 129.23, 129.34, 129.46, 129.69, 129.78, 130.22, 130.25, 134.02, 134.06, 145.18, 145.74, 147.70, 149.39 (Ar-**C**), 158.71, 162.26, 164.74, 165.99 (**C**=N, **C**=O). ^19^F-NMR (377 MHz, DMSO-*d*_6_): δ_F_ = (−109.83 to −109.75), (−109.41 to −109.33) (2m, 1F, Ar-**F**). MS (ESI) *m*/*z* = 413.90 [M^+^].

*4-(2-(4-Fluorobenzylidene)hydrazinecarbonyl)-1-(2-hydroxyethyl)pyridin-1-ium iodide* (**7**). It was obtained as brown crystals; m.p.: 97–98 °C. FT-IR (KBr): ῡ = 1320 (C-O), 1590 (C=N), 1655 (C=O), 2924 (Al-H), 3079 cm^−1^ (Ar-H). ^1^H-NMR (400 MHz, DMSO-*d*_6_): δ_H_ = 3.89–3.93 (m, 2H, OC**H**_2_), 4.74 (t, 2H, *J* = 4 Hz, NC**H**_2_), 7.26 (dd, 0.5H, *J* = 8 Hz, 12 Hz, Ar-**H**), 7.37 (dd, 1.5H, *J* = 8 Hz, 12 Hz, Ar-**H**), 7.62 (dd, 0.5H, *J* = 4 Hz, 8 Hz, Ar-**H**), 7.89 (dd, 1.5H, *J* = 4 Hz, 8 Hz, Ar-**H**), 8.16 (s, 0.25H, **H**-C=N), 8.40 (d, 0.5H, *J* = 4 Hz, Ar-**H**), 8.49 (s, 0.75H, **H**-C=N), 8.53 (d, 1.5H, *J* = 8 Hz, Ar-**H**), 9.15 (d, 0.5H, *J* = 8 Hz, Ar-**H**), 9.23 (d, 1.5H, *J* = 8 Hz, Ar-**H**), 12.48 (s, 0.75H, CON**H**), 12.50 (s, 0.25H, CON**H**). ^13^C-NMR (100 MHz, DMSO-*d*_6_): δ_C_ = 60.00 (O**C**H_2_), 63.37, 63.44 (N**C**H_2_), 115.77, 115.97, 116.19, 125.80, 126.83, 129.37, 129.46, 129.73, 129.82, 130.01, 130.04, 130.20, 130.23, 145.17, 145.48, 146.11, 147.57, 148.38, 149.71 (Ar-**C**), 158.90, 162.29, 164.76, 165.23 (**C**=N, **C**=O). ^19^F-NMR (377 MHz, DMSO-*d*_6_): δ_F_ = (−109.87 to −109.76), (−109.40 to −109.35) (2m, 1F, Ar-**F**). MS (ESI) *m*/*z* = 415.38 [M^+^].

### 3.3. General Metathesis Procedure for the Synthesis of Fluorinated Specific Task ILs ***8***–***25***

#### 3.3.1. Conventional Method (CM)

A mixture of ionic liquid **2**–**7** (1 mmol) in methanol (30 mL) and potassium hexafluorophosphate, sodium tetrafluoroborate, and/or sodium trifluoroacetate (1.2 mmol) was refluxed for 16 h. The precipitate formed was collected by filtration and washed with the appropriate solvent to give the expected ionic liquids **8**–**25**.

#### 3.3.2. Ultrasound Method (US)

A mixture of ionic liquid **2**–**7** (1 mmol) in dichloromethane, acetonitrile or methanol (30 mL), and potassium hexafluorophosphate, sodiumtetrafluoroborate, and/or sodium trifluoroacetate (1.2 mmol) was irradiated by ultrasound irradiation. The reaction was processed as described above to give the same ionic liquids **8**–**25**.

*1-(2-Ethoxy-2-oxoethyl)-4-(2-(4-fluorobenzylidene)hydrazinecarbonyl)pyridin-1-ium hexafluorophosphate* (**8**). It was obtained as brown crystals; m.p.: 81–82 °C. ^1^H-NMR (400 MHz, DMSO-*d*_6_): δ_H_ = 1.28 (t, 3H, *J* = 8 Hz, CH_2_C**H**_3_), 4.24–4.30 (m, 2H, C**H**_2_CH_3_), 5.73 (s, 0.5H, NC**H**_2_), 5.74 (s, 1.5H, NC**H**_2_), 7.25 (t, 0.5H, *J* = 8 Hz, Ar-**H**), 7.37 (dd, 1.5H, *J* = 8 Hz, 12 Hz, Ar-**H**), 7.61 (dd, 0.5H, *J* = 4 Hz, 8 Hz, Ar-**H**), 7.89 (dd, 1.5H, *J* = 4 Hz, 8 Hz, Ar-**H**), 8.17 (s, 0.25H, **H**-C=N), 8.48 (d, 0.5H, *J* = 8 Hz, Ar-**H**), 8.49 (s, 0.75H, **H**-C=N), 8.60 (d, 1.5H, *J* = 8 Hz, Ar-**H**), 9.19 (d, 0.5H, *J* = 8 Hz, Ar-**H**), 9.26 (d, 1.5H, *J* = 8 Hz, Ar-**H**), 12.51 (s, 0.75H, CON**H**), 12.54 (s, 0.25H, CON**H**). ^13^C-NMR (100 MHz, DMSO-*d*_6_): δ_C_ =13.92 (CH_2_**C**H_3_), 60.49, 60.53 (**C**H_2_CH_3_), 62.42, 62.46 (N**C**H_2_), 115.76, 115.96, 116.18, 125.99, 126.83, 129.32, 129.41, 129.75, 129.84, 130.00, 130.03, 130.19, 130.22, 145.27, 146.66, 147.24, 148.62, 149.45, 150.91 (Ar-**C**), 158.85, 162.31, 164.78, 165.15 (**C**=N, **C**=O), 166.11, 166.18 (**C**=OO). ^31^P-NMR (162 MHz, DMSO-*d*_6_): δ_P_ = −152.97 to −135.41 (m, 1P, **P**F_6_). ^19^F-NMR (377 MHz, DMSO-*d*_6_): δ_F_ = −71.13, −69.24 (2s, 6F, P**F_6_**); (−109.82 to −109.74), (−109.38 to −109.30) (2m, 1F, Ar-**F**). MS (ESI) *m*/*z* = 475.90 [M^+^].

*1-(2-Ethoxy-2-oxoethyl)-4-(2-(4-fluorobenzylidene)hydrazinecarbonyl)pyridin-1-ium tetrafluoroborate* (**9**). It was obtained as brown crystals; m.p.: 88–89 °C. ^1^H-NMR (400 MHz, DMSO-*d*_6_): δ_H_ = 1.28 (t, 3H, *J* = 8 Hz, CH_2_C**H**_3_), 4.24–4.30 (m, 2H, C**H**_2_CH_3_), 5.73 (s, 0.5H, NC**H**_2_), 5.74 (s, 1.5H, NC**H**_2_), 7.25 (t, 0.5H, *J* = 8 Hz, Ar-**H**), 7.37 (dd, 1.5H, *J* = 8 Hz, 12 Hz, Ar-**H**), 7.61 (dd, 0.5H, *J* = 4 Hz, 8 Hz, Ar-**H**), 7.89 (dd, 1.5H, *J* = 4 Hz, 8 Hz, Ar-**H**), 8.17 (s, 0.25H, **H**-C=N), 8.48 (d, 0.5H, *J* = 8 Hz, Ar-**H**), 8.49 (s, 0.75H, **H**-C=N), 8.60 (d, 1.5H, *J* = 8 Hz, Ar-**H**), 9.19 (d, 0.5H, *J* = 4 Hz, Ar-**H**), 9.26 (d, 1.5H, *J* = 4 Hz, Ar-**H**), 12.51 (s, 0.75H, CON**H**), 12.54 (s, 0.25H, CON**H**). ^13^C-NMR (100 MHz, DMSO-*d*_6_): δ_C_ =13.93 (CH_2_**C**H_3_), 60.49, 60.53 (**C**H_2_CH_3_), 62.42, 62.46 (N**C**H_2_), 115.76, 115.96, 116.18, 125.99, 126.83, 129.33, 129.41, 129.75, 129.84, 130.00, 130.03, 130.19, 130.22, 145.27, 146.66, 147.24, 148.62, 149.44, 150.90 (Ar-**C**), 158.85, 162.31, 164.78, 165.15 (**C**=N, **C**=O), 166.11, 166.18 (**C**=OO). ^11^B-NMR (128 MHz, DMSO-*d*_6_): δ_B_ = −1.29 (d, 1B, **B**F_4_). ^19^F-NMR (377 MHz, DMSO-*d*_6_): δ_F_ = (−109.81 to −109.74), (−109.42 to −109.30) (2m, 1F, Ar-**F**); −148.34, −148.28 (2d, 4F, B**F**_4_). MS (ESI) *m*/*z* = 417.90 [M^+^].

*1-(2-Ethoxy-2-oxoethyl)-4-(2-(4-fluorobenzylidene)hydrazinecarbonyl)pyridin-1-ium trifluoroacetate* (**10**). It was obtained as brown crystals; m.p.: 93–94 °C. ^1^H-NMR (400 MHz, DMSO-*d*_6_): δ_H_ = 1.28 (t, 3H, *J* = 8 Hz, CH_2_C**H**_3_), 4.24–4.30 (m, 2H, C**H**_2_CH_3_), 5.73 (s, 0.5H, NC**H**_2_), 5.75 (s, 1.5H, NC**H**_2_), 7.25 (t, 0.5H, *J* = 8 Hz, Ar-**H**), 7.37 (dd, 1.5H, *J* = 8 Hz, 12 Hz, Ar-**H**), 7.61 (dd, 0.5H, *J* = 4 Hz, 8 Hz, Ar-**H**), 7.89 (dd, 1.5H, *J* = 4 Hz, 8 Hz, Ar-**H**), 8.17 (s, 0.25H, **H**-C=N), 8.48 (d, 0.5H, *J* = 8 Hz, Ar-**H**), 8.50 (s, 0.75H, **H**-C=N), 8.60 (d, 1.5H, *J* = 8 Hz, Ar-**H**), 9.19 (d, 0.5H, *J* = 4 Hz, Ar-**H**), 9.27 (d, 1.5H, *J* = 8 Hz, Ar-**H**), 12.51 (s, 0.75H, CON**H**), 12.54 (s, 0.25H, CON**H**). ^13^C-NMR (100 MHz, DMSO-*d*_6_): δ_C_ =13.93 (CH_2_**C**H_3_), 60.49, 60.53 (**C**H_2_CH_3_), 62.42, 62.46 (N**C**H_2_), 115.76, 115.96, 116.18, 125.98, 126.83, 129.33, 129.41, 129.75, 129.84, 130.00, 130.03, 130.19, 130.22, 145.27, 146.66, 147.24, 148.62, 149.46, 150.91 (Ar-**C**), 158.85, 162.31, 164.78, 165.15 (**C**=O), 166.10, 166.17 (**C**=OO). ^19^F-NMR (377 MHz, DMSO-*d*_6_): δ_F_ = −73.60 (s, 3F, C**F_3_**); (−109.82 to −109.74), (−109.40 to −109.31) (2m, 1F, Ar-**F**). MS (ESI) *m*/*z* = 443.90 [M^+^].

*1-(2-(4-Chlorophenyl)-2-oxoethyl)-4-(2-(4-fluorobenzylidene)hydrazinecarbonyl)-pyridin-1-ium hexafluorophosphate* (**11**). It was obtained as yellow crystals; m.p.: 84–85 °C. ^1^H-NMR (400 MHz, DMSO-*d*_6_): δ_H_ = 6.59 (s, 0.5H, NC**H**_2_), 6.61 (s, 1.5H, NC**H**_2_), 7.25 (t, 0.5H, *J* = 8 Hz, Ar-**H**), 7.35 (t, 1.5H, *J* = 8 Hz, Ar-**H**), 7.64 (dd, 0.5H, *J* = 8 Hz, Ar-**H**), 7.78 (d, 2H, *J* = 8 Hz, Ar-**H**), 7.89 (dd, 1.5H, *J* = 4 Hz, Ar-**H**), 8.13 (d, 2H, *J* = 8 Hz, Ar-**H**), 8.22 (s, 0.25H, **H**-C=N), 8.52 (d, 0.5H, *J* = 8 Hz, Ar-**H**), 8.60 (s, 0.75H, **H**-C=N), 8.68 (d, 1.5H, *J* = 8 Hz, Ar-**H**), 9.17 (d, 0.5H, *J* = 8 Hz, Ar-**H**), 9.24 (d, 1.5H, *J* = 4 Hz, Ar-**H**), 12.58 (s, 0.25H, CON**H**), 12.66 (s, 0.75H, CON**H**). ^13^C-NMR (100 MHz, DMSO-*d*_6_): δ_C_ = 66.33, 66.36 (N**C**H_2_), 115.79, 115.96, 116.18, 126.03, 126.86, 129.31, 129.44, 129.72, 129.81, 130.18, 130.24, 132.26, 132.31, 139.61, 145.24, 146.67, 147.22, 148.26, 149.43, 150.60 (Ar-**C**), 158.94, 162.29, 164.77, 165.22 (**C**=N, **C**=O), 189.58, 189.74 (CH_2_**C**=O). ^31^P-NMR (162 MHz, DMSO-*d*_6_) δ_P_ = −157.36 to −131.01 (m, 1P, **P**F_6_). ^19^F-NMR (377 MHz, DMSO-*d*_6_): δ_F_ = −71.14, −69.25 (2s, 6F, P**F_6_**), (−109.92 to −109.84), (−109.44 to −109.36) (2m, 1F, Ar-**F**). MS (ESI) *m*/*z* = 541.90 [M^+^].

*1-(2-(4-Chlorophenyl)-2-oxoethyl)-4-(2-(4-fluorobenzylidene)hydrazinecarbonyl)-pyridin-1-ium tetrafluoroborate* (**12**). It was obtained as yellow crystals; m.p.: 89–90 °C. ^1^H-NMR (400 MHz, DMSO-*d*_6_): δ_H_ = 6.60 (s, 0.5H, NC**H**_2_), 6.62 (s, 1.5H, NC**H**_2_), 7.25 (t, 0.5H, *J* = 8 Hz, Ar-**H**), 7.35 (t, 1.5H, *J* = 8 Hz, Ar-**H**), 7.64 (dd, 0.5H, *J* = 8 Hz, Ar-**H**), 7.78 (d, 2H, *J* = 8 Hz, Ar-**H**), 7.89 (dd, 1.5H, *J* = 4 Hz, Ar-**H**), 8.12 (d, 2H, *J* = 8 Hz, Ar-**H**), 8.21 (s, 0.25H, **H**-C=N), 8.52 (d, 0.5H, *J* = 8 Hz, Ar-**H**), 8.60 (s, 0.75H, **H**-C=N), 8.68 (d, 1.5H, *J* = 8 Hz, Ar-**H**), 9.16 (d, 0.5H, *J* = 8 Hz, Ar-**H**), 9.24 (d, 1.5H, *J* = 4 Hz, Ar-**H**), 12.57 (s, 0.25H, CON**H**), 12.65 (s, 0.75H, CON**H**). ^13^C-NMR (100 MHz, DMSO-*d*_6_): δ_C_ = 66.33, 66.36 (N**C**H_2_), 115.79, 115.96, 116.18, 126.03, 126.86, 129.31, 129.44, 129.72, 129.81, 130.18, 130.24, 132.26, 132.31, 139.61, 145.24, 146.67, 147.22, 148.26, 149.43, 150.60 (Ar-**C**), 158.94, 162.29, 164.77, 165.22 (**C**=N, **C**=O), 189.58, 189.74 (CH_2_**C**=O). ^11^B-NMR (128 MHz, DMSO-*d*_6_): δ_B_ = −1.28 (d, 1B, **B**F_4_). ^19^F-NMR (377 MHz, DMSO-*d*_6_): δ_F_ = (−109.94 to −109.77), (−109.47 to −109.35) (2m, 1F, Ar-**F**), −148.35, −148.30 (2d, 4F, B**F**_4_). MS (ESI) *m*/*z* = 483.90 [M^+^].

*1-(2-(4-Chlorophenyl)-2-oxoethyl)-4-(2-(4-fluorobenzylidene)hydrazinecarbonyl)-pyridin-1-ium trifluoroacetate* (**13**). It was obtained as yellow crystals; m.p.: 97–98 °C. ^1^H-NMR (400 MHz, DMSO-*d*_6_): δ_H_ = 6.59 (s, 0.5H, NC**H**_2_), 6.61 (s, 1.5H, NC**H**_2_), 7.25 (t, 0.5H, *J* = 8 Hz, Ar-**H**), 7.35 (t, 1.5H, *J* = 8 Hz, Ar-**H**), 7.64 (dd, 0.5H, *J* = 8 Hz, Ar-**H**), 7.78 (d, 2H, *J* = 8 Hz, Ar-**H**), 7.89 (dd, 1.5H, *J* = 4 Hz, Ar-**H**), 8.12 (d, 2H, *J* = 8 Hz, Ar-**H**), 8.21 (s, 0.25H, **H**-C=N), 8.52 (d, 0.5H, *J* = 8 Hz, Ar-**H**), 8.60 (s, 0.75H, **H**-C=N), 8.68 (d, 1.5H, *J* = 8 Hz, Ar-**H**), 9.17 (d, 0.5H, *J* = 8 Hz, Ar-**H**), 9.24 (d, 1.5H, *J* = 4 Hz, Ar-**H**), 12.57 (s, 0.25H, CON**H**), 12.65 (s, 0.75H, CON**H**). ^13^C-NMR (100 MHz, DMSO-*d*_6_): δ_C_ = 53.71, 66.36 (N**C**H_2_), 115.79, 115.96, 116.18, 126.03, 126.86, 129.31, 129.44, 129.72, 129.81, 130.18, 130.24, 132.26, 132.31, 139.61, 145.24, 146.67, 147.22, 148.26, 149.43, 150.60 (Ar-**C**), 158.94, 162.29, 164.77, 165.22 (**C**=N, **C**=O), 189.58, 189.74 (CH_2_**C**=O). ^19^F-NMR (377 MHz, DMSO-*d*_6_): δ_F_ = −73.50 (s, 3F, C**F_3_**); (−109.98 to −109.90), (−109.52 to −109.44) (2m, 1F, Ar-**F**). MS (ESI) *m*/*z* = 509.90 [M^+^].

*4-(2-(4-Fluorobenzylidene)hydrazinecarbonyl)-1-(2-(4-nitrophenyl)-2-oxoethyl)-pyridin-1-ium hexafluorophosphate* (**14**). It was obtained as yellow crystals; m.p.: 79–80 °C. ^1^H-NMR (400 MHz, DMSO-*d*_6_): δ_H_ = 6.63 (s, 0.5H, NC**H**_2_), 6.65 (s, 1.5H, NC**H**_2_), 7.22 (t, 0.5H, *J* = 8 Hz, Ar-**H**), 7.33 (t, 1.5H, *J* = 8 Hz, Ar-**H**), 7.62 (dd, 0.5H, *J* = 4 Hz, 8 Hz, Ar-**H**), 7.87 (dd, 1.5H, *J* = 4 Hz, 8 Hz, Ar-**H**), 8.16 (s, 0.25H, **H**-C=N), 8.16–8.20 (m, 2H, Ar-**H**), 8.33–8.36 (m, 2H, Ar-**H**), 8.52 (d, 0.5H, *J* = 4 Hz, Ar-**H**), 8.54 (s, 0.75H, **H**-C=N), 8.68 (d, 1.5H, *J* = 8 Hz, Ar-**H**), 9.13 (d, 0.5H, *J* = 8 Hz, Ar-**H**), 9.21 (d, 1.5H, *J* = 4 Hz, Ar-**H**), 12.48 (s, 0.25H, CON**H**), 12.51 (s, 0.75H, CON**H**). ^13^C-NMR (100 MHz, DMSO-*d*_6_): δ_C_ = 52.27, 66.66 (N**C**H_2_), 115.73, 115.93, 116.15, 123.82, 125.74, 126.71, 129.35, 129.44, 129.68, 129.76, 130.04, 138.07, 130.24, 130.27, 130.59, 134.99, 145.10, 145.88, 146.50, 146.91, 149.41, 150.21 (Ar-**C**), 158.74, 162.27, 164.75, 165.25 (**C**=N, **C**=O), 189.80, 189.95 (CH_2_**C**=O). ^31^P-NMR (162 MHz, DMSO-*d*_6_): δ_P_ = −157.35 to −131.01 (m, 1P, **P**F_6_). ^19^F-NMR (377 MHz, DMSO-*d*_6_): δ_F_ = −71.14, −69.25 (2s, 6F, P**F_6_**); (−109.93 to −109.85), (−109.46 to −109.38) (2m, 1F, Ar-**F**). MS (ESI) *m*/*z* = 552.90 [M^+^].

*4-(2-(4-Fluorobenzylidene)hydrazinecarbonyl)-1-(2-(4-nitrophenyl)-2-oxoethyl)-pyridin-1-ium tetrafluoroborate* (**15**). It was obtained as yellow crystals; m.p.: 84–85 °C. ^1^H-NMR (400 MHz, DMSO-*d*_6_): δ_H_ = 6.63 (s, 0.5H, NC**H**_2_), 6.65 (s, 1.5H, NC**H**_2_), 7.22 (t, 0.5H, *J* = 8 Hz, Ar-**H**), 7.32 (t, 1.5H, *J* = 8 Hz, Ar-**H**), 7.62 (dd, 0.5H, *J* = 4 Hz, 8 Hz, Ar-**H**), 7.87 (dd, 1.5H, *J* = 4 Hz, 8 Hz, Ar-**H**), 8.16 (s, 0.25H, **H**-C=N), 8.17–8.20 (m, 2H, Ar-**H**), 8.32–8.36 (m, 2H, Ar-**H**), 8.52 (d, 0.5H, *J* = 4 Hz, Ar-**H**), 8.54 (s, 0.75H, **H**-C=N), 8.68 (d, 1.5H, *J* = 8 Hz, Ar-**H**), 9.14 (d, 0.5H, *J* = 8 Hz, Ar-**H**), 9.24 (d, 1.5H, *J* = 4 Hz, Ar-**H**), 12.48 (s, 0.25H, CON**H**), 12.56 (s, 0.75H, CON**H**). ^13^C-NMR (100 MHz, DMSO-*d*_6_): δ_C_ = 52.27, 66.66 (N**C**H_2_), 115.22, 115.42, 115.64, 123.31, 125.22, 126.23, 128.84, 128.93, 129.19, 129.27, 129.56, 129.73, 129.76, 130.08, 134.50, 144.61, 145.36, 145.99, 146.47, 148.80, 148.92, 149.71 (Ar-**C**), 158.24, 161.78, 164.25, 164.74 (**C**=N, **C**=O), 189.80, 189.95 (CH_2_**C**=O). ^11^B-NMR (128 MHz, DMSO-*d*_6_): δ_B_ = −1.28 (d, 1B, **B**F_4_). ^19^F-NMR (377 MHz, DMSO-*d*_6_): δ_F_ = (−109.94 to −109.86), (−109.48 to −109.40) (2m, 1F, Ar-**F**); −148.38, −148.33 (2d, 4F, B**F**_4_). MS (ESI) *m*/*z* = 494.90 [M^+^].

*4-(2-(4-Fluorobenzylidene)hydrazinecarbonyl)-1-(2-(4-nitrophenyl)-2-oxoethyl)-pyridin-1-ium trifluoroacetate* (**16**). It was obtained as yellow crystals; m.p.: 89–90 °C. ^1^H-NMR (400 MHz, DMSO-*d*_6_): δ_H_ = 6.63 (s, 0.5H, NC**H**_2_), 6.65 (s, 1.5H, NC**H**_2_), 7.22 (t, 0.5H, *J* = 8 Hz, Ar-**H**), 7.33 (t, 1.5H, *J* = 8 Hz, Ar-**H**), 7.62 (dd, 0.5H, *J* = 4 Hz, 8 Hz, Ar-**H**), 7.87 (dd, 1.5H, *J* = 4 Hz, 8 Hz, Ar-**H**), 8.17 (s, 0.25H, **H**-C=N), 8.16–8.21 (m, 2H, Ar-**H**), 8.31–8.35 (m, 2H, Ar-**H**), 8.52 (d, 0.5H, *J* = 4 Hz, Ar-**H**), 8.54 (s, 0.75H, **H**-C=N), 8.68 (d, 1.5H, *J* = 8 Hz, Ar-**H**), 9.14 (d, 0.5H, *J* = 8 Hz, Ar-**H**), 9.24 (d, 1.5H, *J* = 4 Hz, Ar-**H**), 12.47 (s, 0.25H, CON**H**), 12.55 (s, 0.75H, CON**H**). ^13^C-NMR (100 MHz, DMSO-*d*_6_): δ_C_ = 52.27, 66.66 (N**C**H_2_), 115.22, 115.42, 115.64, 123.31, 125.22, 126.23, 128.84, 128.93, 129.19, 129.27, 129.56, 129.73, 129.76, 130.08, 134.50, 144.61, 145.36, 145.99, 146.47, 148.80, 148.92, 149.71 (Ar-**C**), 158.24, 161.78, 164.25, 164.74 (**C**=N, **C**=O), 189.80, 189.95 (CH_2_**C**=O). ^19^F-NMR (377 MHz, DMSO-*d*_6_): δ_F_ = −73.65 (s, 3F, C**F_3_**); (−110.00 to −109.92), (−109.53 to −109.45) (2m, 1F, Ar-**F**). MS (ESI) *m*/*z* = 520.00 [M^+^].

*4-(2-(4-Fluorobenzylidene)hydrazinecarbonyl)-1-(4-phenoxybutyl)pyridin-1-ium hexafluorophosphate* (**17**). It was obtained as yellow crystals; m.p.: 75–76 °C. ^1^H-NMR (400 MHz, DMSO-*d*_6_): δ_H_ = 1.75–1.83 (m, 2H, C**H**_2_CH_2_N), 2.11–2.21 (m, 2H, C**H**_2_CH_2_O), 4.01–4.05 (m, 2H, C**H**_2_N), 4.78 (t, 2H, *J* = 8 Hz, OC**H**_2_CH_2_), 6.91–6.95 (m, 3H, Ar-**H**), 7.24 (dd, 0.5H, *J* = 8 Hz, 12 Hz, Ar-**H**), 7.29 (t, 2H, *J* = 4 Hz, Ar-**H**), 7.34 (t, 1.5H, *J* = 8 Hz, Ar-**H**), 7.62 (dd, 0.5H, *J* = 4 Hz, 8 Hz, Ar-**H**), 7.89 (dd, 1.5H, *J* = 4 Hz, 8 Hz, Ar-**H**), 8.17 (s, 0.25H, **H**-C=N), 8.41 (d, 0.5H, *J* = 4 Hz, Ar-**H**), 8.53 (s, 0.75H, **H**-C=N), 8.56 (d, 1.5H, *J* = 8 Hz, Ar-**H**), 9.28 (d, 0.5H, *J* = 8 Hz, Ar-**H**), 9.36 (d, 1.5H, *J* = 4 Hz, Ar-**H**), 12.50 (bs, 1H, CON**H**). ^13^C-NMR (100 MHz, DMSO-*d*_6_): δ_C_ = 25.28, 25.33 (**C**H_2_CH_2_N), 27.59, 27.68 (**C**H_2_CH_2_O), 60.72, 60.78 (**C**H_2_N), 66.60, 66.64 (O**C**H_2_), 114.44, 115.74, 115.94, 116.16, 120.56, 126.20, 127.19, 129.44, 129.72, 129.81, 130.06, 130.23, 130.26, 145.12, 145.74, 147.37, 149.43, 149.69 (Ar-**C**), 158.39, 158.72, 162.29, 164.76, 165.16 (**C**=N, **C**=O). ^31^P-NMR (162 MHz, DMSO-*d*_6_): δ_P_ = −157.36 to −131.01 (m, 1P, **P**F_6_). ^19^F-NMR (377 MHz, DMSO-*d*_6_): δ_F_ = −71.13, −69.24 (2s, 6F, P**F_6_**); (−109.92 to −109.84), (−109.43 to −109.35) (2m, 1F, Ar-**F**). MS (ESI) *m*/*z* = 537.00 [M^+^].

*4-(2-(4-Fluorobenzylidene)hydrazinecarbonyl)-1-(4-phenoxybutyl)pyridin-1-ium tetrafluoroborate* (**18**). It was obtained as yellow crystals; m.p.: 80–81 °C. ^1^H-NMR (400 MHz, DMSO-*d*_6_): δ_H_ = 1.74–1.82 (m, 2H, C**H**_2_CH_2_N), 2.11–2.20 (m, 2H, C**H**_2_CH_2_O), 4.01–4.04 (m, 2H, C**H**_2_N), 4.79 (dd, 2H, *J* = 4 Hz, 8 Hz, OC**H**_2_), 6.91–6.94 (m, 3H, Ar-**H**), 7.21 (t, 0.5H, *J* = 8 Hz, Ar-**H**), 7.28 (t, 2H, *J* = 4 Hz, Ar-**H**), 7.36 (dd, 1.5H, *J* = 8 Hz,12 Hz, Ar-**H**), 7.62 (dd, 0.5H, *J* = 4 Hz, 8 Hz, Ar-**H**), 7.88 (dd, 1.5H, *J* = 4 Hz, 8 Hz, Ar-**H**), 8.16 (s, 0.25H, **H**-C=N), 8.40 (d, 0.5H, *J* = 8 Hz, Ar-**H**), 8.51 (s, 0.75H, **H**-C=N), 8.54 (d, 1.5H, *J* = 8 Hz, Ar-**H**), 9.26 (d, 0.5H, *J* = 4 Hz, Ar-**H**), 9.35 (d, 1.5H, *J* = 8 Hz, Ar-**H**), 12.47 (bs, 1H, CON**H**). ^13^C-NMR (100 MHz, DMSO-*d*_6_): δ_C_ = 24.59, 24.64 (**C**H_2_CH_2_N), 26.90, 26.99 (**C**H_2_CH_2_O), 60.06, 60.13 (**C**H_2_N), 65.93, 65.96 (O**C**H_2_CH_2_), 113.77, 115.08, 115.28, 115.50, 119.90, 125.51, 126.52, 128.70, 128.78, 129.07, 129.15, 129.51, 129.54, 144.42, 144.50, 145.04, 146.68, 148.80, 148.99 (Ar-**C**), 157.70, 158.09, 161.62, 164.09, 164.47 (**C**=N, **C**=O). ^11^B-NMR (128 MHz, DMSO-*d*_6_): δ_B_ = −1.29 (d, 1B, **B**F_4_). ^19^F-NMR (377 MHz, DMSO-*d*_6_): δ_F_ = (−109.90 to −109.82), (−109.38 to −109.30) (2m, 1F, Ar-**F**); −148.34, −148.29 (2d, 4F, B**F**_4_). MS (ESI) *m*/*z* = 479.90 [M^+^].

*4-(2-(4-Fluorobenzylidene)hydrazinecarbonyl)-1-(4-phenoxybutyl)pyridin-1-ium trifluoroacetate* (**19**). It was obtained as yellow crystals; m.p.: 87–88 °C. ^1^H-NMR (400 MHz, DMSO-*d*_6_): δ_H_ = 1.75–1.83 (m, 2H, C**H**_2_CH_2_N), 2.11–2.19 (m, 2H, C**H**_2_CH_2_O), 4.01–4.05 (m, 2H, C**H**_2_N), 4.78 (t, 2H, *J* = 8 Hz, OC**H**_2_CH_2_), 6.91–6.95 (m, 3H, Ar-**H**), 7.24 (dd, 0.5H, *J* = 8 Hz, 12 Hz, Ar-**H**), 7.29 (t, 2H, *J* = 8 Hz, *o*-Ar-**H**), 7.34 (t, 1.5H, *J* = 8 Hz, Ar-**H**), 7.62 (dd, 0.5H, *J* = 4 Hz, 8 Hz, Ar-**H**), 7.89 (dd, 1.5H, *J* = 4 Hz, 8 Hz, Ar-**H**), 8.16 (s, 0.25H, **H**-C=N), 8.41 (d, 0.5H, *J* = 8 Hz, Ar-**H**), 8.51 (s, 0.75H, **H**-C=N), 8.55 (d, 1.5H, *J* = 8 Hz, Ar-**H**), 9.28 (d, 0.5H, *J* = 8 Hz, Ar-**H**), 9.37 (d, 1.5H, *J* = 8 Hz, Ar-**H**), 12.45 (bs, 1H, CON**H**). ^13^C-NMR (100 MHz, DMSO-*d*_6_): δ_C_ = 25.28, 25.33 (**C**H_2_CH_2_N), 27.58, 27.68 (**C**H_2_CH_2_O ), 60.71, 60.77 (**C**H_2_N), 66.61, 66.64 (O**C**H_2_), 114.45, 115.75, 115.95, 116.17, 120.56, 126.19, 127.18, 129.37, 129.44, 129.73, 129.82, 130.02, 130.05, 130.22, 145.12, 145.74, 147.38, 149.42 (Ar-**C**), 158.39, 158.74, 162.29, 164.76, 165.16 (**C**=N, **C**=O). ^19^F-NMR (377 MHz, DMSO-*d*_6_): δ_F_ = −73.52 (s, 3F, C**F_3_**); (−109.92 to −109.84), (−109.48 to −109.35) (2m, 1F, Ar-**F**). MS (ESI) *m*/*z* = 505.00 [M^+^].

*1-Benzyl-4-(2-(4-fluorobenzylidene)hydrazinecarbonyl)pyridin-1-ium hexafluorophosphate* (**20**). It was obtained as yellow crystals; m.p.: 70–71 °C. ^1^H-NMR (400 MHz, DMSO-*d*_6_): δ_H_ = 5.94 (s, 2H, NC**H**_2_), 7.22 (t, 0.5H, *J* = 8 Hz, Ar-**H**), 7.36 (dd, 1.5H, *J* = 8 Hz, 12 Hz, Ar-**H**), 7.45–7.50 (m, 3H, Ar-**H**), 7.56–7.59 (m, 2.5H, Ar-**H**), 7.88 (dd, 1.5H, *J* = 4 Hz, 8 Hz, Ar-**H**), 8.15 (s, 0.25H, **H**-C=N), 8.43 (d, 0.5H, *J* = 8 Hz, Ar-**H**), 8.49 (s, 0.75H, **H**-C=N), 8.55 (d, 1.5H, *J* = 8 Hz, Ar-**H**), 9.33 (d, 0.5H, *J* = 8 Hz, Ar-**H**), 9.43 (d, 1.5H, *J* = 8 Hz, Ar-**H**), 12.45 (s, 0.75H, CON**H**), 12.50 (s, 0.25H, CON**H**). ^13^C-NMR (100 MHz, DMSO-*d*_6_): δ_C_ = 63.23, 63.32 (N**C**H_2_), 115.47, 115.66, 115.88, 126.34, 127.29, 128.59, 128.64, 128.99, 129.14, 129.21, 129.45, 129.53, 129.77, 129.93, 129.96, 133.69, 133.73, 144.89, 145.48, 147.59, 149.13, 149.75 (Ar-**C**), 158.48, 162.02, 164.50, 164.72 (**C**=N, **C**=O). ^31^P-NMR (162 MHz, DMSO-*d*_6_): δ_P_ = −157.34 to −131.00 (m, 1P, **P**F_6_). ^19^F-NMR (377 MHz, DMSO-*d*_6_): δ_F_ = −71.11, −69.22 (2s, 6F, P**F_6_**); (−109.83 to −109.75) and (−109.41 to −109.33) (2m, 1F, Ar-**F**). MS (ESI) *m*/*z* = 479.90 [M^+^].

*1-Benzyl-4-(2-(4-fluorobenzylidene)hydrazinecarbonyl)pyridin-1-ium tetrafluoroborate* (**21**). It was obtained as yellow crystals; m.p.: 79–80 °C. ^1^H-NMR (400 MHz, DMSO-*d*_6_): δ_H_ = 5.94 (s, 2H, NC**H**_2_), 7.22 (t, 0.5H, *J* = 8 Hz, Ar-**H**), 7.36 (dd, 1.5H, *J* = 8 Hz, 12 Hz, Ar-**H**), 7.45–7.50 (m, 3H, Ar-**H**), 7.56–7.59 (m, 2.5H, Ar-**H**), 7.88 (dd, 1.5H, *J* = 4 Hz, 8 Hz, Ar-**H**), 8.15 (s, 0.25H, **H**-C=N), 8.43 (d, 0.5H, *J* = 8 Hz, Ar-**H**), 8.48 (s, 0.75H, **H**-C=N), 8.55 (d, 1.5H, *J* = 8 Hz, Ar-**H**), 9.32 (d, 0.5H, *J* = 4 Hz, Ar-**H**), 9.42 (d, 1.5H, *J* = 4 Hz, Ar-**H**), 12.44 (s, 0.75H, CON**H**), 12.50 (s, 0.25H, CON**H**). ^13^C-NMR (100 MHz, DMSO-*d*_6_): δ_C_ = 63.51, 63.61 (N**C**H_2_), 115.74, 115.93, 116.15, 126.60, 127.56, 128.86, 128.92, 129.26, 129.42, 129.49, 129.72, 129.81, 130.04, 130.20, 130.23, 133.95, 134.00, 145.15, 145.75, 147.87, 149.41, 150.03 (Ar-**C**), 158.76, 161.98, 162.30, 164.77, 164.99 (**C**=N, **C**=O). ^11^B-NMR (128 MHz, DMSO-*d*_6_): δ_B_ = −1.26 (d, 1B, **B**F_4_). ^19^F-NMR (377 MHz, DMSO-*d*_6_): δ_F_ = (−109.83 to −109.75) and (−109.41 to −109.33) (2m, 1F, Ar-**F**); −148.30, −148.25 (2d, 4F, B**F**_4_). MS (ESI) *m*/*z* = 421.90 [M^+^].

*1-Benzyl-4-(2-(4-fluorobenzylidene)hydrazinecarbonyl)pyridin-1-ium trifluoroacetate* (**22**). It was obtained as yellow crystals; m.p.: 83–84 °C. ^1^H-NMR (400 MHz, DMSO-*d*_6_): δ_H_ = 5.97 (s, 2H, NC**H**_2_), 7.22 (t, 0.5H, *J* = 8 Hz, Ar-**H**), 7.33 (t, 1.5H, *J* = 8 Hz, Ar-**H**), 7.44–7.49 (m, 3H, Ar-**H**), 7.57–7.60 (m, 2.5H, Ar-**H**), 7.86 (dd, 1.5H, *J* = 4 Hz, 8 Hz, Ar-**H**), 8.18 (s, 0.25H, **H**-C=N), 8.42 (d, 0.5H, *J* = 4 Hz, Ar-**H**), 8.55 (s, 0.75H, **H**-C=N), 8.58 (d, 1.5H, *J* = 4 Hz, Ar-**H**), 9.37 (d, 0.5H, *J* = 8 Hz, Ar-**H**), 9.47 (d, 1.5H, *J* = 8 Hz, Ar-**H**), 12.53 (s, 0.25H, CON**H**), 12.63 (s, 0.75H, CON**H**). ^13^C-NMR (100 MHz, DMSO-*d*_6_): δ_C_ = 63.44, 63.50 (N**C**H_2_), 115.73, 115.94, 116.15, 126.61, 127.53, 128.88, 128.94, 129.23, 129.40, 129.46, 129.68, 129.76, 130.04, 130.24, 130.27, 134.00, 134.04, 145.19, 145.76, 147.80, 149.44, 150.03 (Ar-**C**), 157.75, 158.06, 158.73, 162.27, 164.75, 164.98 (**C**=N, **C**=O). ^19^F-NMR (377 MHz, DMSO-*d*_6_): δ_F_ = −73.51 (s, 3F, C**F_3_**), (−109.86 to −109.79), (−109.46 to −109.38) (2m, 1F, Ar-**F**). MS (ESI) *m*/*z* = 447.00 [M^+^].

*4-(2-(4-Fluorobenzylidene)hydrazinecarbonyl)-1-(2-hydroxyethyl)pyridin-1-ium hexafluorophosphate* (**23**). It was obtained as black crystals; m.p.: 68–69 °C. ^1^H-NMR (400 MHz, DMSO-*d*_6_): δ_H_ = 3.87–3.93 (m, 2H, OC**H**_2_), 4.75 (t, 2H, *J* = 4 Hz, NC**H**_2_), 7.24 (dd, 0.5H, *J* = 4 Hz, 8 Hz, Ar-**H**), 7.33 (t, 1.5H, *J* = 8 Hz, Ar-**H**), 7.62 (dd, 0.5H, *J* = 4 Hz, 8 Hz, Ar-**H**), 7.88 (dd, 1.5H, *J* = 4 Hz, 8 Hz, Ar-**H**), 8.16 (s, 0.25H, **H**-C=N), 8.39 (d, 0.5H, *J* = 4 Hz, Ar-**H**), 8.50 (s, 0.75H, **H**-C=N), 8.52 (d, 1.5H, *J* = 4 Hz, Ar-**H**), 9.15 (d, 0.5H, *J* = 8 Hz, Ar-**H**), 9.24 (d, 1.5H, *J* = 8 Hz, Ar-**H**), 12.46 (s, 1H, CON**H**). ^13^C-NMR (100 MHz, DMSO-*d*_6_): δ_C_ = 60.00 (O**C**H_2_), 63.39, 63.45 (N**C**H_2_), 115.77, 115.95, 116.17, 125.80, 126.93, 129.40, 129.48, 129.73, 129.82, 129.99, 130.02, 130.19, 130.22, 145.18, 145.46, 146.11, 147.50, 147.53, 149.44, 149.70 (Ar-**C**), 158.87, 161.41, 162.27, 164.42, 164.74, 165.23 (**C**=N, **C**=O). ^31^P-NMR (162 MHz, DMSO-*d*_6_): δ_P_ = −152.97 to −135.41 (m, 1P, **P**F_6_). ^19^F-NMR (377 MHz, DMSO-*d*_6_): δ_F_ = −71.11, −69.22 (2s, 6F, P**F_6_**); (−109.87 to −109.76), (−109.40 to −109.35) (2m, 1F, Ar-**F**). MS (ESI) *m*/*z* = 433.90 [M^+^].

*4-(2-(4-Fluorobenzylidene)hydrazinecarbonyl)-1-(2-hydroxyethyl)pyridin-1-ium tetrafluoroborate* (**24**). It was obtained as black crystals; m.p.: 73–74 °C. ^1^H-NMR (400 MHz, DMSO-*d*_6_): δ_H_ = 3.76–3.79 (m, 2H, OC**H**_2_), 4.64 (t, 2H, *J* = 4 Hz, NC**H**_2_), 7.24 (dd, 0.5H, *J* = 4 Hz, 8 Hz, Ar-**H**), 7.33 (t, 1.5H, *J* = 8 Hz, Ar-**H**), 7.62 (dd, 0.5H, *J* = 4 Hz, 8 Hz, Ar-**H**), 7.88 (dd, 1.5H, *J* = 4 Hz, 8 Hz, Ar-**H**), 8.16 (s, 0.25H, **H**-C=N), 8.39 (d, 0.5H, *J* = 4 Hz, Ar-**H**), 8.50 (s, 0.75H, **H**-C=N), 8.52 (d, 1.5H, *J* = 4 Hz, Ar-**H**), 9.15 (d, 0.5H, *J* = 8 Hz, Ar-**H**), 9.24 (d, 1.5H, *J* = 8 Hz, Ar-**H**), 12.46 (s, 1H, CON**H**). ^13^C-NMR (100 MHz, DMSO-*d*_6_): δ_C_ = 60.01 (O**C**H_2_), 63.29, 63.52 (N**C**H_2_), 115.76, 115.95, 116.17, 125.80, 126.93, 129.40, 129.48, 129.73, 129.82, 129.99, 130.02, 130.19, 130.22, 145.18, 145.46, 146.11, 147.50, 147.53, 149.44, 149.70 (Ar-**C**), 158.87, 161.41, 162.27, 164.42, 164.74, 165.23 (**C**=N, **C**=O). ^11^B-NMR (128 MHz, DMSO-*d*_6_): δ_B_ = −1.29 (d, 1B, **B**F_4_). ^19^F-NMR (377 MHz, DMSO-*d*_6_): δ_F_ = (−109.90 to −109.82), (−109.38 to −109.30) (2m, 1F, Ar-**F**); −148.34, −148.29 (2d, 4F, B**F**_4_). MS (ESI) *m*/*z* = 375.90 [M^+^].

*4-(2-(4-Fluorobenzylidene)hydrazinecarbonyl)-1-(2-hydroxyethyl)pyridin-1-ium trifluoroacetate* (**25**). It was obtained as black crystals; m.p.: 79–80 °C. ^1^H-NMR (400 MHz, DMSO-*d*_6_): δ_H_ = 3.87–3.92 (m, 2H, OC**H**_2_), 4.75 (t, 2H, *J* = 4 Hz, NC**H**_2_), 7.23 (t, 0.5H, *J* = 8 Hz, Ar-**H**), 7.34 (dd, 1.5H, *J* = 8 Hz, 12 Hz, Ar-**H**), 7.62 (dd, 0.5H, *J* = 4 Hz, 8 Hz, Ar-**H**), 7.88 (dd, 1.5H, *J* = 4 Hz, 8 Hz, Ar-**H**), 8.16 (s, 0.25H, **H**-C=N), 8.40 (d, 0.5H, *J* = 8 Hz, Ar-**H**), 8.50 (s, 0.75H, **H**-C=N), 8.53 (d, 1.5H, *J* = 8 Hz, Ar-**H**), 9.15 (d, 0.5H, *J* = 4 Hz, Ar-**H**), 9.24 (d, 1.5H, *J* = 8 Hz, Ar-**H**), 12.46 (s, 1H, CON**H**). ^13^C-NMR (100 MHz, DMSO-*d*_6_): δ_C_ = 59.99 (O**C**H_2_), 63.38, 63.44 (N**C**H_2_), 115.77, 115.95, 116.17, 125.47, 126.93, 126.82, 126.91, 129.47, 129.73, 129.82, 130.24, 130.19, 145.19, 145.48, 146.11, 147.56, 149.43, 149.72 (Ar-**C**), 158.88, 162.28, 163.55, 164.76, 165.19 (**C**=N, **C**=O). ^19^F-NMR (377 MHz, DMSO-*d*_6_): δ_F_ = −74.77 (s, 3F, C**F_3_**); (−109.92 to −109.84), (−109.48 to −109.35) (2m, 1F, Ar-**F**). MS (ESI) *m*/*z* = 401.90 [M^+^].

### 3.4. Antifungal Screening

#### 3.4.1. Growth Conditions, Species Identification and Determination of MIC_90_

Yeast extract, peptone and dextrose (YPD) medium (Sigma, Aldrich, St. Louis, MO, USA) was used to culture the yeast strains used in this study. For longer periods, the strains were maintained on YPD agar plates and kept at 4 °C. For experimental purposes, a loop-full of culture was taken from the plates, dissolved in 50 mL of YPD medium and grown on a rotary shaker at 37 °C (150–170 rpm). The culture samples were stored at −80 °C with 500 μL of cell culture and 500 μL of glycerol as glycerol stocks. Identification of the yeasts was done through Gram staining procedure, germ tube test, CHROMagar test and VITEK 2 yeast identification system. 

Minimum inhibitory concentration (MIC_90_) was determined following the NCCLS document M27-A2, 2002 [44]. The cell suspensions and required concentrations of the synthesized compounds were added to different wells of 96 well microtitre plates as done earlier [45,46,47,48,49,50].

#### 3.4.2. Ergosterol Extraction and Estimation Assay

Initially, *Candida* cells and MIC and sub-MIC values of the test agents were added to 50 mL of YPD medium. Both the control and treated samples were incubated for 16 h and each sample was harvested by centrifugation for 5 min at 2700 rpm. After the weighing the pellet, 25% alcoholic potassium hydroxide solution was added to each sample and it was vortexed for 1 min. For sterol extraction, sterile distilled water and *n*-heptane was added to the sample in 1:3 ratio followed by vortexing for 3 min. Sterol extract (20 μL) was then diluted in ethanol (100%) and scanned between 230 and 300 nm. The detailed procedure about the calculation of total ergosterol content in control and treated samples can be obtained from our previous studies [51,52,53,54,55].

#### 3.4.3. Confocal Scanning Laser Microscopy (CSLM)

Initially, *Candida* cells (10^6^ cells/mL) were added to YPD medium. The MIC value of the test agent was added to the medium and the samples were incubated at 37 °C for 16 h. The suspensions were centrifuged at 2700 rpm for 10 min, washed and resuspended in PBS. After this, 5 μL of PI (1 μg/mL) was taken and added to both control and test agent-treated samples. The samples were then incubated again for 30 min at 37 °C. Finally, the control and treated cells were examined under Olympus Laser Confocal Scanning Microscope as done previously [56,57,58].

## 4. Conclusions

This study reports an efficient and ecofriendly ultrasound versus conventional synthesis of novel specific task pyridinium ionic liquid hydrazones tethering fluorinated counter anions. To gain insights into the antifungal mechanism of action, we examined the effect of these synthesized compounds on total ergosterol content present in yeast cell membranes. The results obtained showed a significant decrease in ergosterol content at MIC_90_ values of the active compounds (compounds **8**, **14** and **11**). Thus, we suggest that ergosterol is important as a pharmacological target of the synthesized compounds. Part of this antifungal activity may be originating from the direct binding of these compounds to membrane ergosterol. This may be creating pores in fungal cell membranes, and consequently lead to loss of intracellular content, which ultimately kills the cells as confirmed by our confocal results. Further in vivo studies are necessary to obtain a novel and effective antifungal agent against *Candida* infections.

## Figures and Tables

**Table 1:** Conventional versus ultrasound times and yields of halogenated specific task ionic liquids (ILs) **2**–**7**.

Compound No.	R	Conventional Method CM		Ultrasound Method US	
		Time (h)	Yield (%)	Time (h)	Yield (%)
**2**	CH_2_COOEt	20	90	4	94
**3**	*p*-ClC_6_H_4_COCH_2_	24	91	5	94
**4**	*p*-NO_2_C_6_H_4_COCH_2_	24	80	5	88
**5**	C_6_H_5_O(CH_2_)_4_	24	93	4	96
**6**	C_6_H_5_CH_2_	40	89	6	92
**7**	OH(CH_2_)_2_	48	80	6	88

**Table 2:** Conventional versus ultrasound times and yields of fluorinated specific task-ILs **8**–**25**.

Compound No.	R	Y	Conventional Method CM		Ultrasound Method US	
			Time (h)	Yield (%)	Time (h)	Yield (%)
**8**	CH_2_COOEt	PF_6_	16	88	4	92
**9**	CH_2_COOEt	BF_4_	16	85	4	90
**10**	CH_2_COOEt	COOCF_3_	16	83	4	90
**11**	*p*-ClC_6_H_4_COCH_2_	PF_6_	16	90	4	94
**12**	*p*-ClC_6_H_4_COCH_2_	BF_4_	16	86	4	90
**13**	*p*-ClC_6_H_4_COCH_2_	COOCF_3_	16	92	4	96
**14**	*p*-NO_2_C_6_H_4_COCH_2_	PF_6_	16	89	4	92
**15**	*p*-NO_2_C_6_H_4_COCH_2_	BF_4_	16	84	4	92
**16**	*p*-NO_2_C_6_H_4_COCH_2_	COOCF_3_	16	91	4	94
**17**	C_6_H_5_O(CH_2_)_4_	PF_6_	16	84	5	92
**18**	C_6_H_5_O(CH_2_)_4_	BF_4_	16	84	5	90
**19**	C_6_H_5_O(CH_2_)_4_	COOCF_3_	16	89	5	92
**20**	C_6_H_5_CH_2_	PF_6_	16	94	5	96
**21**	C_6_H_5_CH_2_	BF_4_	16	88	5	92
**22**	C_6_H_5_CH_2_	COOCF_3_	16	86	5	90
**23**	OH(CH_2_)_2_	PF_6_	16	90	5	94
**24**	OH(CH_2_)_2_	BF_4_	16	83	5	90
**25**	OH(CH_2_)_2_	COOCF_3_	16	87	5	92

**Table 3:** Standard *Candida* species and clinical isolates used in this study.

Isolates/Strain	Species (Number of Strains)
Sensitive (standard, *n* = 4)	
ATCC 10261	*C. albicans*
ATCC 90030	*C. glabrata*
ATCC 750	*C. tropicalis*
ATCC 6258	*C. krusei*
Clinical isolates (*n* = 36)	*C. albicans* (**19**), *C. tropicalis* (**5**), *C. glabrata* (**8**), *C. krusei* (**4**)

**Table 4:** Range of MIC_90_ (μg/mL) of the synthesized compounds against four standard and 36 clinical *Candida* isolates.

Test Agents	MIC_90_ Range (μg/mL)
**2**	>1000
**3**	>1000
**4**	>1000
**5**	>1000
**6**	>1000
**7**	500–1000
**8**	62.5–250
**9**	250–500
**10**	250–500
**11**	31.25–62.5
**12**	125–250
**13**	250–500
**14**	62.5–125
**15**	250–500
**16**	500–1000
**17**	125–250
**18**	250–500
**19**	500–1000
**20**	125–250
**21**	250–500
**22**	500–1000
**23**	250–500
**24**	500–1000
**25**	250–500
Fluconazole (standard drug)	1–32

**Table 5:** Percent ergosterol decrease in three standard and three clinical *Candida* isolates grown in presence of MIC and sub-MIC values of compounds **8**, **11** and **14**, respectively.

			% Ergosterol Decrease	
Test Compound	Concentration	Standard Strains (*n* = 3)		Clinical Strains (*n* = 3)
	Control	0		0
**8**	MIC/4	11.6 (± 0.69)		12.9 (± 1.15)
	MIC/2	38.3 (± 3.05)		36.73 (± 2.61)
	MIC	59.06 (± 4.10)		55.23 (± 2.92)
**11**	MIC/4	52.43 (± 3.89)		53.91 (± 3.26)
	MIC/2	65.66 (± 4.93)		62.76 (± 4.27)
	MIC	88.26 (± 2.12)		84.3 (± 5.00)
**14**	MIC/4	36.13 (± 4.00)		29.26 (± 1.16)
	MIC/2	51.16 (± 2.36)		45.72 (± 1.83)
	MIC	70.4 (± 2.62)		71.41 (± 3.16)

## Supplementary Materials

Supplementary File 1

## References

[1] Paluchowska P., Tokarczyk M., Bogusz B., Skiba I., Budak A. (2014). Molecular epidemiology of *Candida albicans* and *Candida glabrata* strains isolated from intensive care unit patients in Poland. Mem. Inst. Oswaldo Cruz..

[2] Rathor N., Khillan V., Sarin S.K. (2014). Nosocomial candiduria in chronic liver disease patients at a hepatobilliary center. Indian J. Crit. Care Med..

[3] Eggimann P., Garbino J., Pittet D. (2003). Epidemiology of Candida species infections in critically ill non-immunosuppressed patients. Lancet Infect. Dis..

[4] Abi-Said D., Anaissie E., Uzun I., Raad O., Pinzcowski H., Vartivarian S. (1997). The epidemiology of hematogenous candidiasis caused by different Candida species. Clin. Infect. Dis..

[5] Pierce G.E. (2005). Pseudomonas aeruginosa, Candida albicans, and device-related nosocomial infections: Implications, trends, and potential approaches for control. J. Ind. Microbiol. Biotechnol..

[6] Laniado-Laborin R., Cabrales-Vargas M.N. (2009). Amphotericin: Side effects and toxicity. Rev. Iberoam. Micol..

[7] Pham C.P., DeFeiter P.W., VanderKuy P.H., VanMook W.N. (2006). Long QTc interval and torsade de pointes caused by fluconazole. Ann. Pharmacother..

[8] Cannon R.D., Lamping E., Holmes A.R., Niimi K., Baret P.V., Keniya M.V., Tanabe K., Niimi M., Goffeau A., Monk B.C. (2009). Efflux-mediated antifungal drug resistance. Clin. Microbiol. Rev..

[9] Odds F.C., Brown A.J., Gow N.A. (2003). Antifungal agents: Mechanisms of action. Trends Microbiol..

[10] Itoga M., Aoki S., Suzuki A., Yoshida Y., Fujinami Y., Masuko M. (2016). Toward resolving anxiety about the accelerated corrosive wear of steel lubricated with the fluorine-containing ionic liquids at elevated temperature. Tribol. Int..

[11] Tiruye G.A., Muñoz-Torrero D., Palma J., Anderson M., Marcilla R. (2016). Performance of solid state supercapacitors based on polymer electrolytes containing different ionic liquids. J. Power Sources.

[12] Andreeva N.A., Chaban V.V. (2016). Amino-functionalized ionic liquids as carbon dioxide scavengers. Ab initio thermodynamics for chemisorption. J. Chem. Thermodyn..

[13] Savest N., Plamus T., Tarasova E., Viirsalu M., Krasnou I., Gudkova V., Küppar K., Krumme A. (2016). The effect of ionic liquids on the conductivity of electrospun polyacrylonitrile membranes. J. Electrost..

[14] Zhai W., Zhu H., Wang L., Liu X., Yang H. (2014). Study of PVDF-HFP/PMMA blended micro-porous gel polymer electrolyte incorporating ionic liquid [BMIM]BF_4_ for Lithium ion batteries. Electrochim. Acta.

[15] Al-Aqmar D.M., Abdelkader H.I., AbouKana M.T.H. (2015). Optical, photo-physical properties and photostability of pyrromethene (PM-597) in ionic liquids as benign green-solvents. J. Lumin..

[16] Poole C.F., Lenca N. (2015). Green sample-preparation methods using room temperature ionic liquids for the chromatographic analysis of organic compounds. Trends Anal. Chem..

[17] Ferreira A.M., Faustino V.F.M., Monda D., Coutinho J.A.P., Freire M.G. (2016). Improving the extraction and purification of immunoglobulin G bythe use of ionic liquids as adjuvants in aqueous biphasic systems. J. Biotechnol..

[18] Shamshina J.L. (2015). Develop ionic liquid drugs. Update regulation to spur research into drugs that the body absorbs more easily and that could reach market more quickly, urge. Nature.

[19] Shirkhanloo H., Ghazaghi M., Mousavi H.Z. (2016). Cadmium determination in human biological samples based on trioctylmethyl ammonium thiosalicylate as a task specific ionic liquid by dispersive liquid–liquid microextraction method. J. Mol. Liq..

[20] Hernoux-Villière A., Lévêque J., Kärkkäinen J., Papaiconomou N., Lajunen M., Lassi U. (2014). Task-specific ionic liquid for the depolymerisation of starch-based industrial waste into high reducing sugars. Catal. Today.

[21] Da Y., Yuan W., Xin T., Nie Y., Ye Y., Yan Y., Liang L., Chen Z. (2012). Synthesis and biological evaluation of new fluorine substituted derivatives as angiotensin II receptor antagonists with anti-hypertension and anti-tumor effects. Bioorg. Med. Chem..

[22] Kirk K.L. (2006). Fluorine in medicinal chemistry: Recent therapeutic applications of fluorinated small molecules. J. Fluor. Chem..

[23] Kirk K.L. (2006). Selective fluorination in drug design and development: An overview of biochemical rationales. Curr. Top. Med. Chem..

[24] Shah P., Westwell A.D. (2007). The role of fluorine in medicinal chemistry. J. Enzyme Inhib. Med. Chem..

[25] Unzner T.A., Magauer T. (2015). Carbon–fluorine bond activation for the synthesis of functionalized molecules. Tetrahedron Lett..

[26] Václavík J., Chernykh Y., Jurásek B., Beier P. (2015). Nucleophilic tetrafluoroethylation of carbonyl compounds with fluorinated sulfones. J. Fluorine Chem..

[27] Rezki N., Al-Sodies S.A., Aouad M.R., Bardaweel S., Messali M., ElAshry E.S.H. (2016). An eco-friendly ultrasound-assisted synthesis ofnovel fluorinated pyridinium salts-based hydrazones and antimicrobial and antitumor screening. Int. J. Mol. Sci..

[28] Shreaz S., Sheikh R.A., Rimple B., Hashmi A.A., Nikhat M., Khan L.A. (2010). Anticandidal activity of cinnamaldehyde, its ligand and Ni(II) complex: Effect of increase in ring and side chain. Microb. Pathog..

[29] Sheikh R.A., Shreaz S., Malik M.A., Khan L.A., Hashmi A.A. (2010). Spectroscopic elucidation of new metal hetroscorpionates: A novel class of antifungal and antibacterial agents. J. Chem. Pharm. Res..

[30] Sheikh R.A., Shreaz S., Sharma G.S., Khan L.A., Hashmi A.A. (2012). Synthesis, characterization and antimicrobial screening of a novel organylborate ligand, potassium hydro (phthalyl)(salicylyl) borate and its Co (II), Ni (II), and Cu (II) complexes. J. Saud. Chem. Soc..

[31] Sheikh R.A., Wani M.Y., Shreaz S., Hashmi A.A. (2016). Synthesis, characterization and biological screening of some Schiff base macrocyclic ligand based transition metal complexes as antifungal agents. Arab. J. Chem..

[32] Sharma G.S., Sheikh R.A., Shreaz S., Hashmi A.A., Khan L.A. (2009). Synthesis, characterization and antimicrobial activity of potassium hydro (benzoyl)(phthalyl) borate and Its Cobalt (II), Nickel (II), and Copper (II) Complexes. Chin. J. Chem..

[33] Shiekh R.A., Shreaz S., Khan L.A., Hashmi A.A. (2010). Development and characterization of bioactive macrocyclic metal complexes, use as a potential drug. J. Chem. Pharm. Res..

[34] Messali M., Aouad M.R., Ali A.A.-S., Rezki N., Ben Hadda T., Hammouti B. (2015). Synthesis, characterization, and POM analysis of novel bioactive imidazolium-based ionic liquids. Med. Chem. Res..

[35] Messali M., Aouad M.R., El-Sayed W.S., Ali A.A.-S., Ben Hadda T., Hammouti B. (2014). New eco-Friendly 1-alkyl-3-(4-phenoxybutyl) imidazolium-based ionic liquids derivatives: A green ultrasound-assisted synthesis, characterization, antibacterial activity and POM analyses. Molecules.

[36] Messali M., Almtiri M.N., Abderrahman B., Salghi R., Aouad M.R., Alshahateet S.F., Ali A.A.-S. (2015). New pyridazinium-based ionic liquids: An eco-friendly ultrasound-assisted synthesis, characterization and biological activity. S. Afr. J. Chem..

[37] Chatel G., MacFarlane D.R. (2014). Ionic liquids and ultrasound in combination: Synergies and challenges. Chem. Soc. Rev..

[38] Marullo S., D’Anna F., Rizzo C., Noto R. (2015). The ultrasounds–ionic liquids synergy on the copper catalyzed azide–alkyne cycloaddition between phenylacetylene and 4-azidoquinoline. Ultrason. Sonochem..

[39] Messali M. (2015). Conventional vs. ultrasound and microwave assisted synthesis: Some new environmentally friendly functionalized picolinium-based ionic liquids with potential antibacterial activity. Acta Pharm..

[40] Rezki N., Al-Yahyawi A.M., Bardaweel S.K., Al-Blewi F.F., Aouad M.R. (2015). Synthesis of novel 2,5-disubstituted-1,3,4-thiadiazoles clubbed 1,2,4-triazole, 1,3,4-thiadiazole, 1,3,4-oxadiazole and/or schiff base as potential antimicrobial and antiproliferative agents. Molecules.

[41] Czub J., Baginski M. (2006). Comparative molecular dynamics study of lipid membranes containing cholesterol and ergosterol. Biophys. J..

[42] de Kroon A.I., Rijken P.J., De Smet C.H. (2013). Checks and balances in membrane phospholipid class and acyl chain homeostasis, the yeast perspective. Prog. Lipid Res..

[43] Ghannoum M.A., Rice L.B. (1999). Antifungal agents: mode of action, mechanisms of resistance, and correlation of these mechanisms with bacterial resistance. Clin. Microbiol. Rev..

[44] National Committee on Clinical Laboratory Standards (NCCLS) (2002). Reference Method for Broth Dilution Antifungal Susceptibility Testing of Yeasts.

[45] Shreaz S., Sheikh R.A., Bhatia R., Neelofar K., Imran S., Hashmi A.A., Manzoor N., Basir S.F., Khan L.A. (2011). Antifungal activity of α-methyl trans cinnamaldehyde, its ligand and metal complexes: Promising growth and ergosterol inhibitors. Biometals.

[46] Shreaz S., Bhatia R., Khan N., Muralidhar S., Manzoor N., Khan L.A. (2013). Influences of cinnamicaldehydes on H^+^ extrusion activity and ultrastructure of Candida. J. Med. Microbiol..

[47] Khan N., Shreaz S., Bhatia R., Ahmad S.I., Muralidhar S., Manzoor N., Khan L.A. (2012). Anticandidal activity of curcumin and methyl cinnamaldehyde. Fitoterapia.

[48] Bharathi N.P., Alam M., Shreaz S., Hashmi A.A. (2009). Synthesis, characterization and biological studies of oil based tin polymer. J. Inorg. Organomet. Polym. Mat..

[49] Bharathi N.P., Khan N.U., Lam M.A., Shreaz S., Hashmi A.A. (2010). Cadmium incorporated oil based bioactive polymers: Synthesis, characterization and physico-chemical studies. J. Inorg. Organom. Pol. Mat..

[50] Shreaz S., Bhatia R., Khan N., Maurya I.K., Ahmad S.I., Muralidhar S., Manzoor N., Khan L.A. (2012). Cinnamic aldehydes affect hydrolytic enzyme secretion and morphogenesis in oral *Candida* isolates. Microb. Pathog..

[51] Irshad M., Shreaz S., Manzoor N., Khan L.A., Rizvi M.M.A. (2011). Anticandidal activity of *Cassia fistula* and its effect on ergosterol biosynthesis. Pharm. Biol..

[52] Shreaz S., Bhatia R., Khan N., Muralidhar S., Basir S.F., Manzoor N., Khan L.A. (2011). Exposure of Candida to p-anisaldehyde inhibits its growth and ergosterol biosynthesis. J. Gen. Appl. Microbiol..

[53] Neelofar K., Shreaz S., Rimple B., Muralidhar S., Nikhat M., Khan L.A. (2011). Curcumin as a promising anticandidal of clinical interest. Can. J. Microbiol..

[54] Vaseem R., Sheikh S., Jawad M.B., Weqar A.S. (2016). In vitro effect of ethanolic extract of *Curcuma longa* rhizome on growth and sterol synthesis in different *Candida* isolates. J. Pure Appl. Microbio..

[55] Shreaz S., Bhatia R., Khan N., Muralidhar S., Basir S.F., Manzoor N., Khan L.A. (2011). Spice oil cinnamaldehyde exhibits potent anticandidal activity against fluconazole resistant clinical isolates. Fitoterapia.

[56] Vaseem R., Shreaz S., Siddiqui W.A. (2014). *Curcuma longa* rhizome extract as a promising source of anticandidal agents. Adv. Sci. Lett..

[57] Shreaz S., Shiekh R.A., Raja V., Wani W.A., Behbehani J.M. (2016). Impaired ergosterol biosynthesis mediated fungicidal activity of Co(II) complex with ligand derived from cinnamaldehyde. Chem. Biol. Int..

[58] Raja V., Ahmad S.I., Irshad M., Wani W.A., Siddiqi W.A., Shreaz S. (2017). Anticandidal activity of ethanolic root extract of *Juglans regia* (L.): Effect on growth, cell morphology, and key virulence factors. J. Mycol. Med..

