# Supplementary material for: Green Ultrasound versus Conventional Synthesis and Characterization of Specific Task Pyridinium Ionic Liquid Hydrazones Tethering Fluorinated Counter Anions: Novel Inhibitors of Fungal Ergosterol Biosynthesis
Source: Molecules. 2017 Nov 7;22(11):1532. doi: 10.3390/molecules22111532 (PMC6150352; doi:10.3390/molecules22111532)
Supplement: Supplementary File 1 [file molecules-22-01532-s001.pdf]

Green ultrasound versus conventional synthesis and characterization of specific task pyridinium ionic liquid hydrazones tethering fluorinated counter anions: Novel inhibitors of fungal ergosterol biosynthesis

Nadjet Rezki[1,2,]*, Salsabeel A. Al-Sodies[1], Sheikh Shreaz[3], Rayees Ahmad Shiekh[1], Mouslim Messali[1], Mohamed R. Aouad[1,2,*]

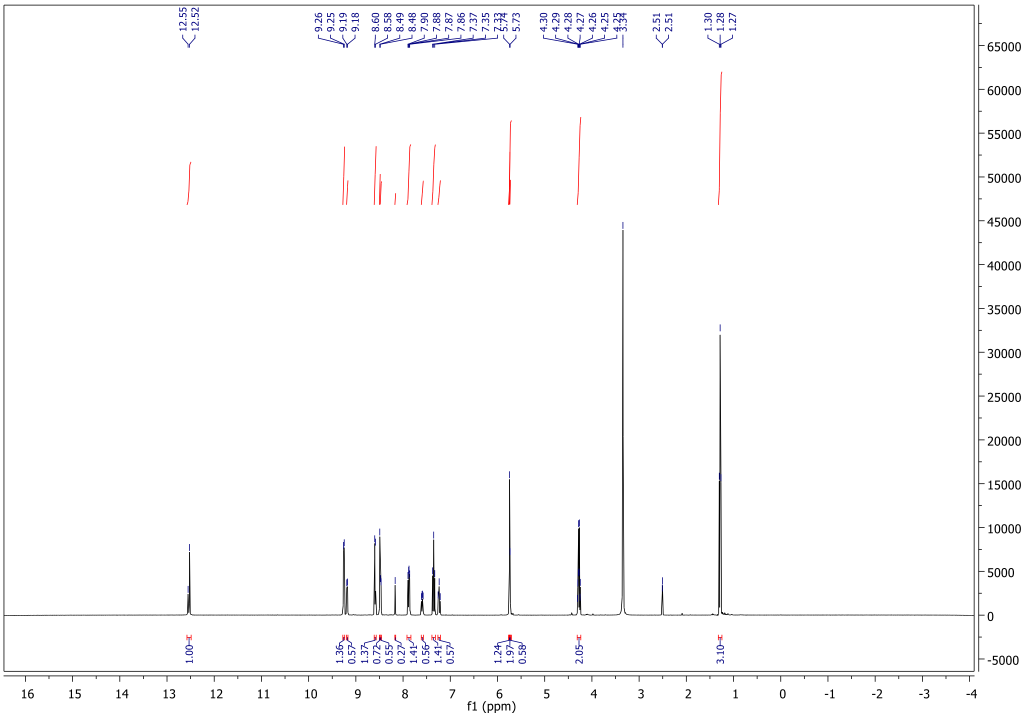

Figure S1. ^{1}H NMR of Compound **2**.

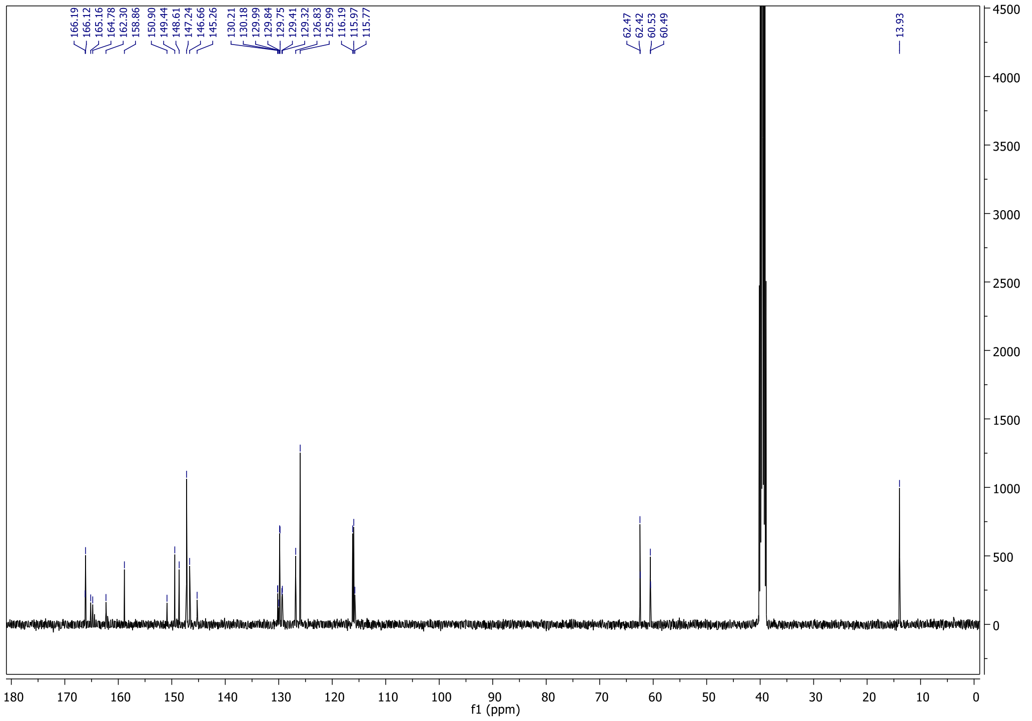

Figure S2. ^{13}C NMR of Compound 2

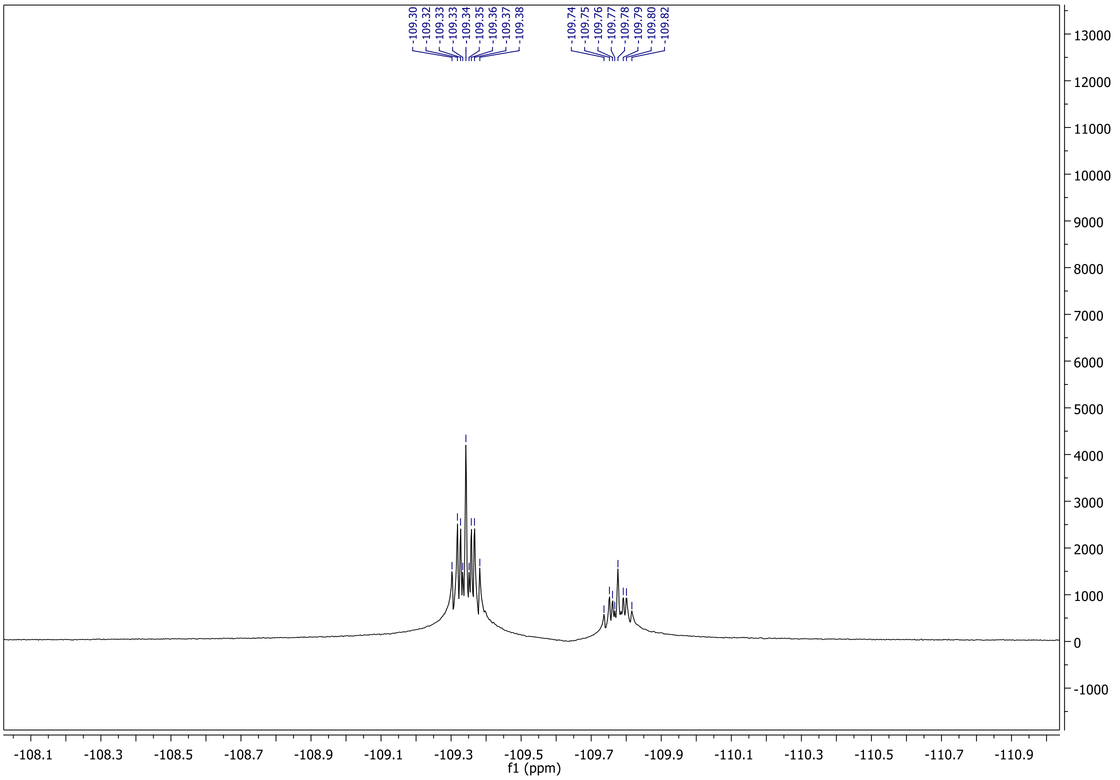

Figure S3. ^{19}FNMR of Compound 2

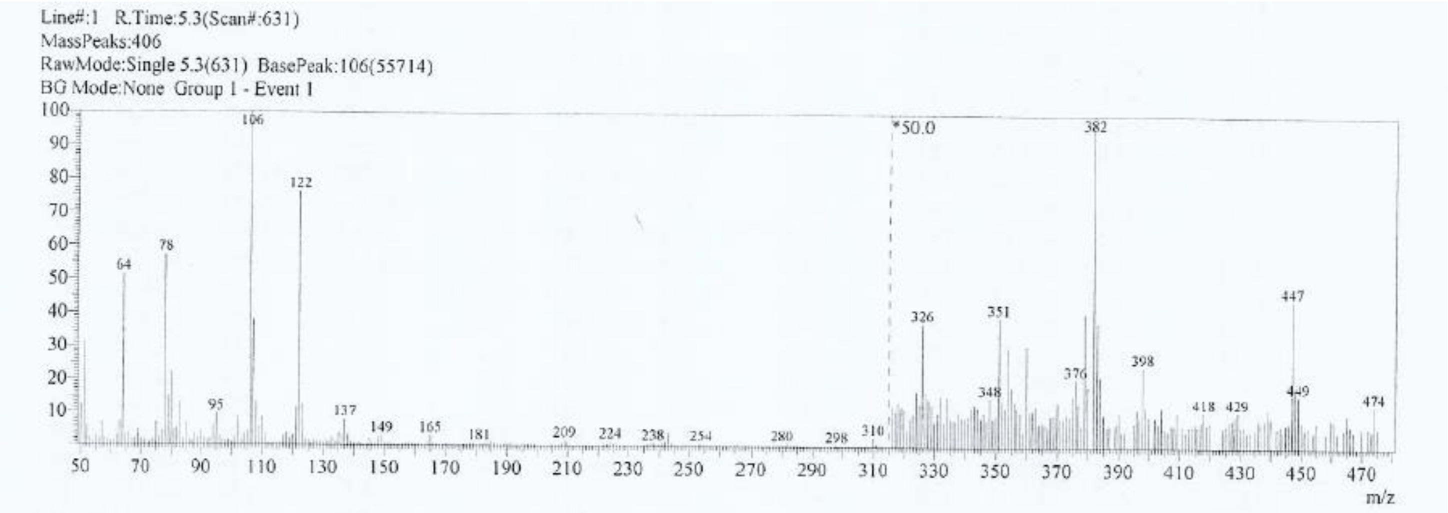

Figure S4. MS (ESI) of Compound 2

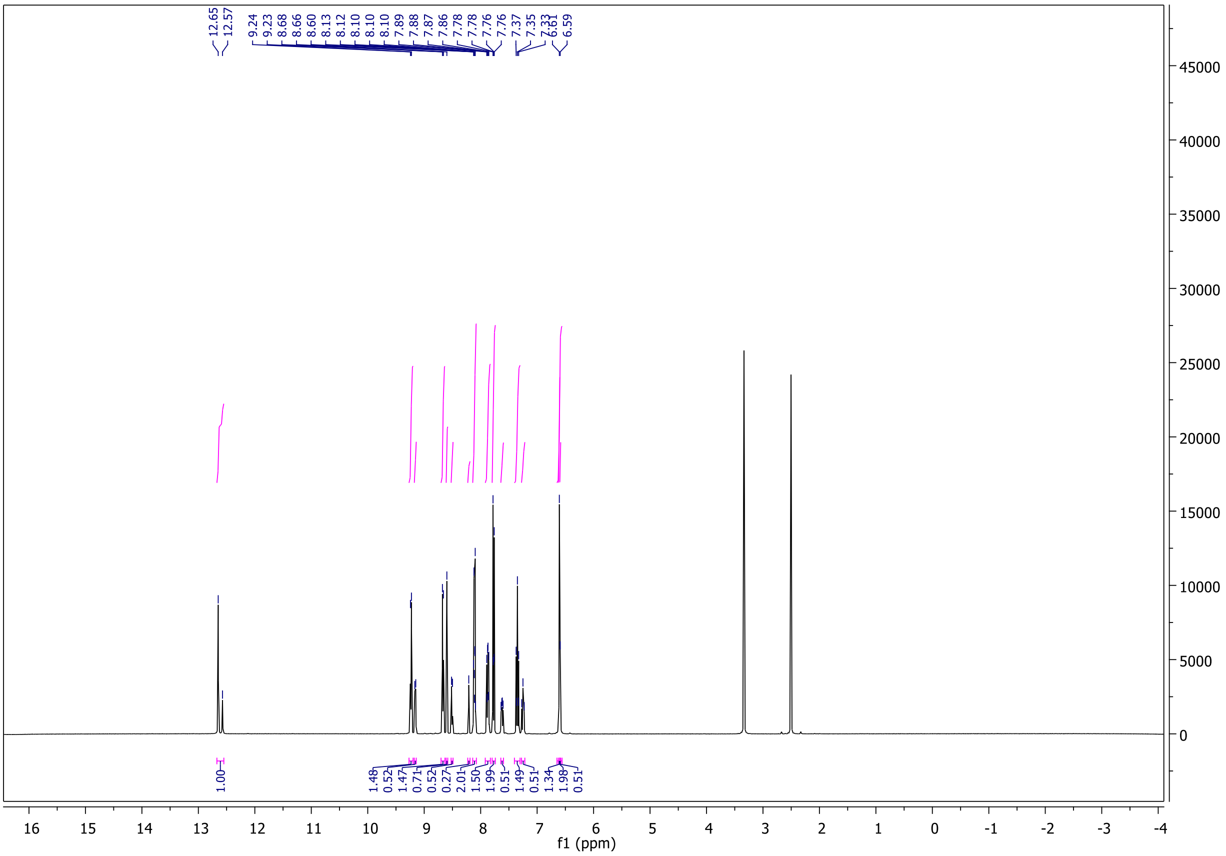

Figure S5. ^{1}HNMR of Compound 3

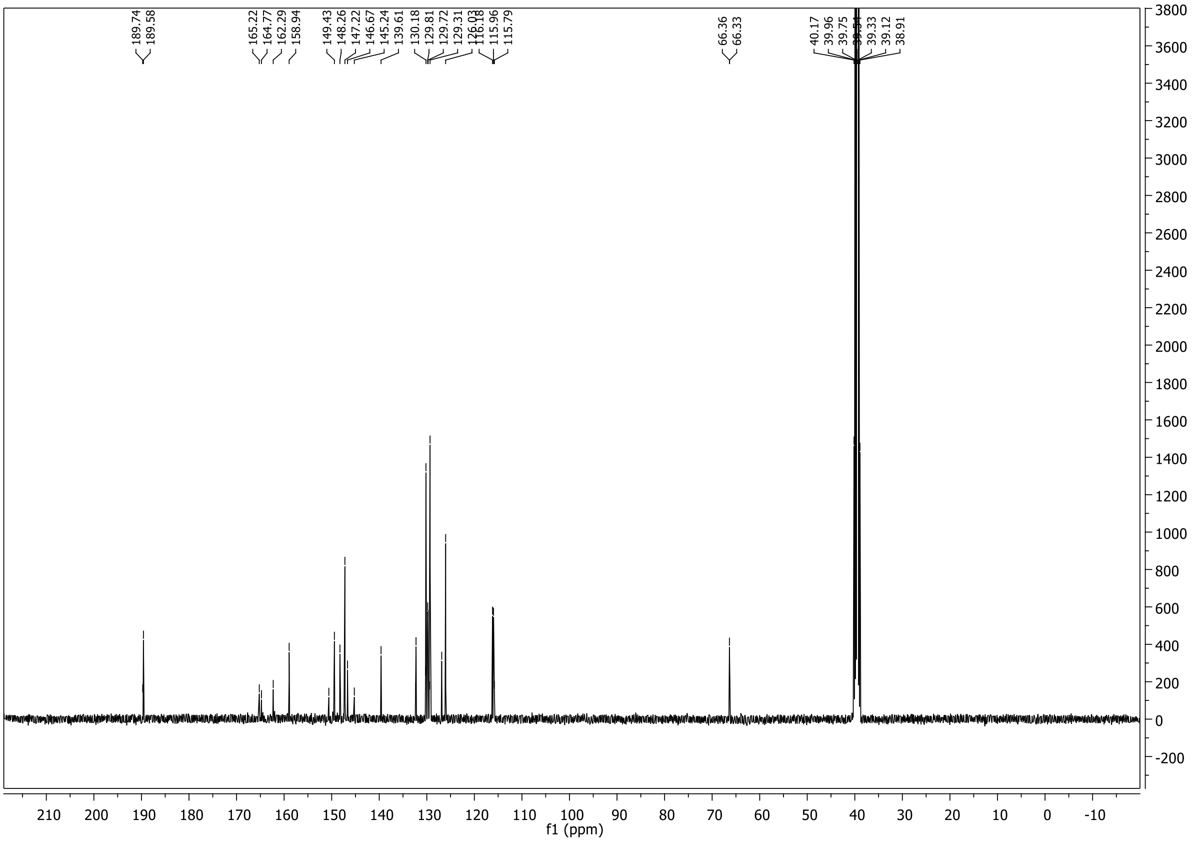

Figure S6. ^{13}CNMR of Compound 3

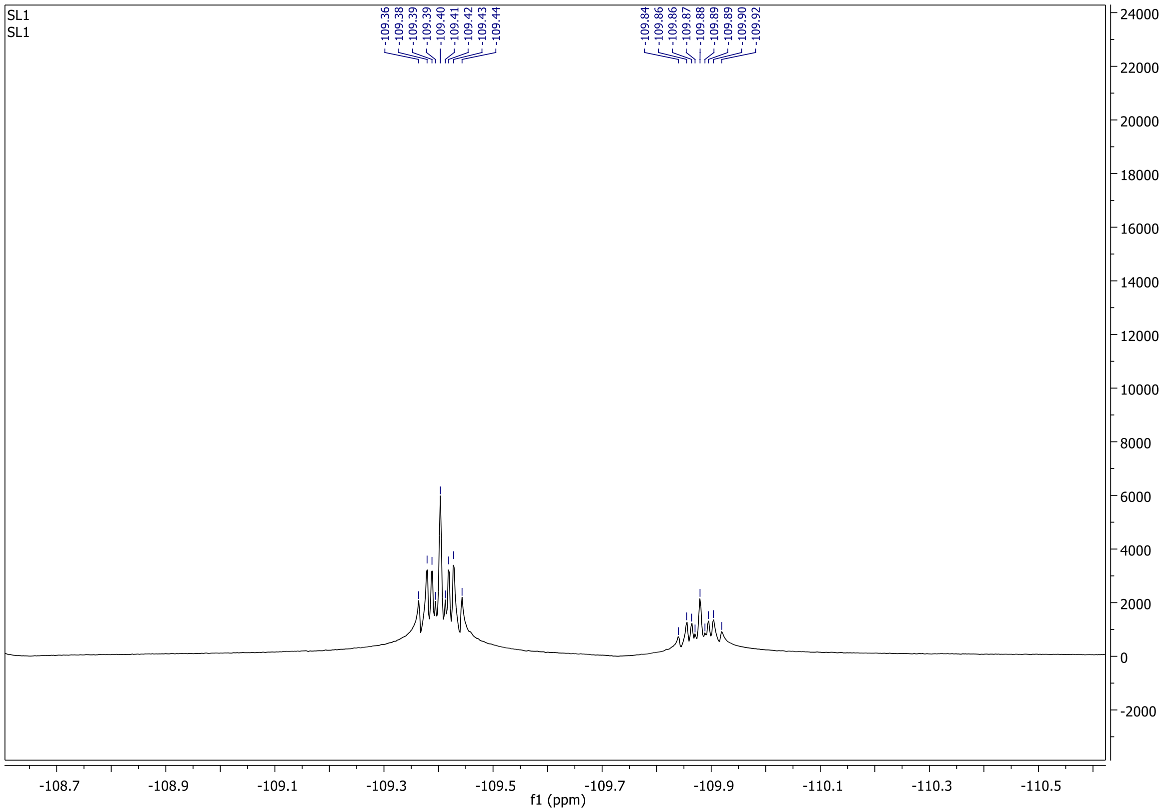

Figure S7. ^{19}FNMR of Compound 3

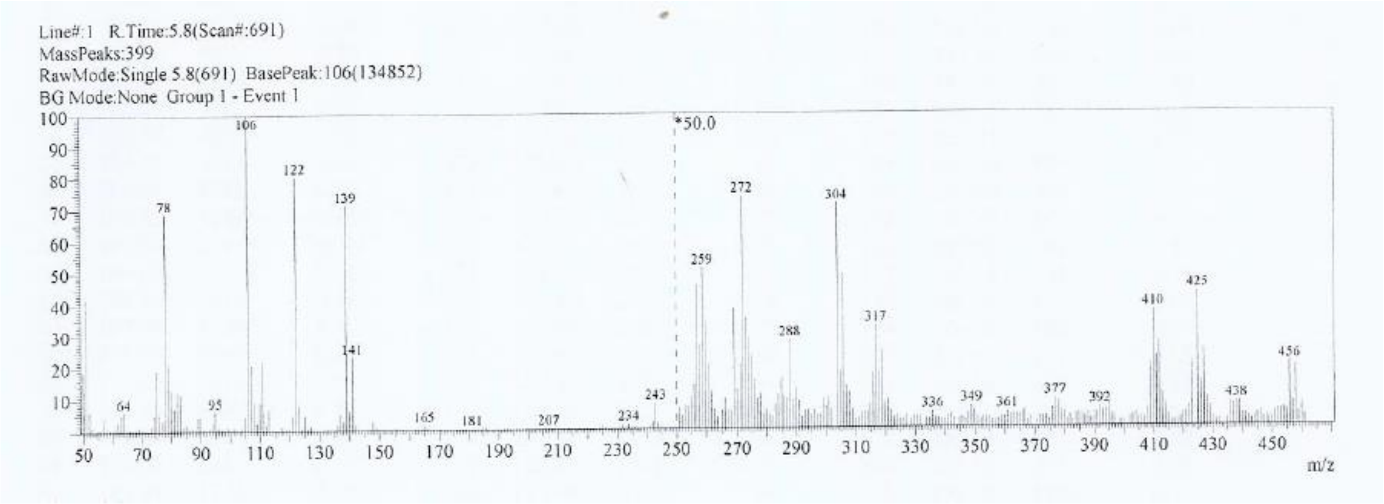

Figure S8. MS (ESI) of Compound 3

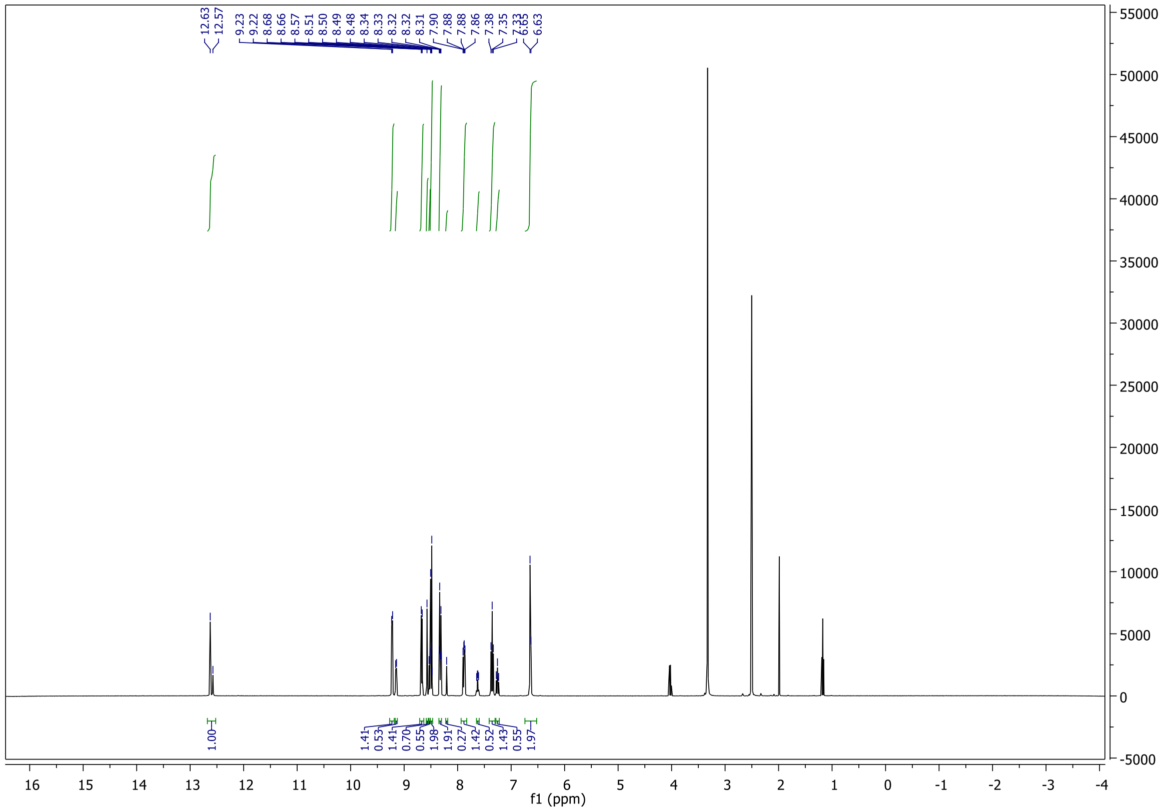

Figure S9. ^{1}HNMR of Compound 4

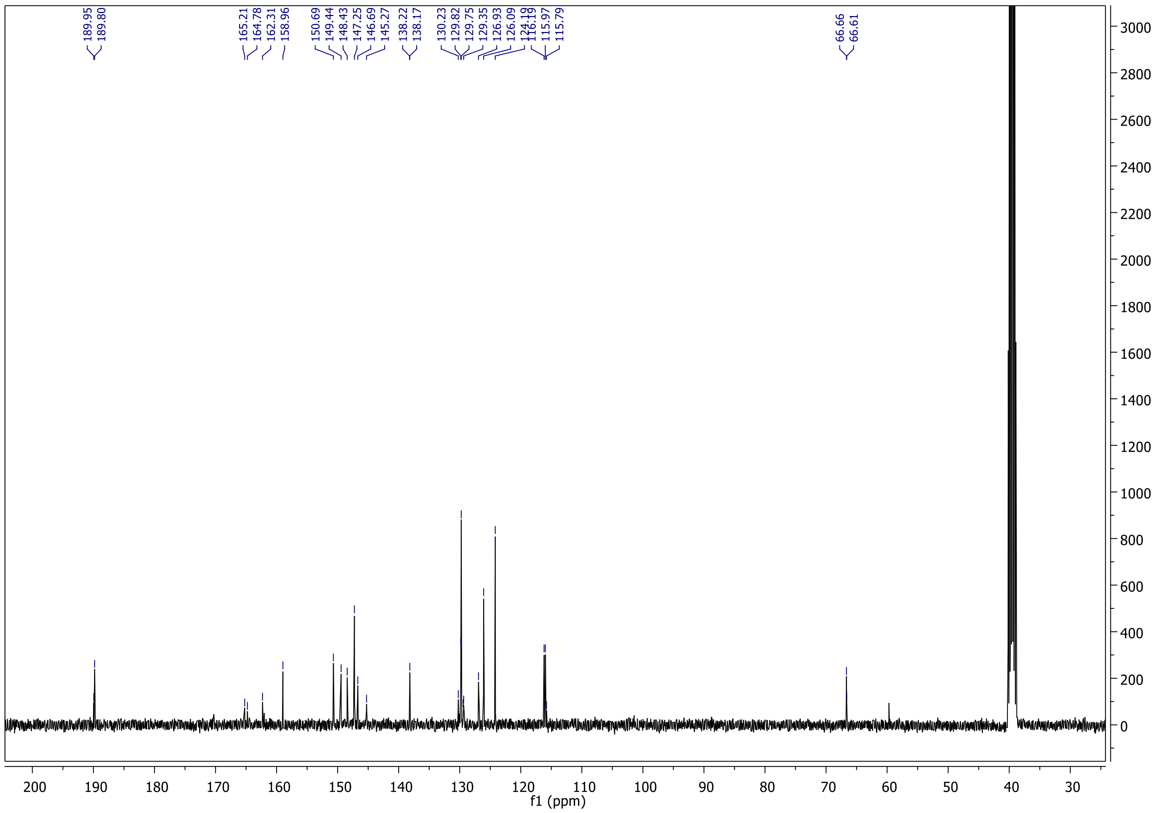

Figure S10. ^{13}CNMR of Compound 4

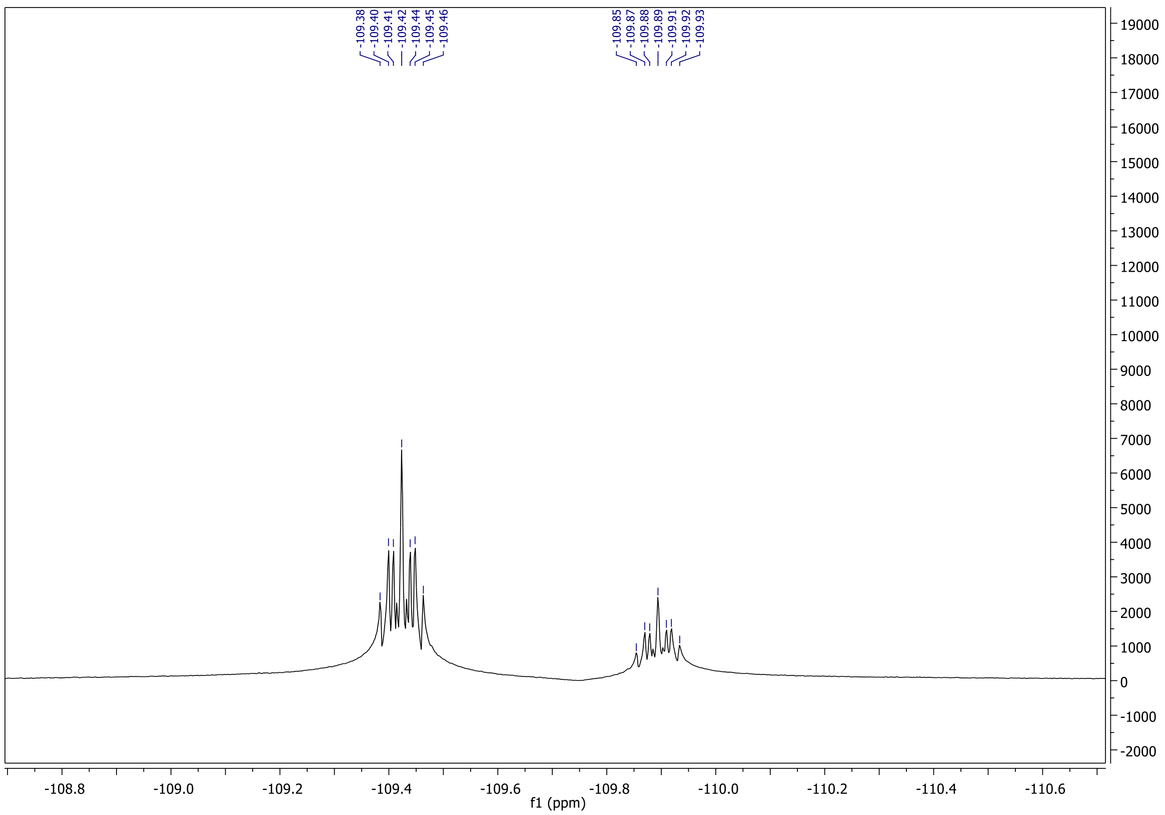

Figure S11. ^{19}FNMR of Compound 4

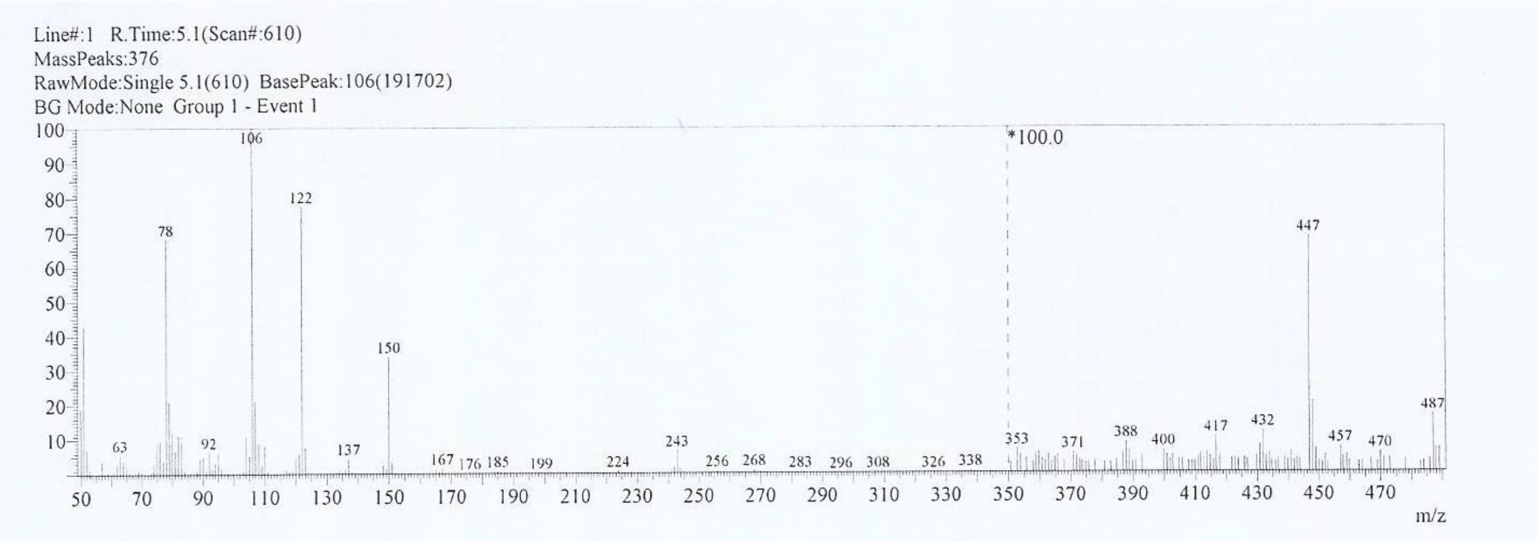

Figure S12. MS (ESI) of Compound 4

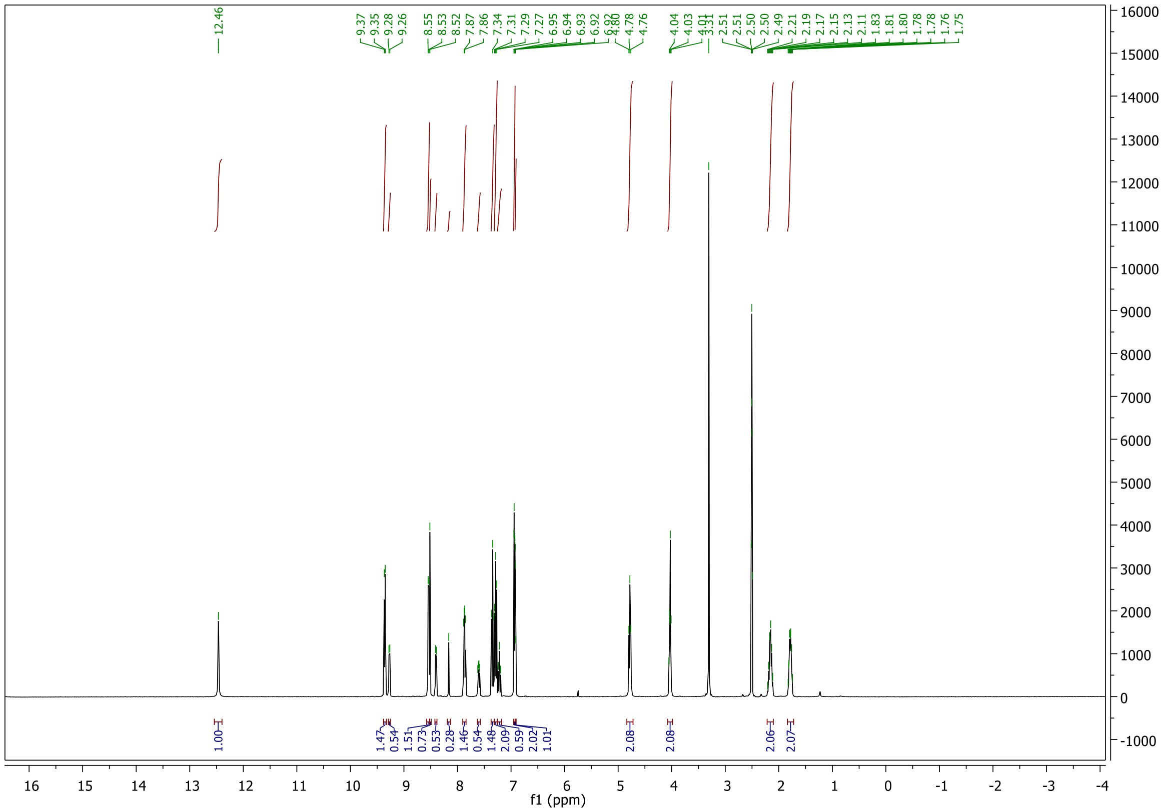

Figure S13. ^{1}HNMR of Compound 5

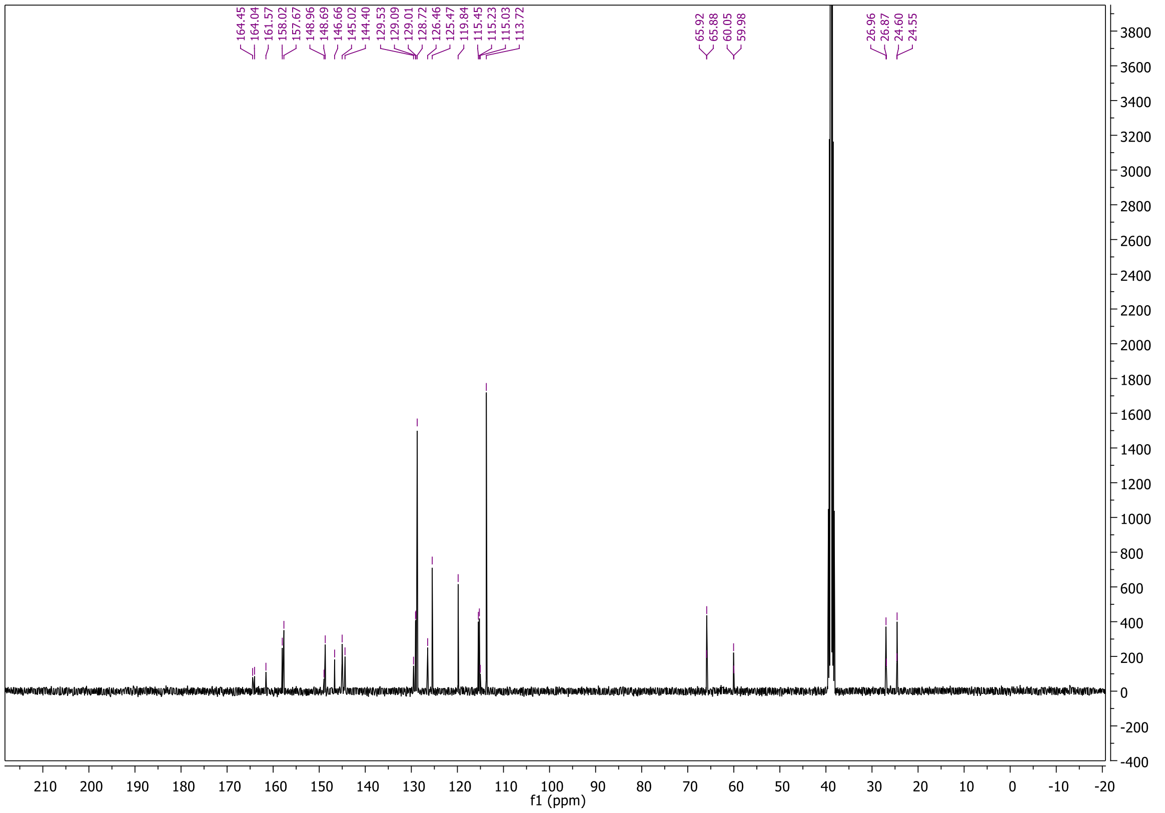

Figure S14. ^{13}CNMR of Compound 5

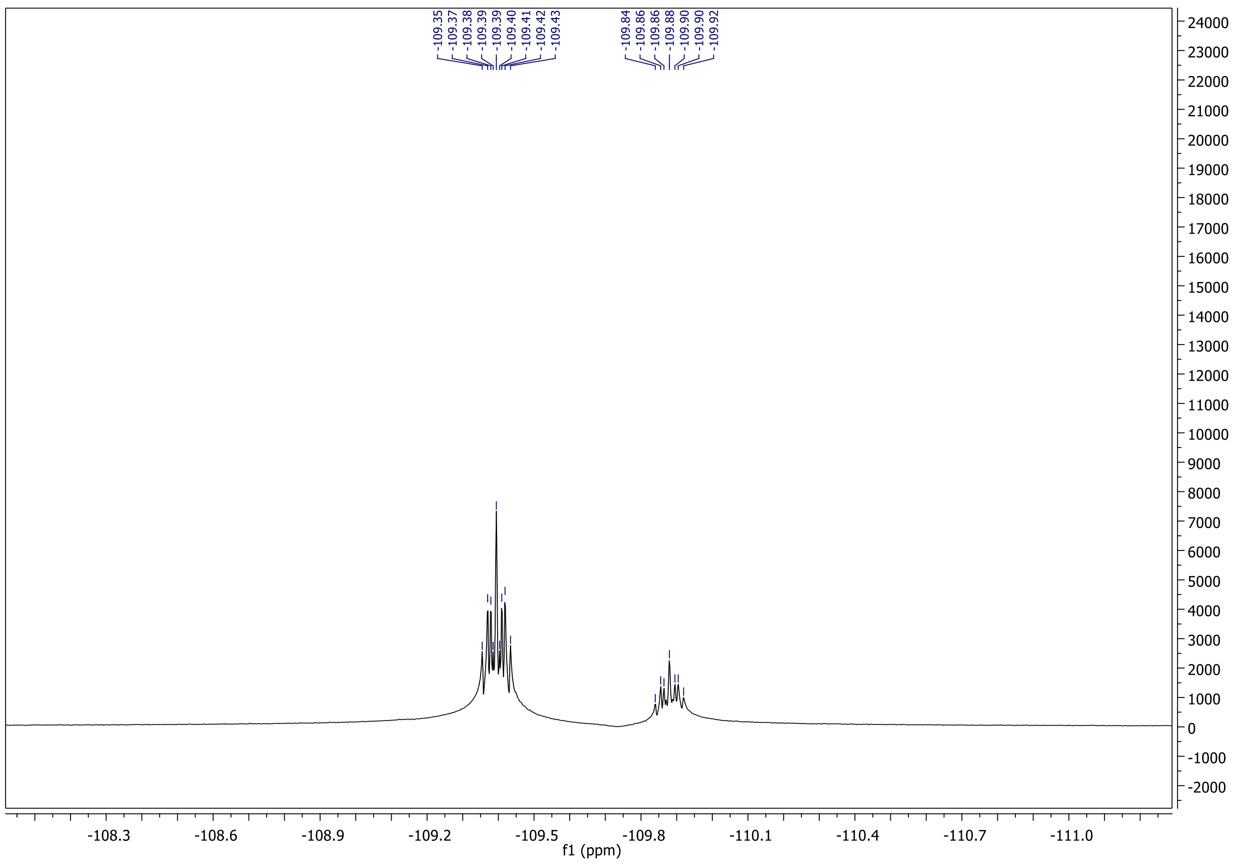

Figure S15. ^{19}FNMR of Compound 5

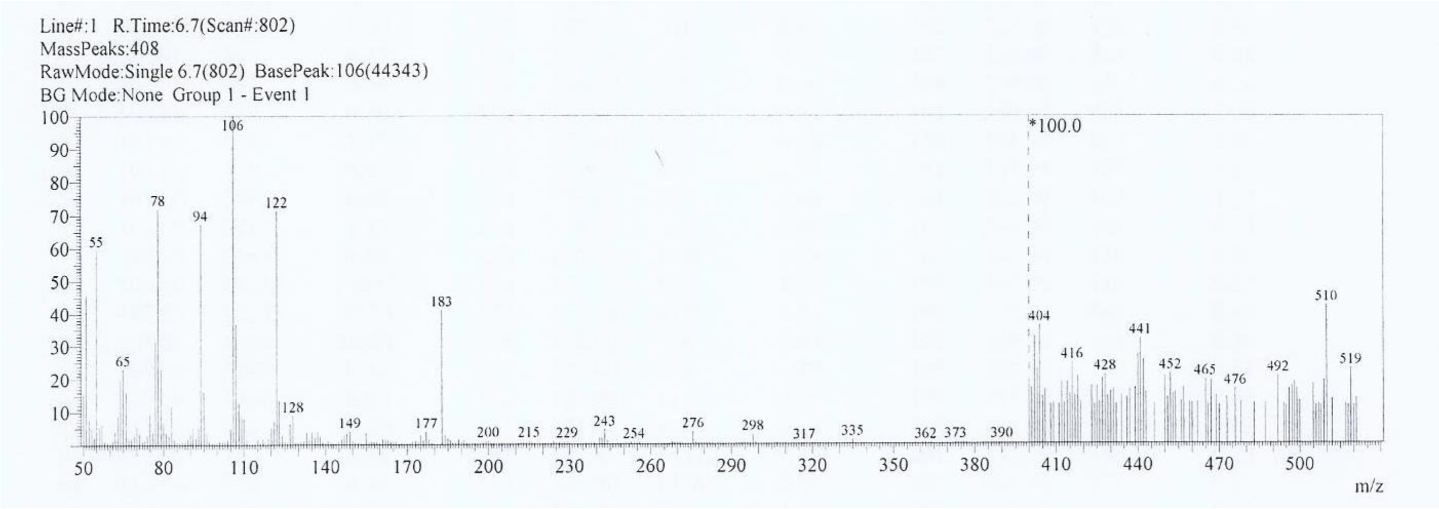

Figure S16. MS (ESI) of Compound 5

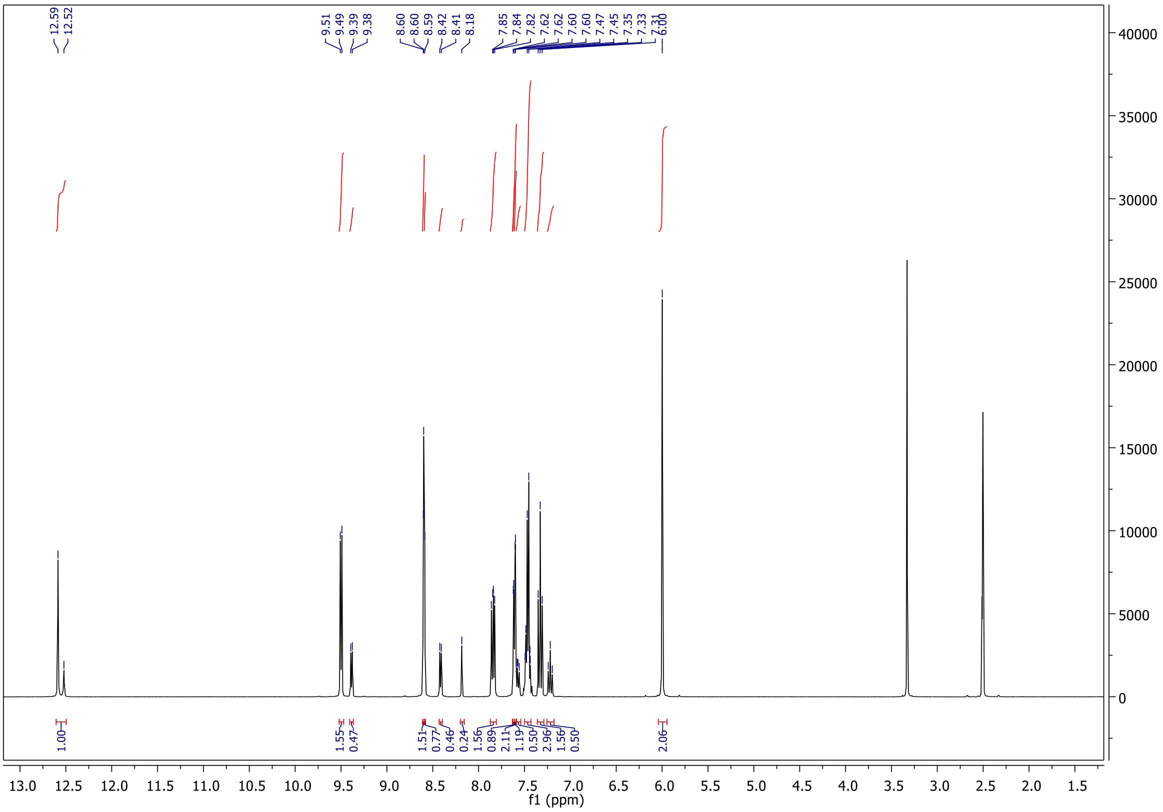

Figure S17. ¹HNMR of Compound 6

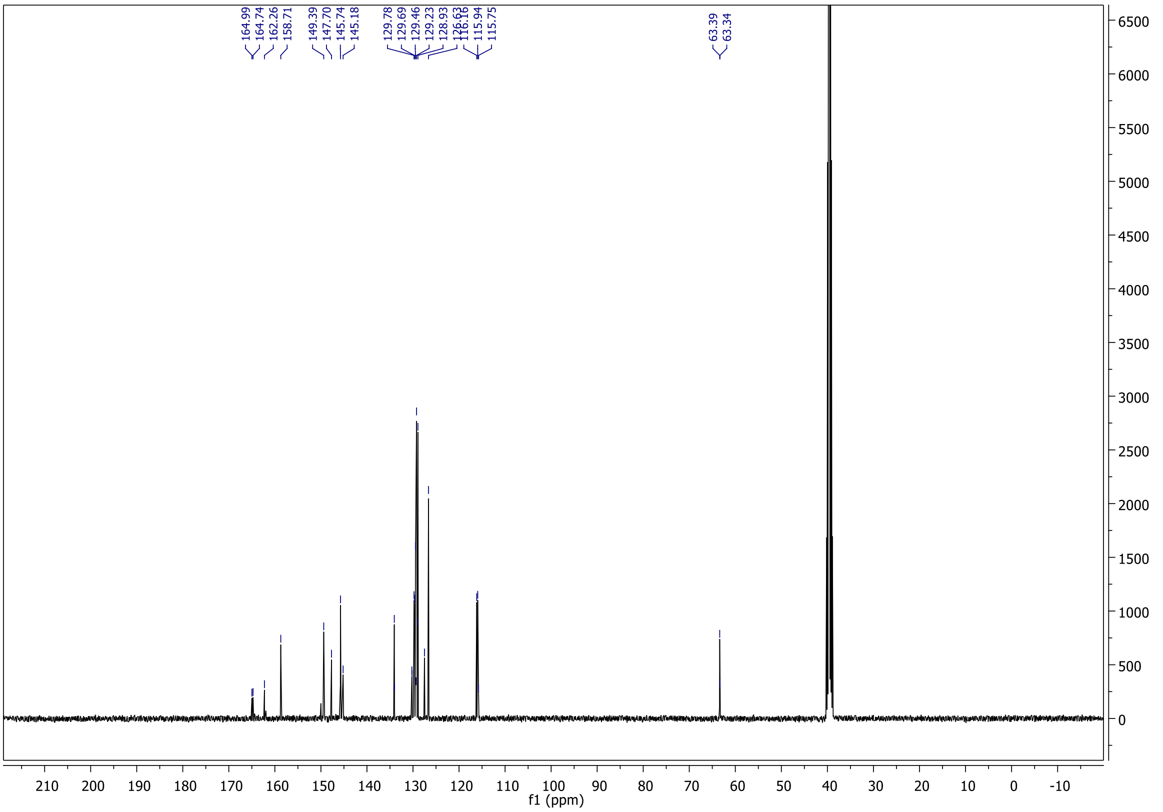

Figure S18. ¹³CNMR of Compound 6

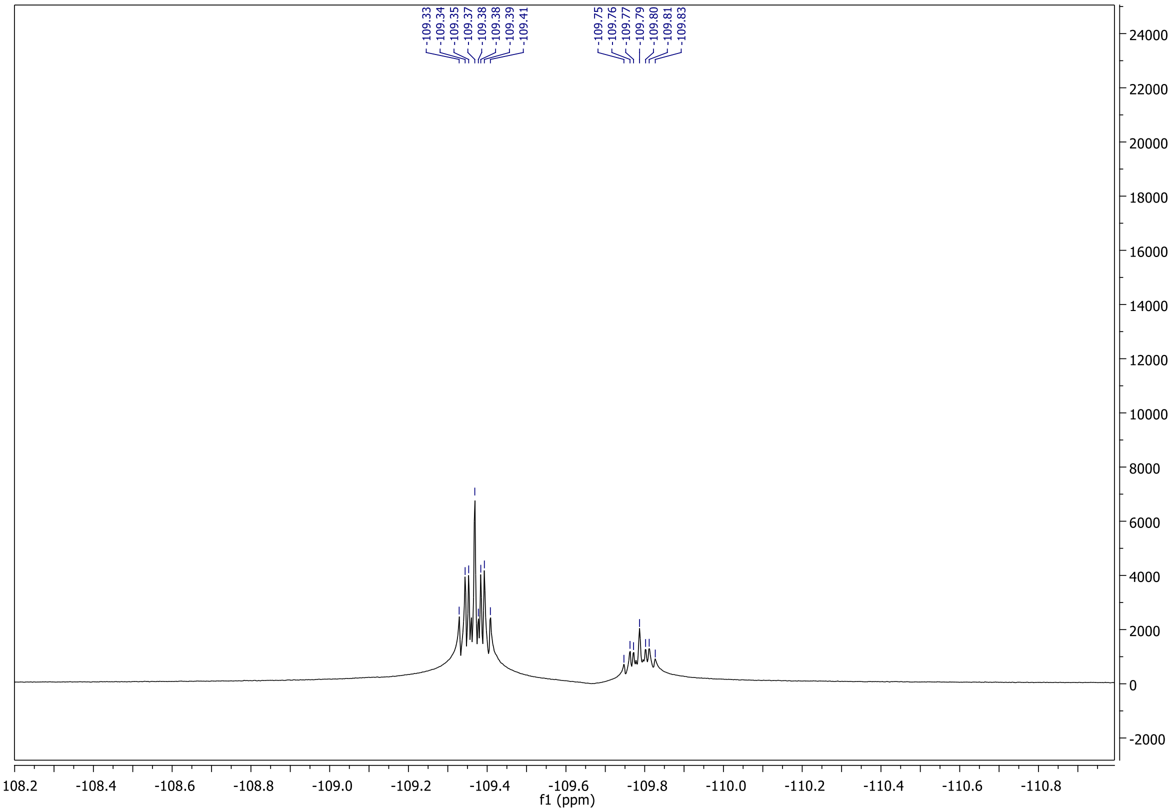

Figure S19. ^{19}FNMR of Compound 6

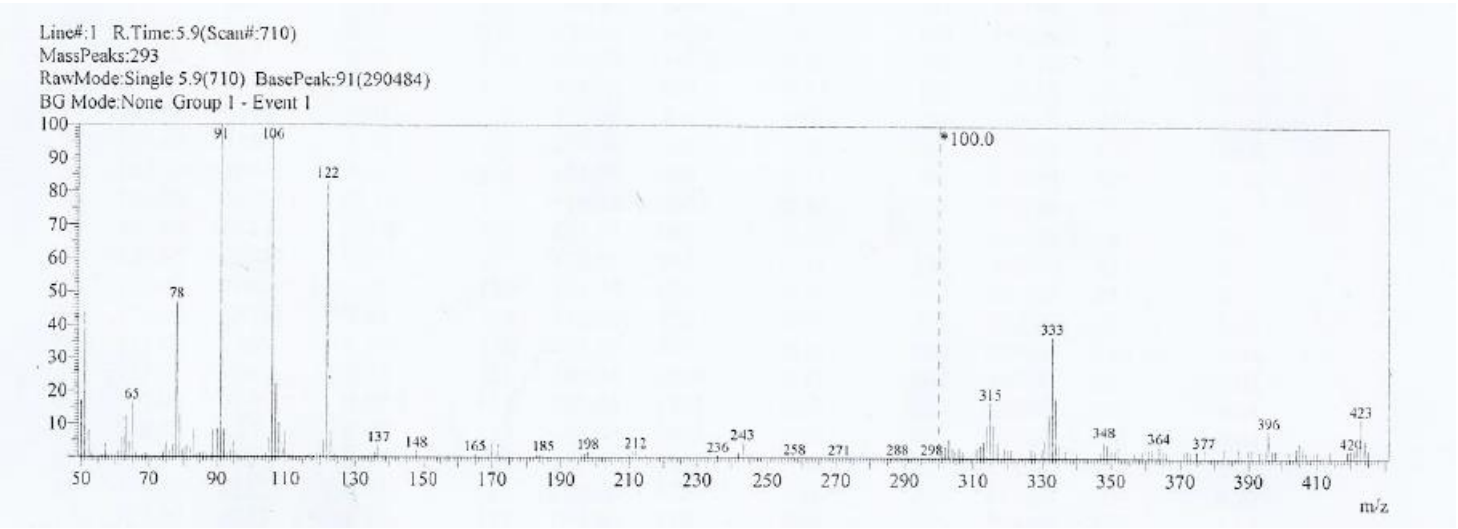

Figure S20. MS (ESI) of Compound 6

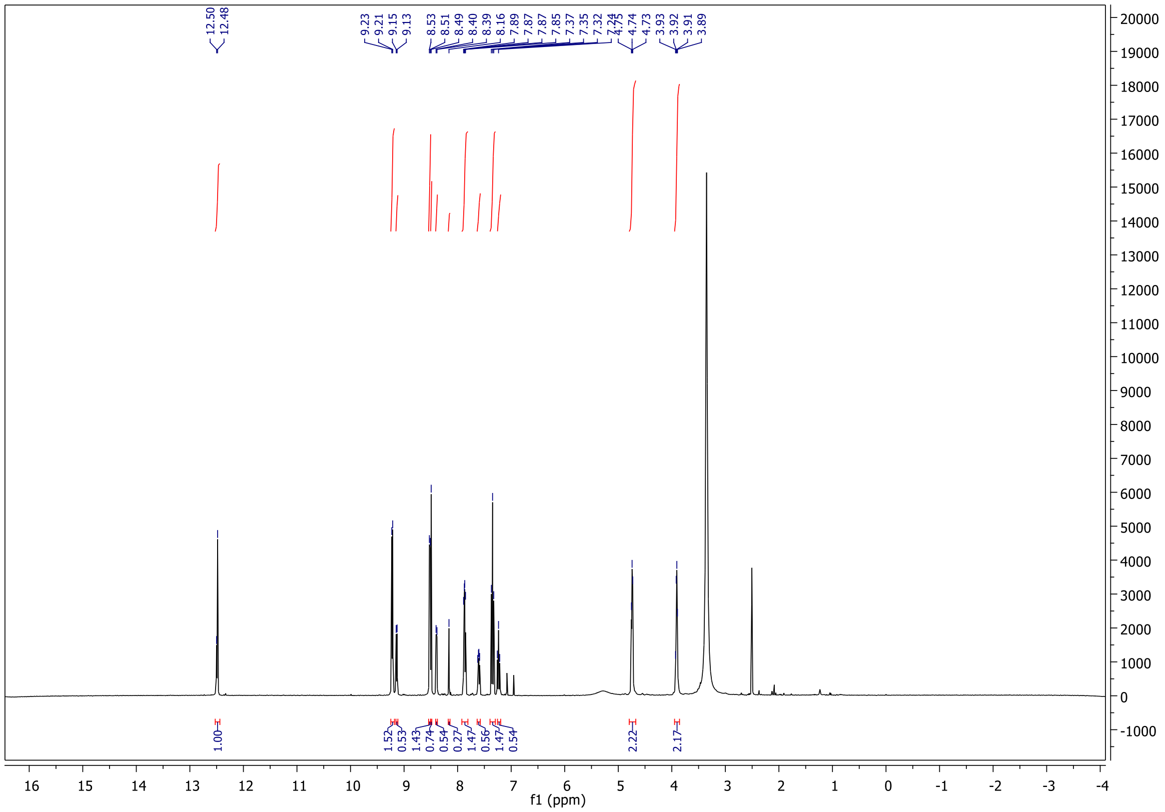

Figure S21. ^{1}HNMR of Compound 7

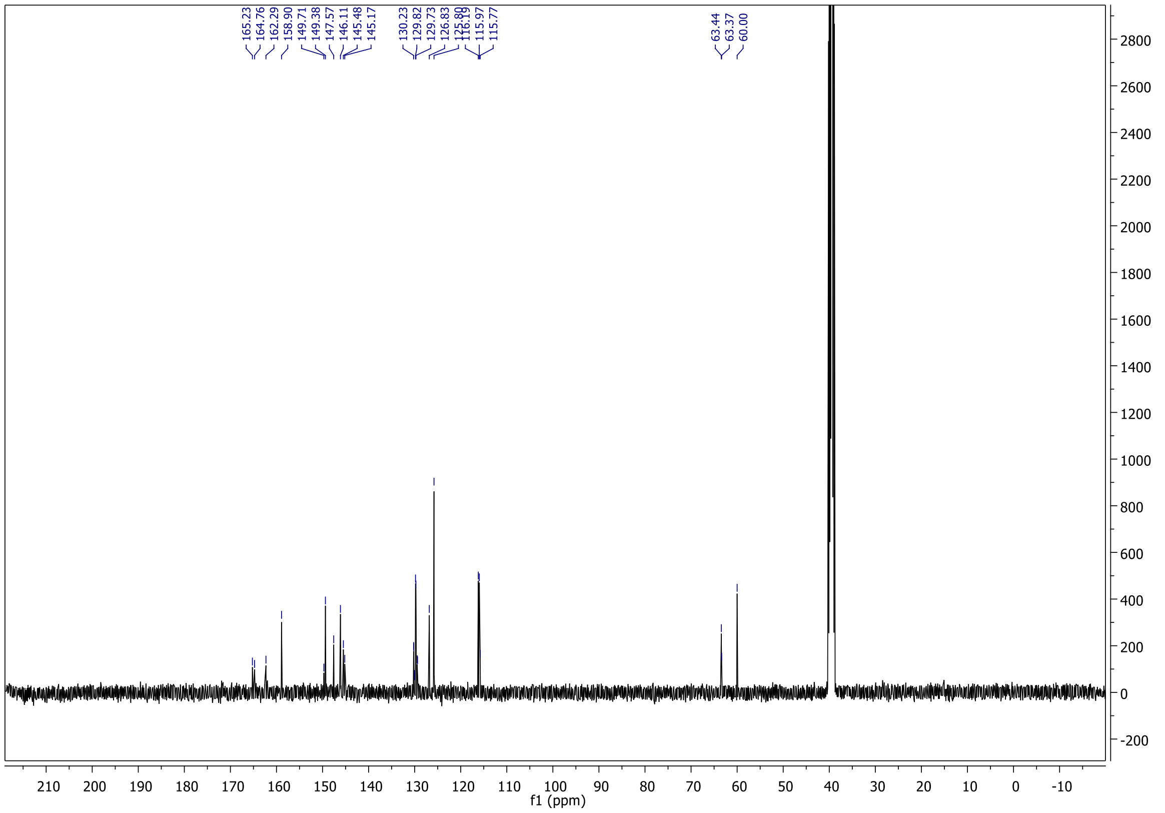

Figure S22. ^{13}CNMR of Compound 7

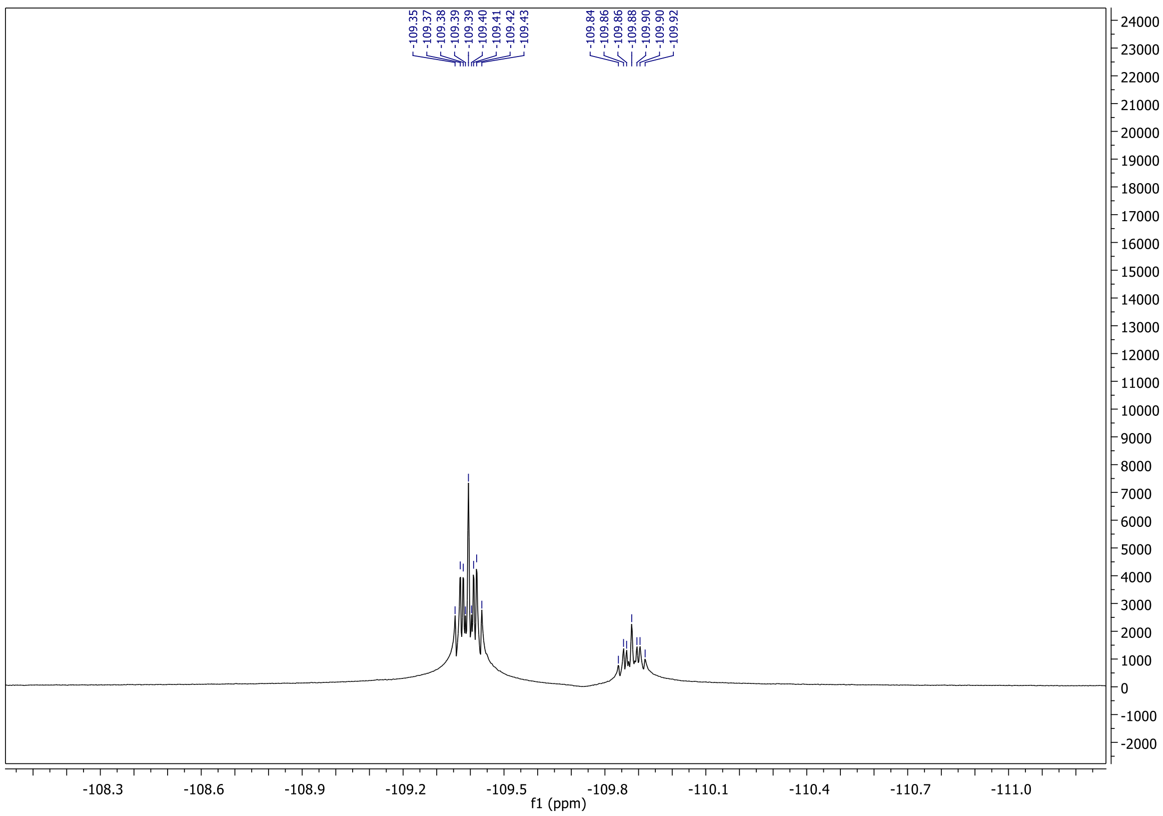

Figure S23. ^{19}FNMR of Compound 7

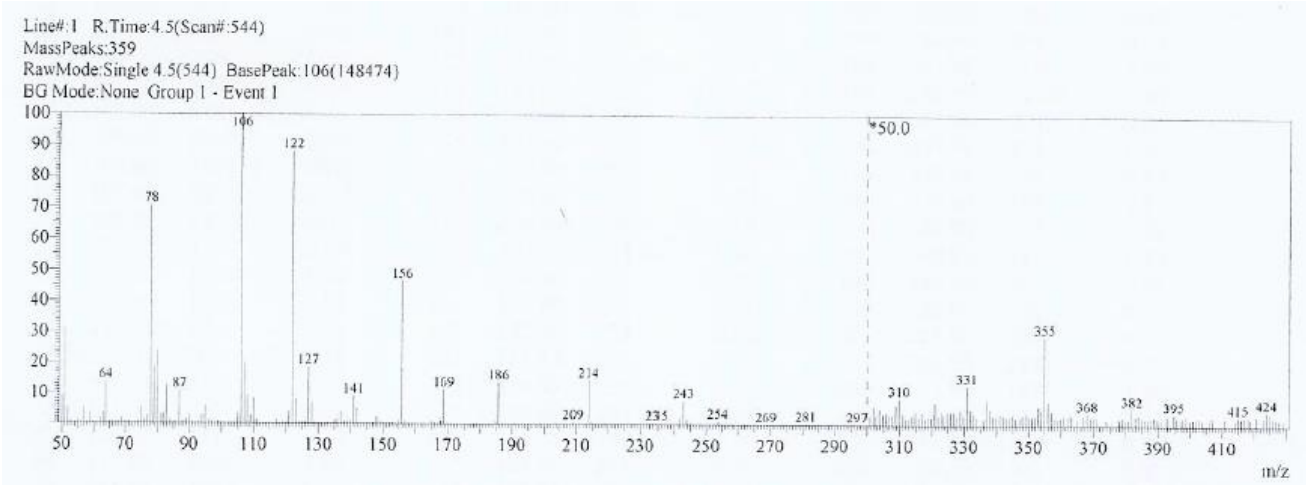

Figure S24. MS (ESI) of Compound 7

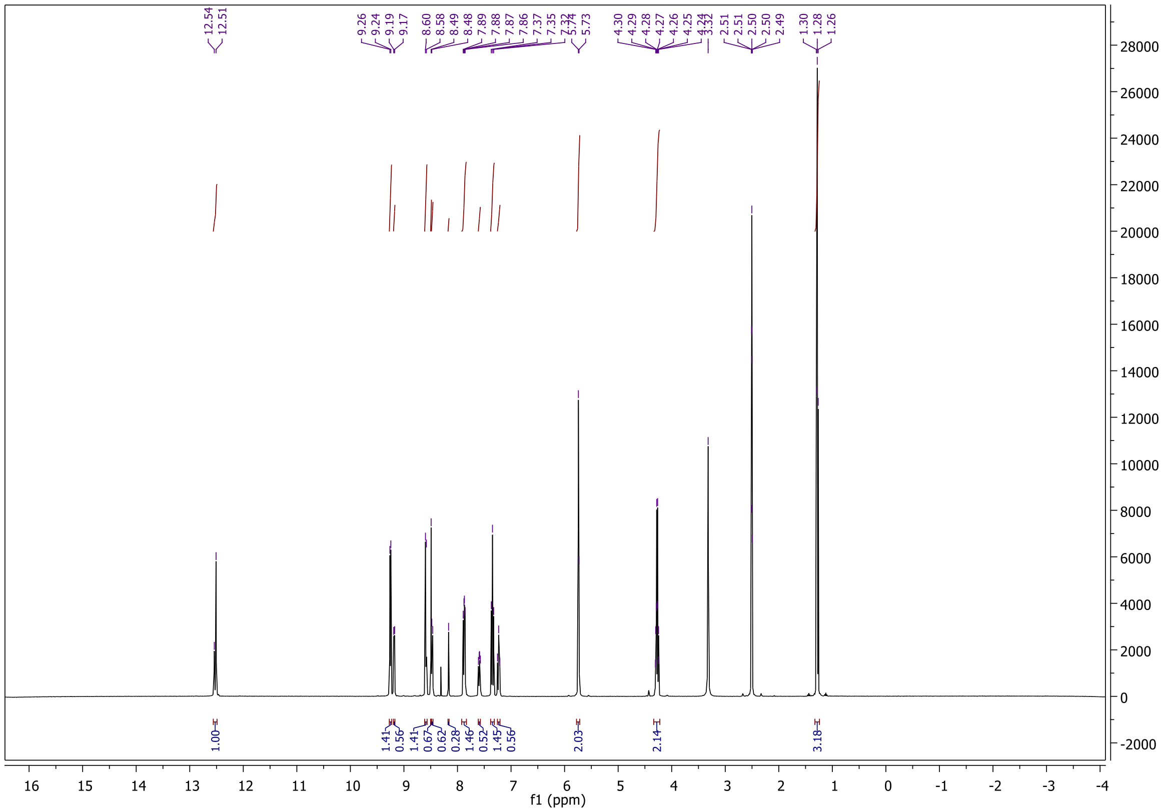

Figure S25. ^{1}HNMR of Compound 8

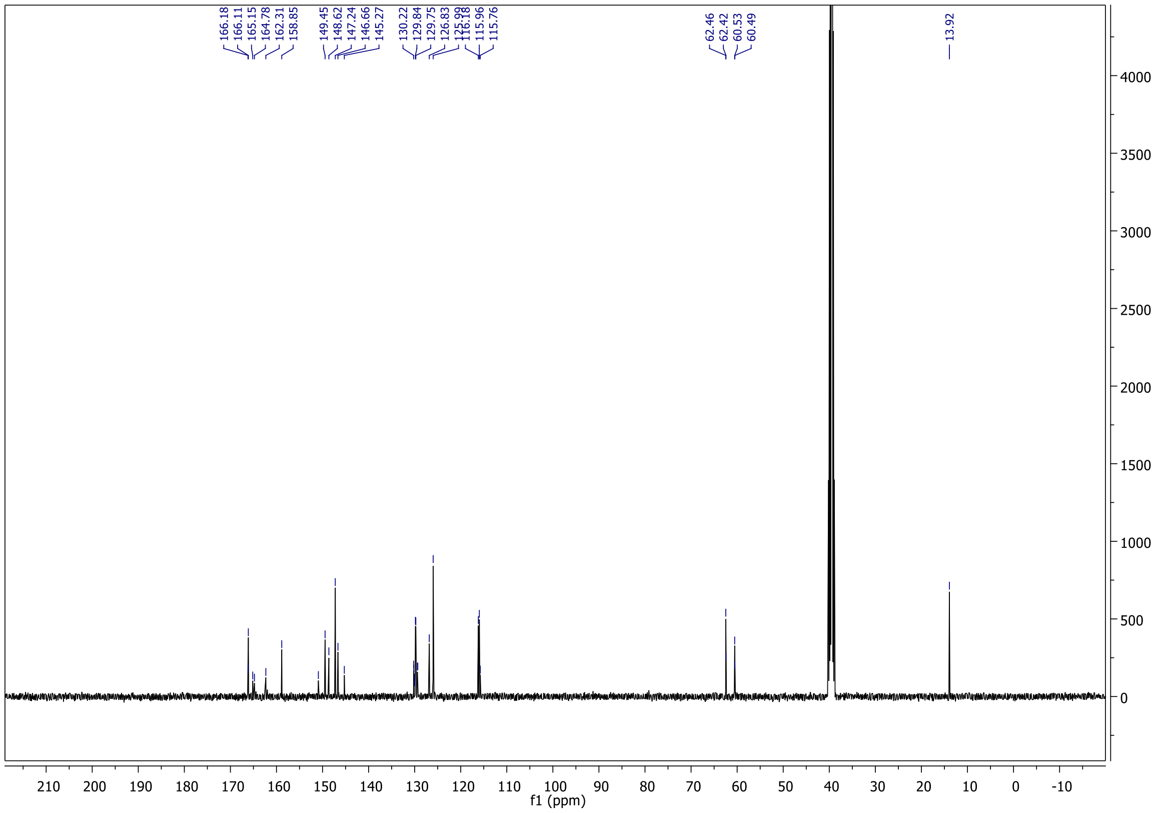

Figure S26. ^{13}CNMR of Compound 8

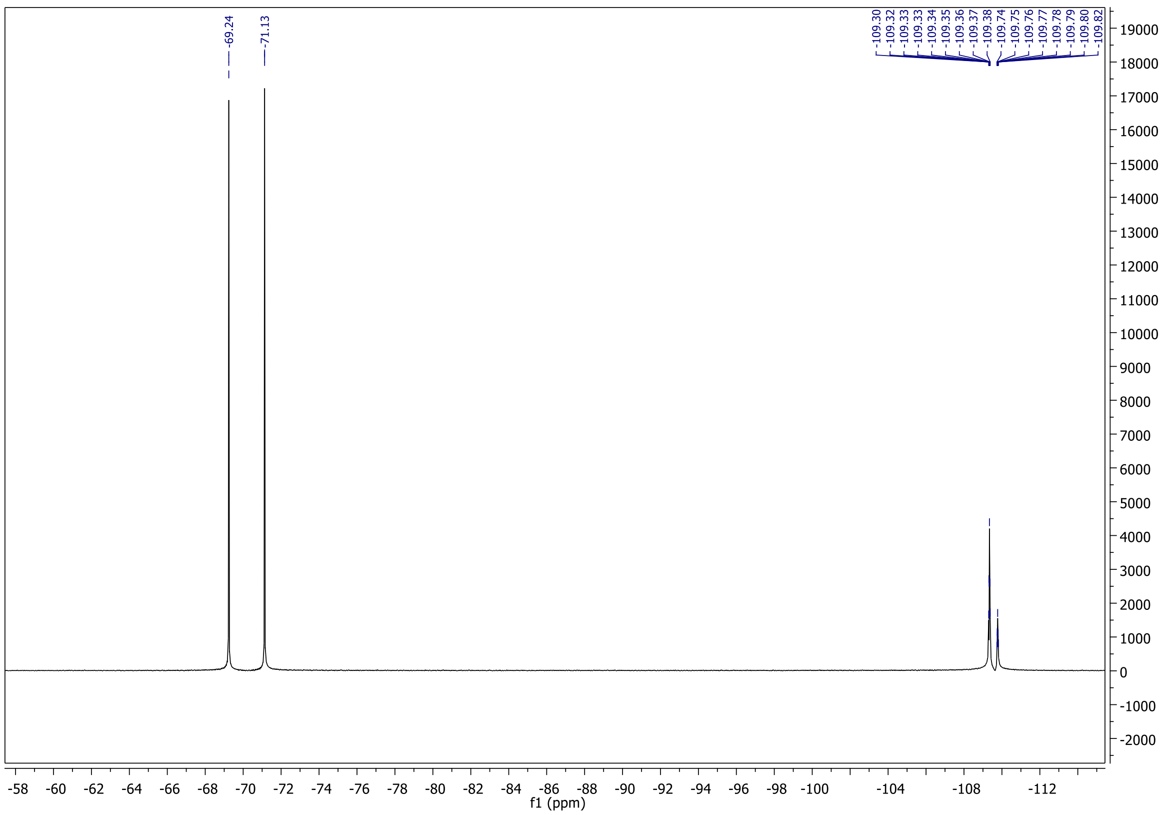

Figure S27. ^{19}FNMR of Compound 8

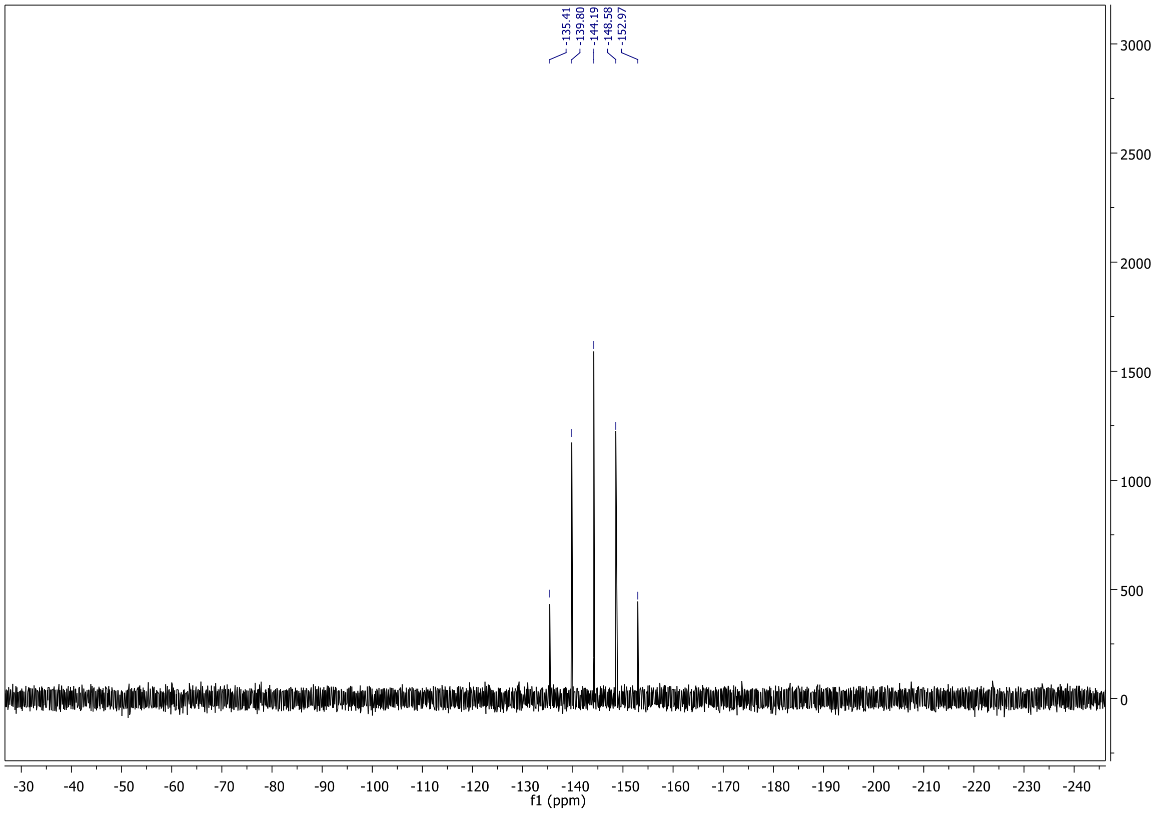

Figure S28. ^{31}PNMR of Compound 8

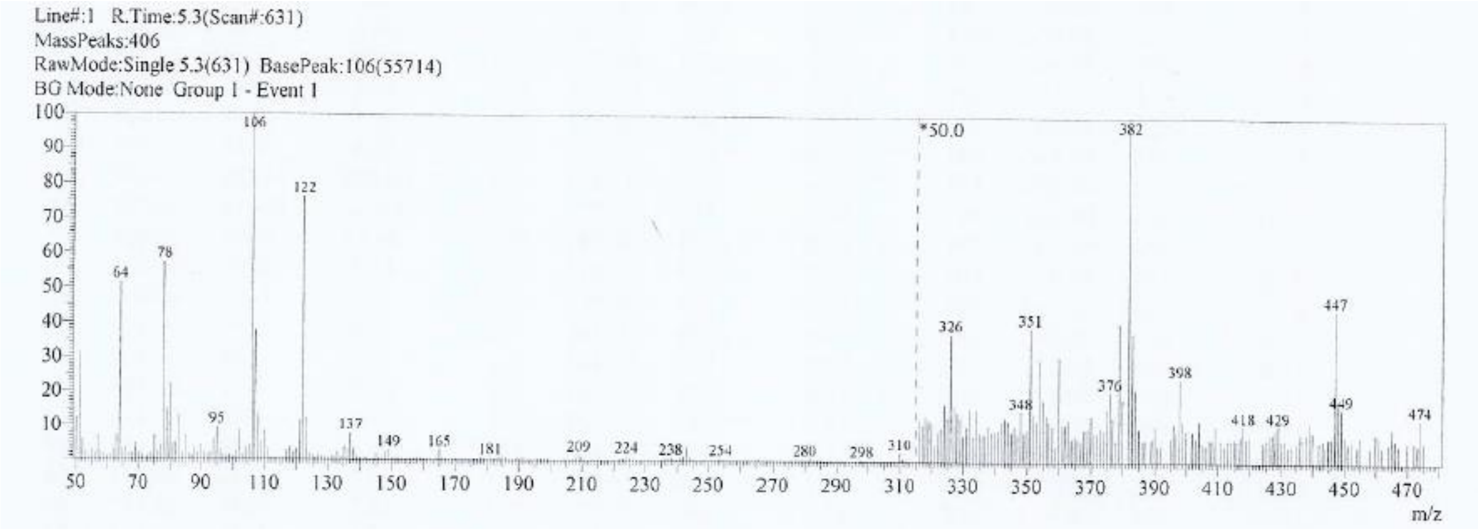

Figure S29. MS (ESI) of Compound 8

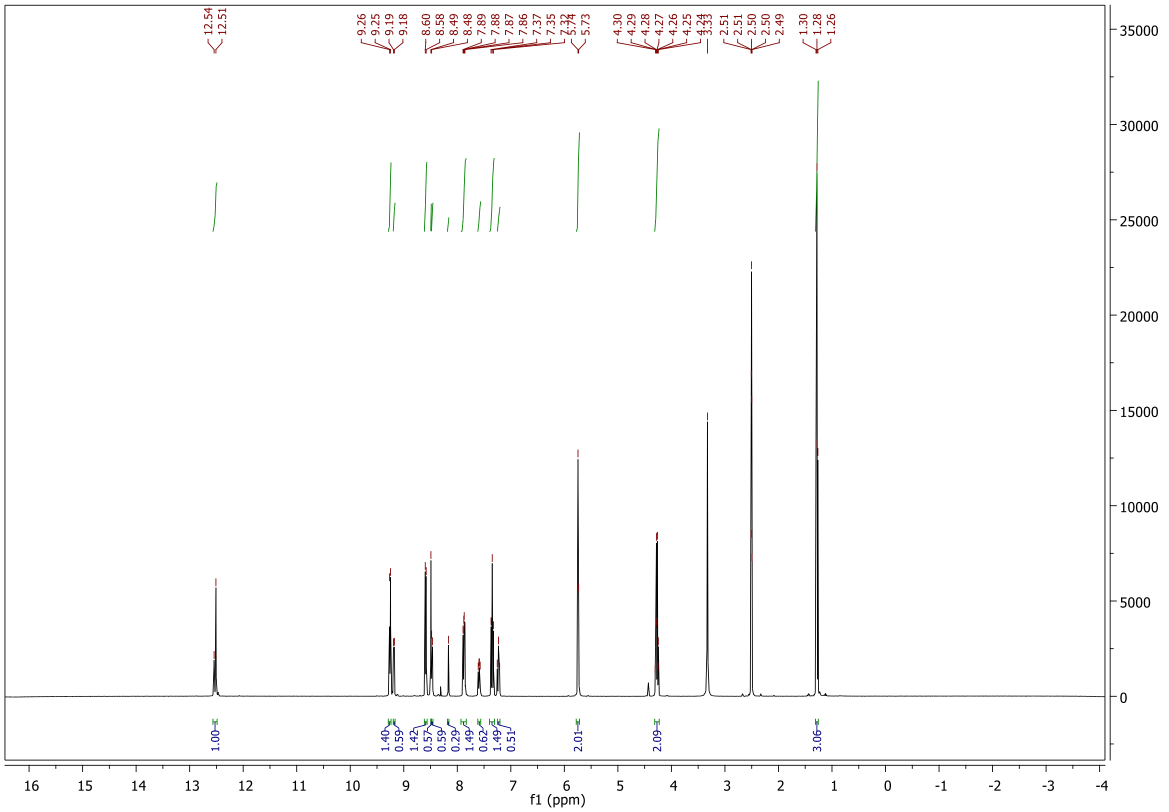

Figure S30. ^{1}HNMR of Compound 9

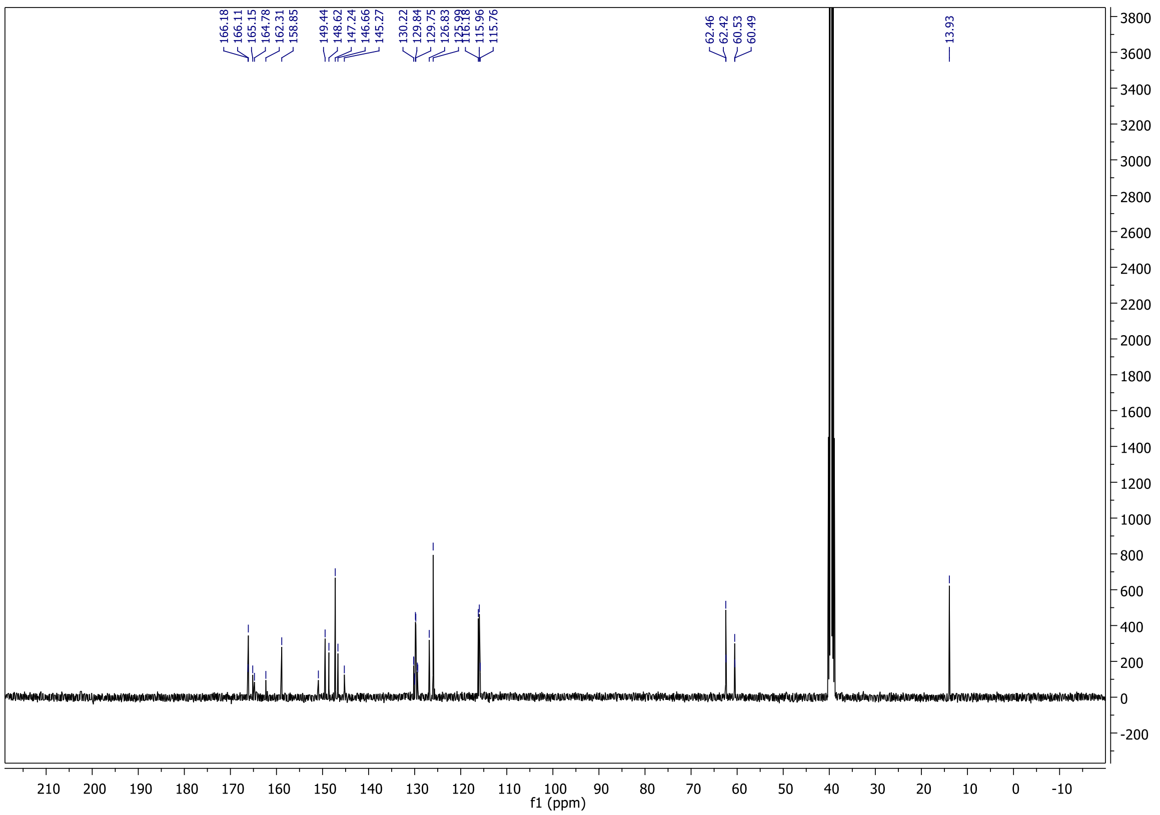

Figure S31. ^{13}CNMR of Compound 9

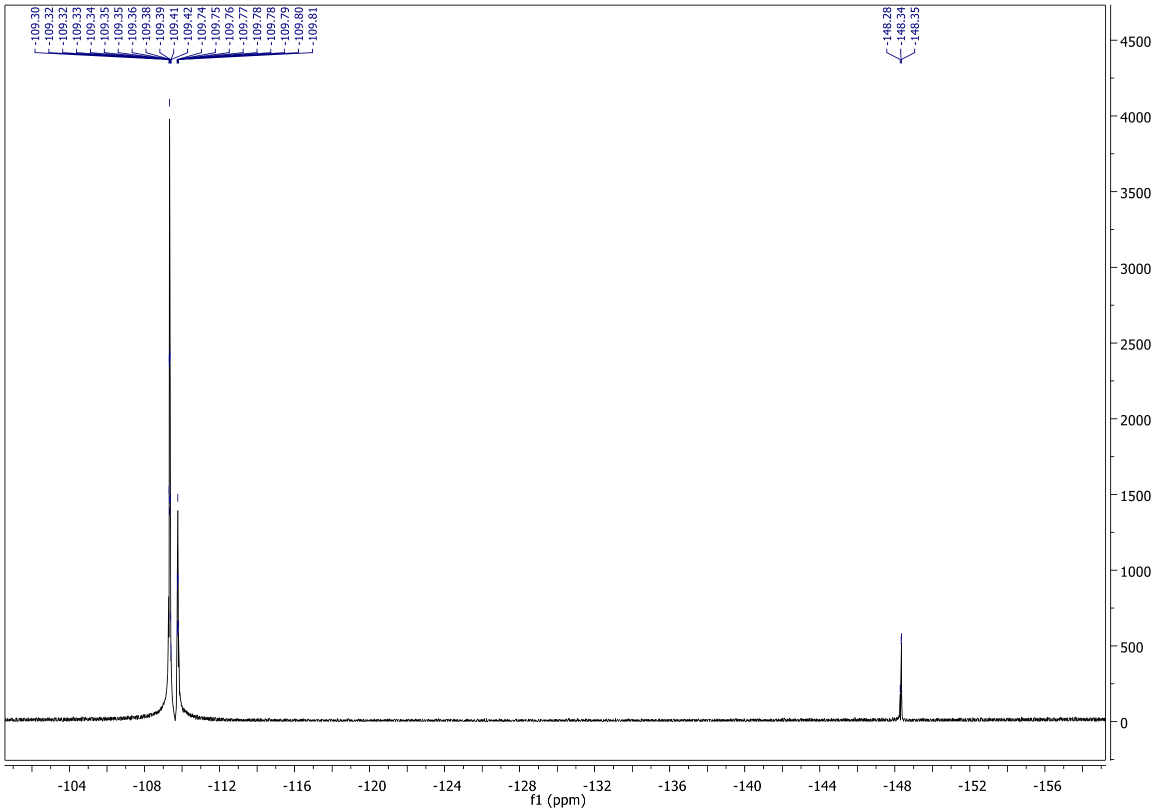

Figure S32. ^{19}FNMR of Compound 9

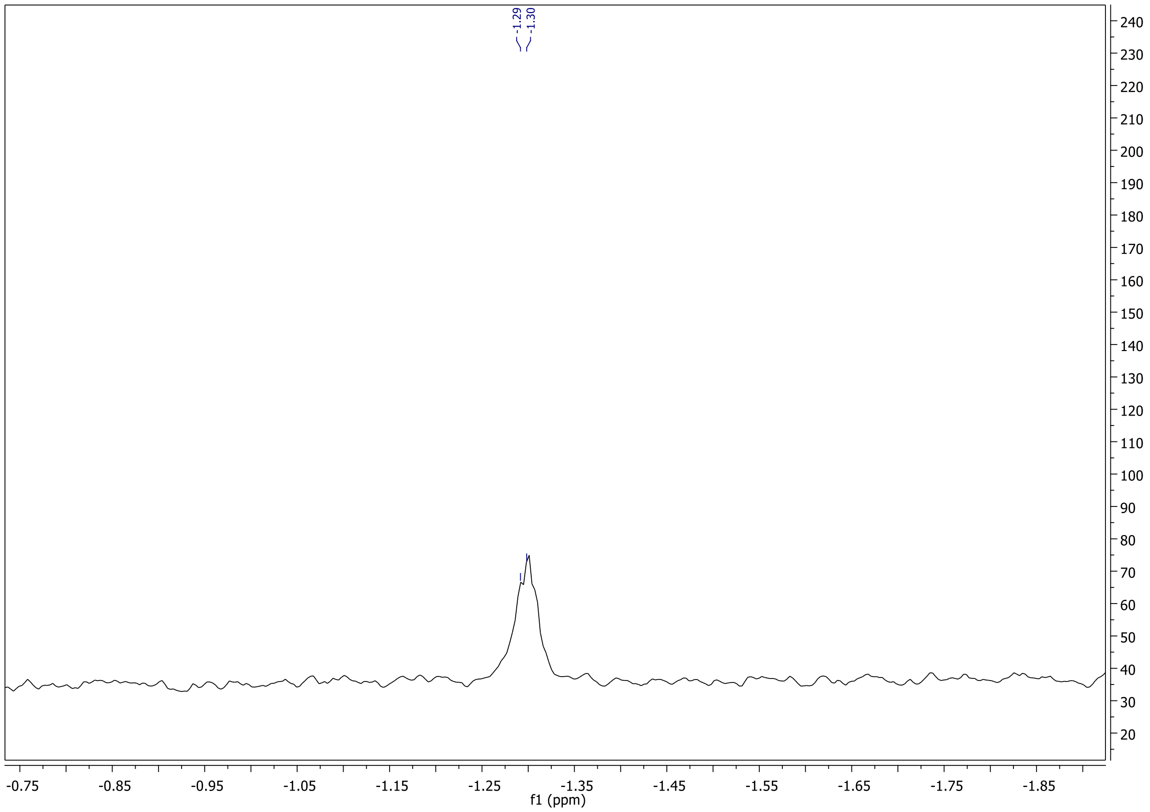

Figure S33. ^{11}BNMR of Compound 9

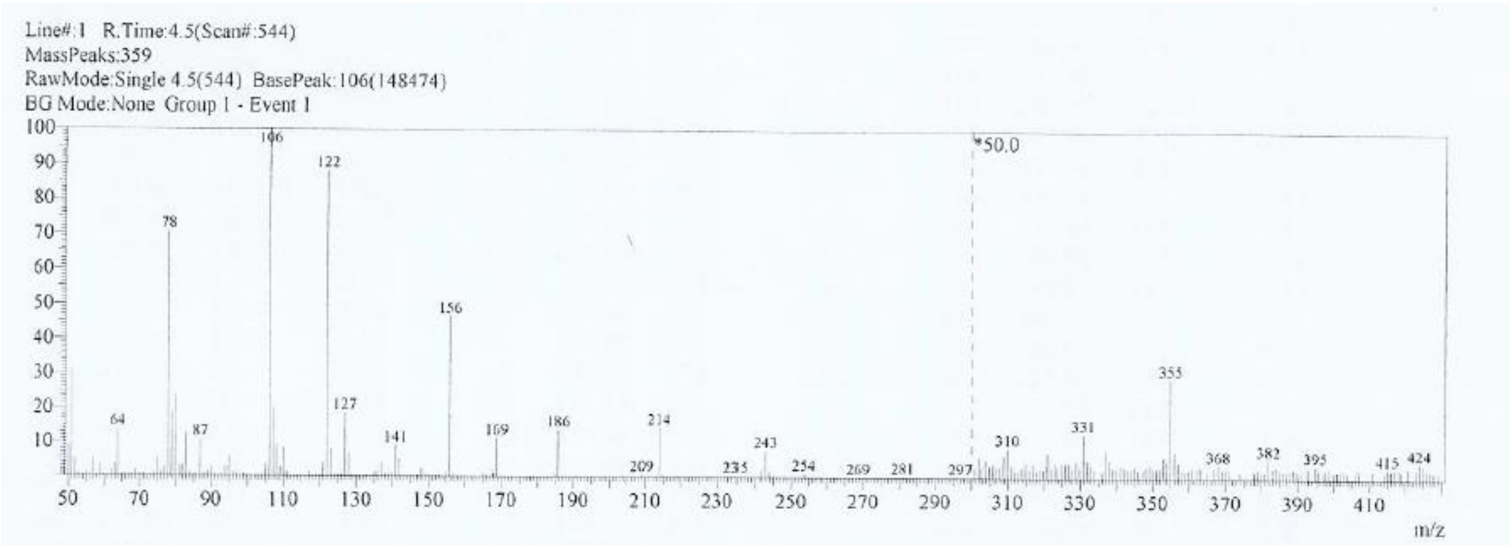

Figure S34. MS (ESI) of Compound 9

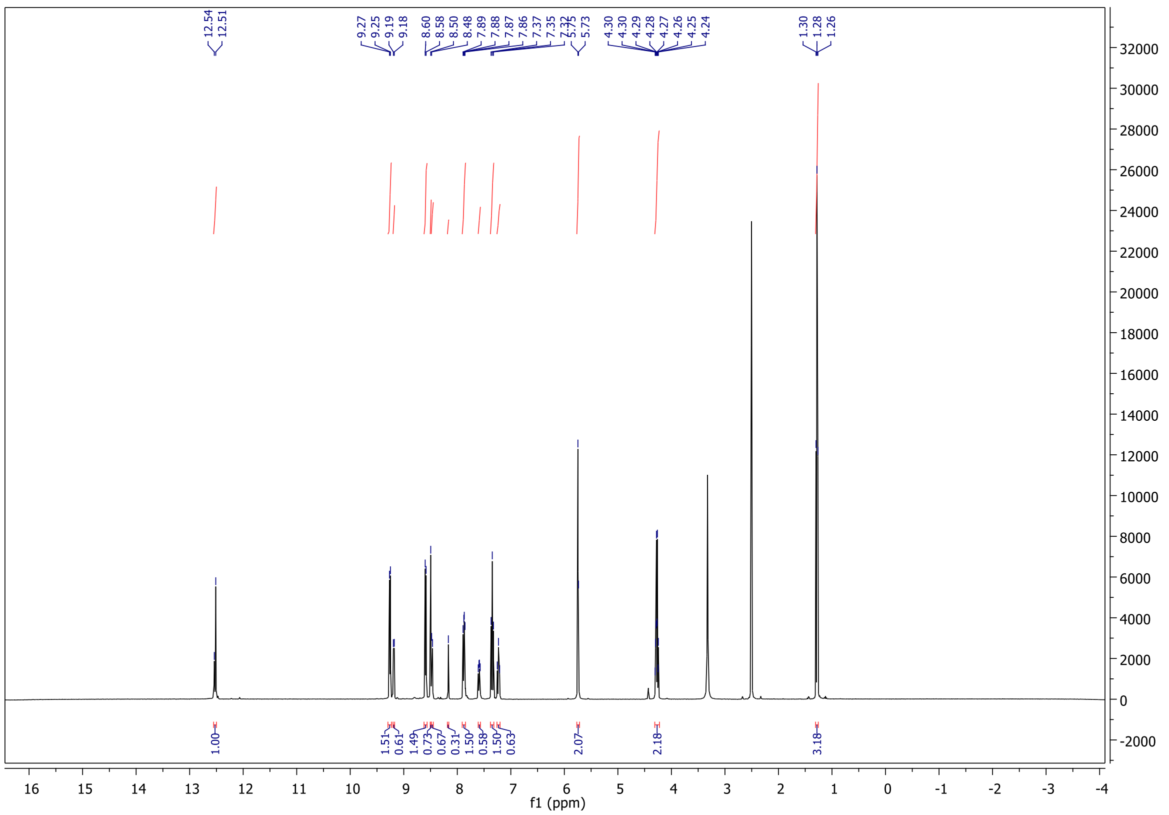

Figure S35. ^{1}HNMR of Compound 10

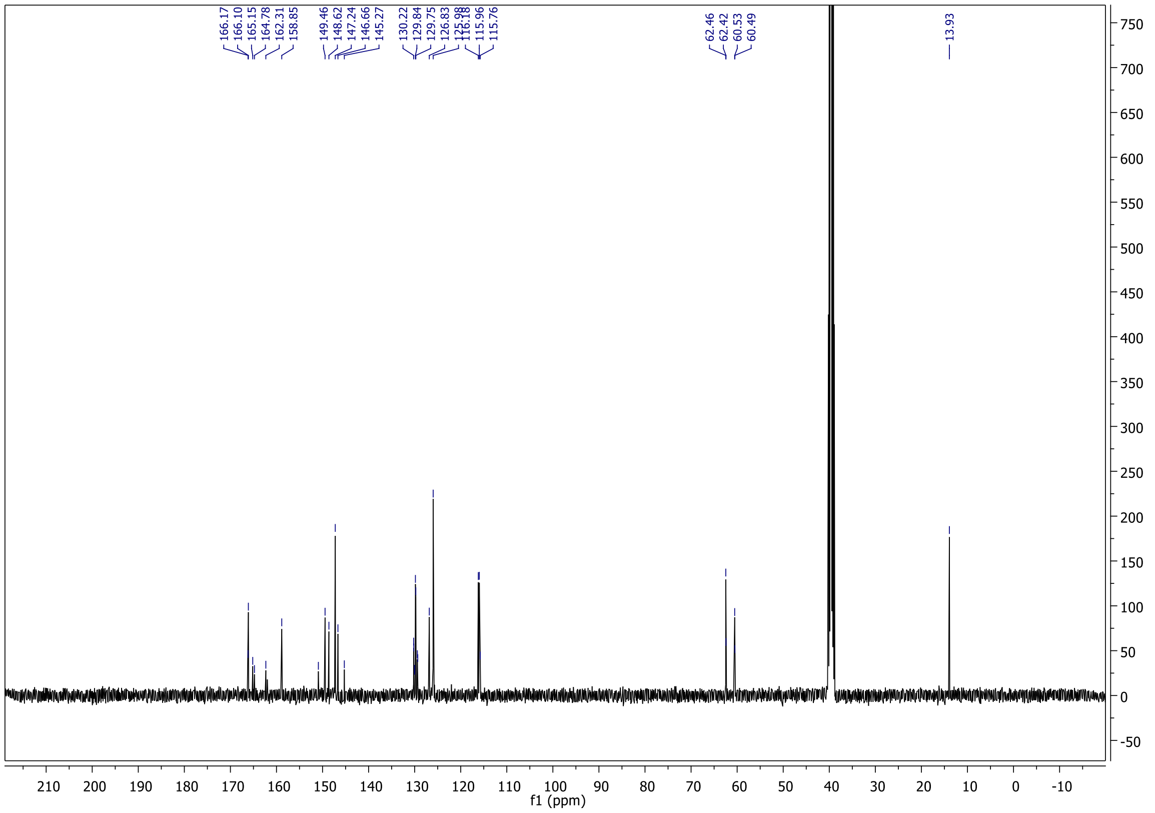

Figure S36. ^{13}CNMR of Compound 10

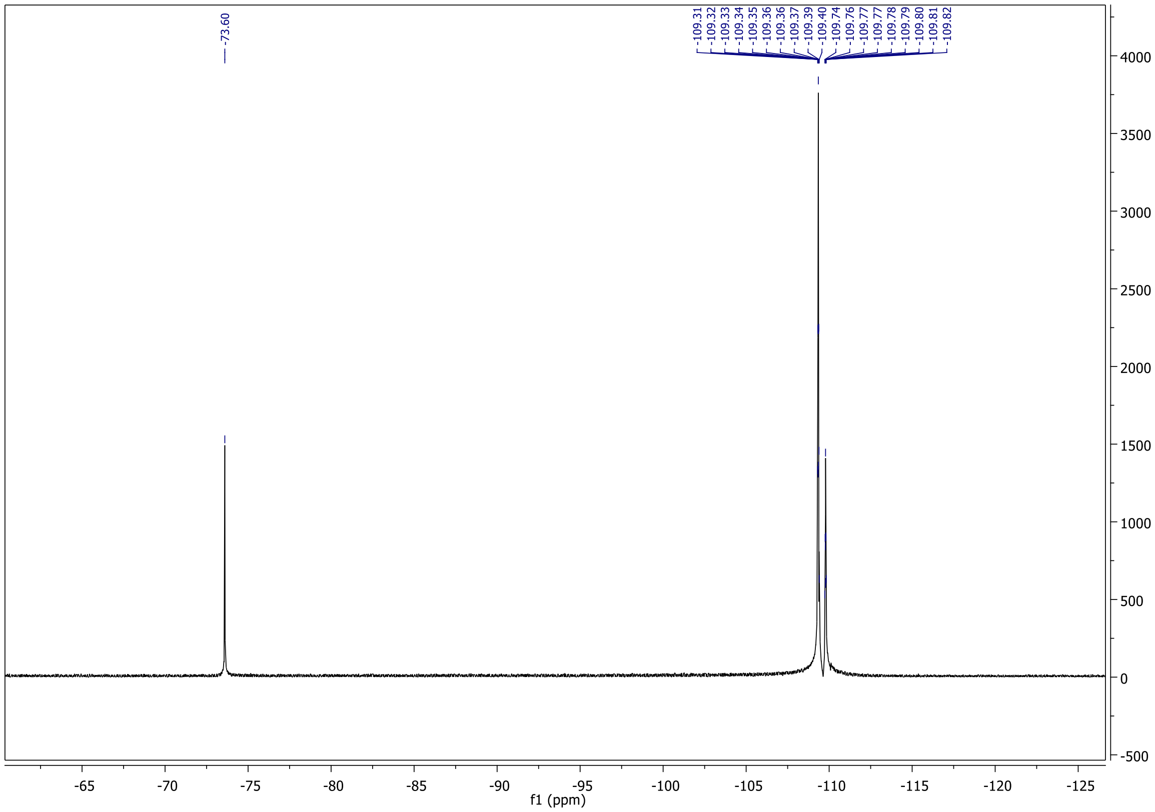

Figure S37. ^{19}FNMR of Compound 10

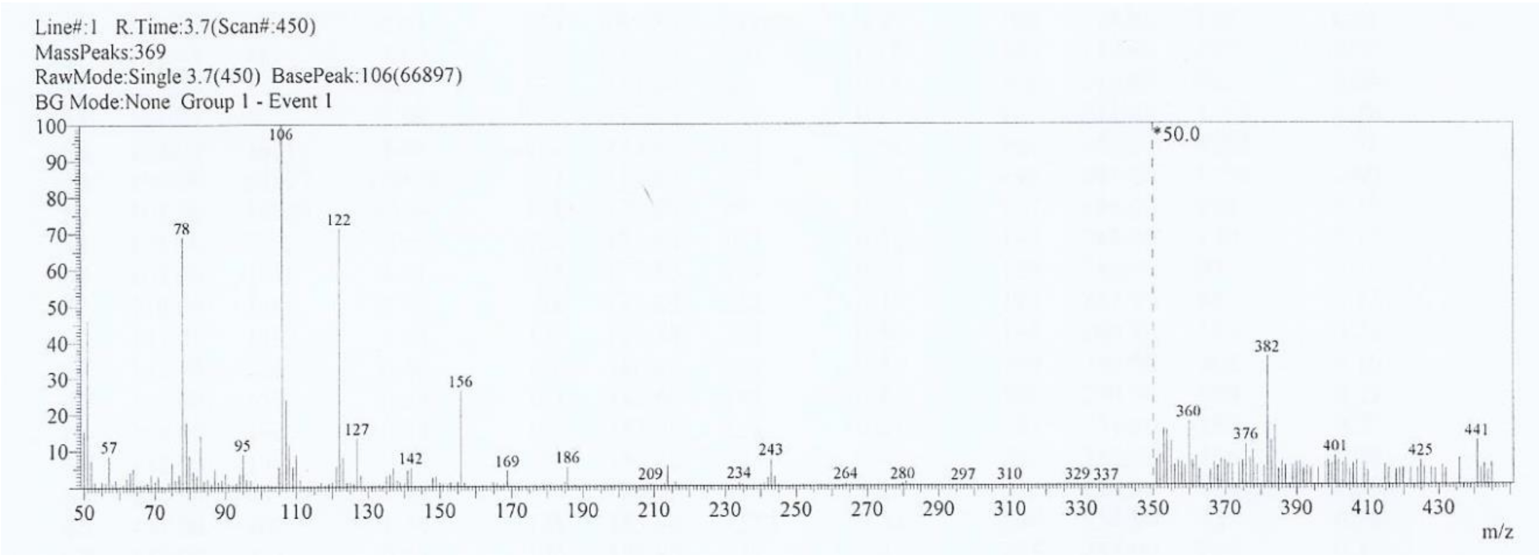

Figure S38. MS (ESI) of Compound 10

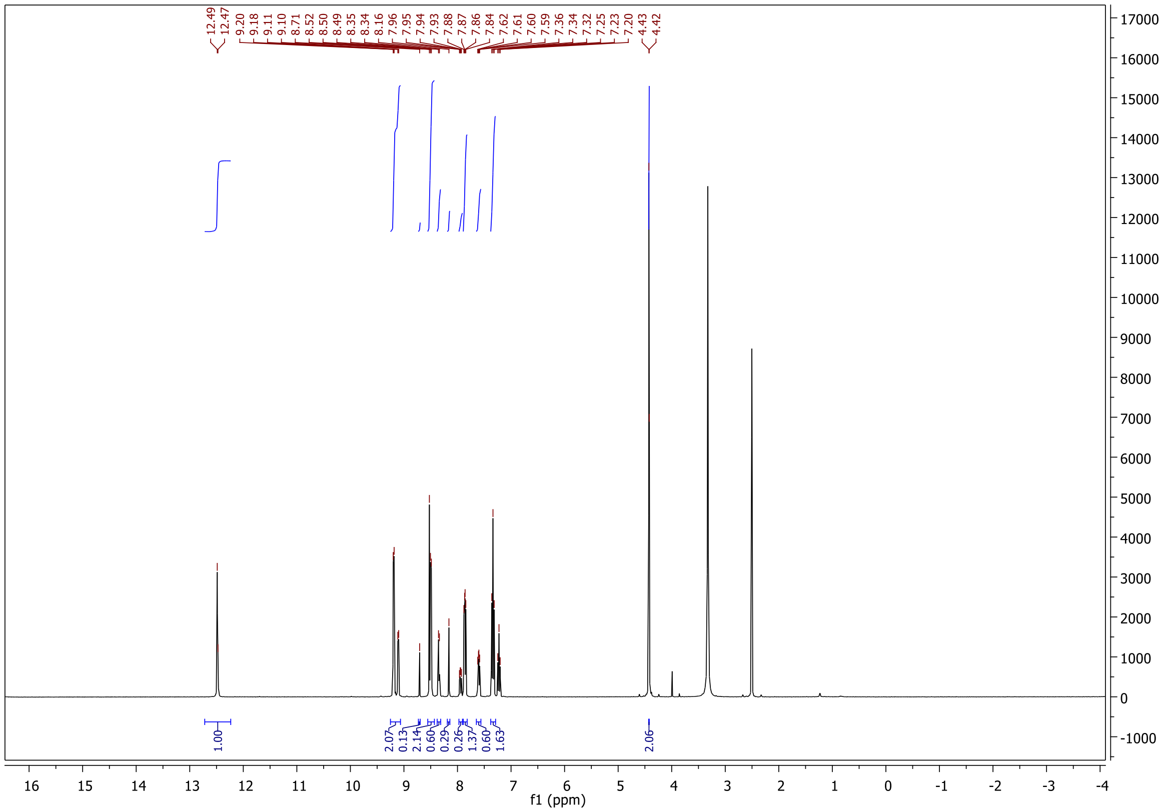

Figure S39. ^{1}HNMR of Compound 11

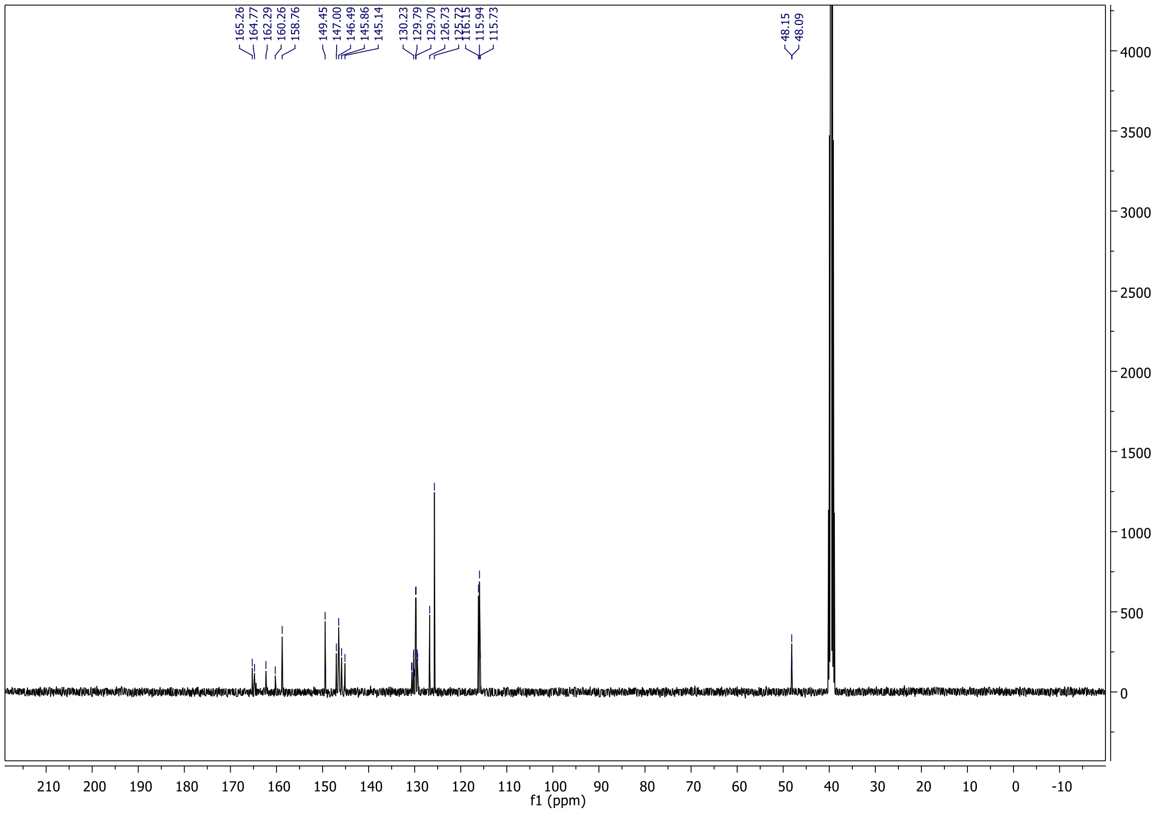

Figure S40. ^{13}CNMR of Compound 11

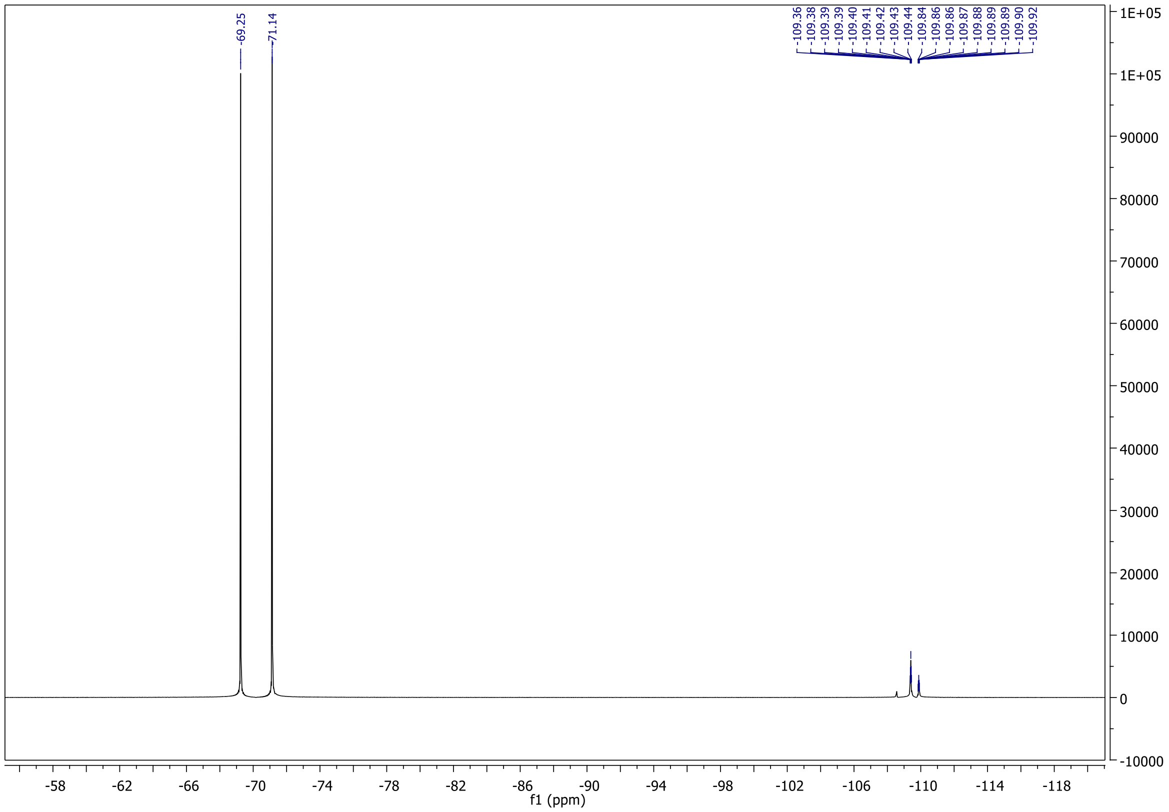

Figure S41. ^{19}FNMR of Compound 11

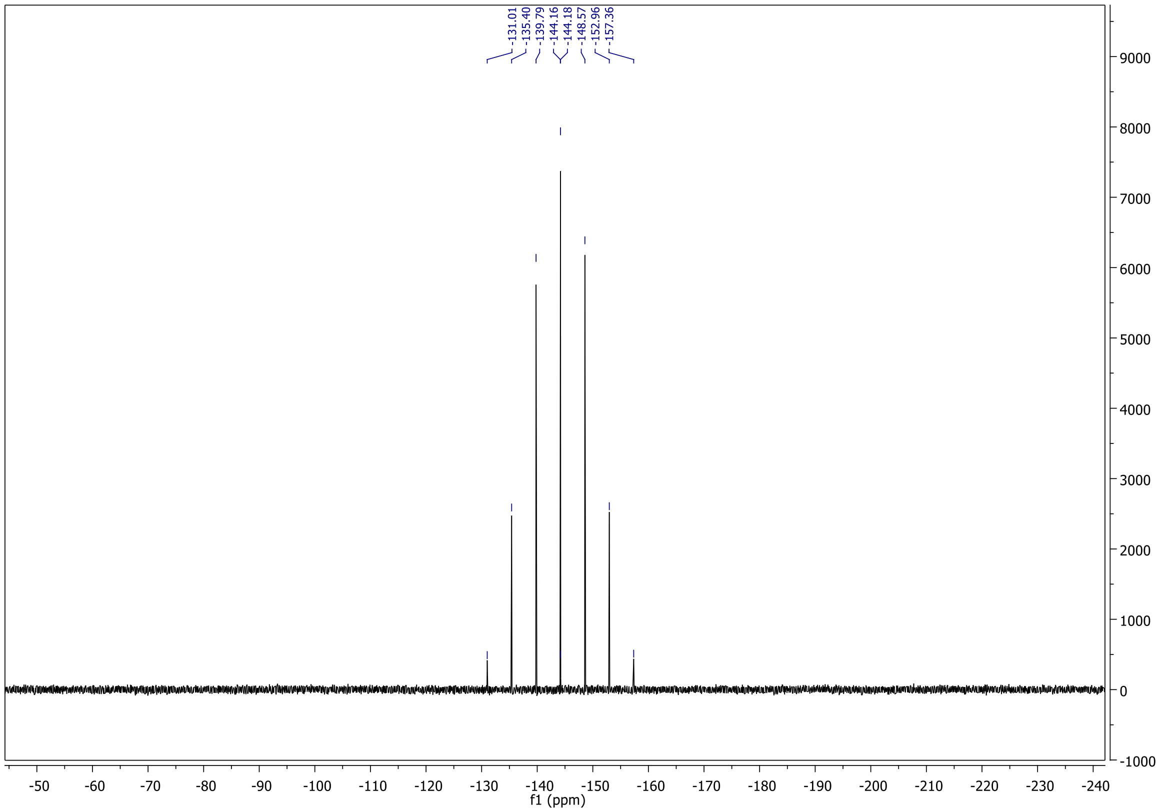

Figure S42. ^{31}PNMR of Compound 11

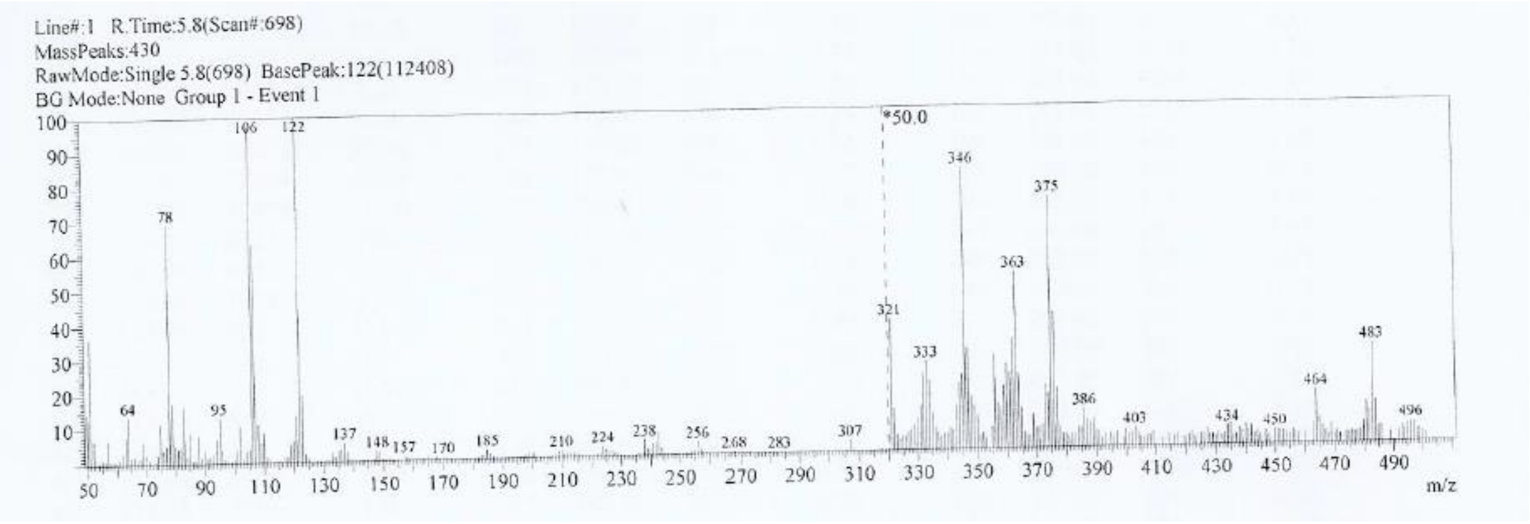

Figure S43. MS (ESI) of Compound 11

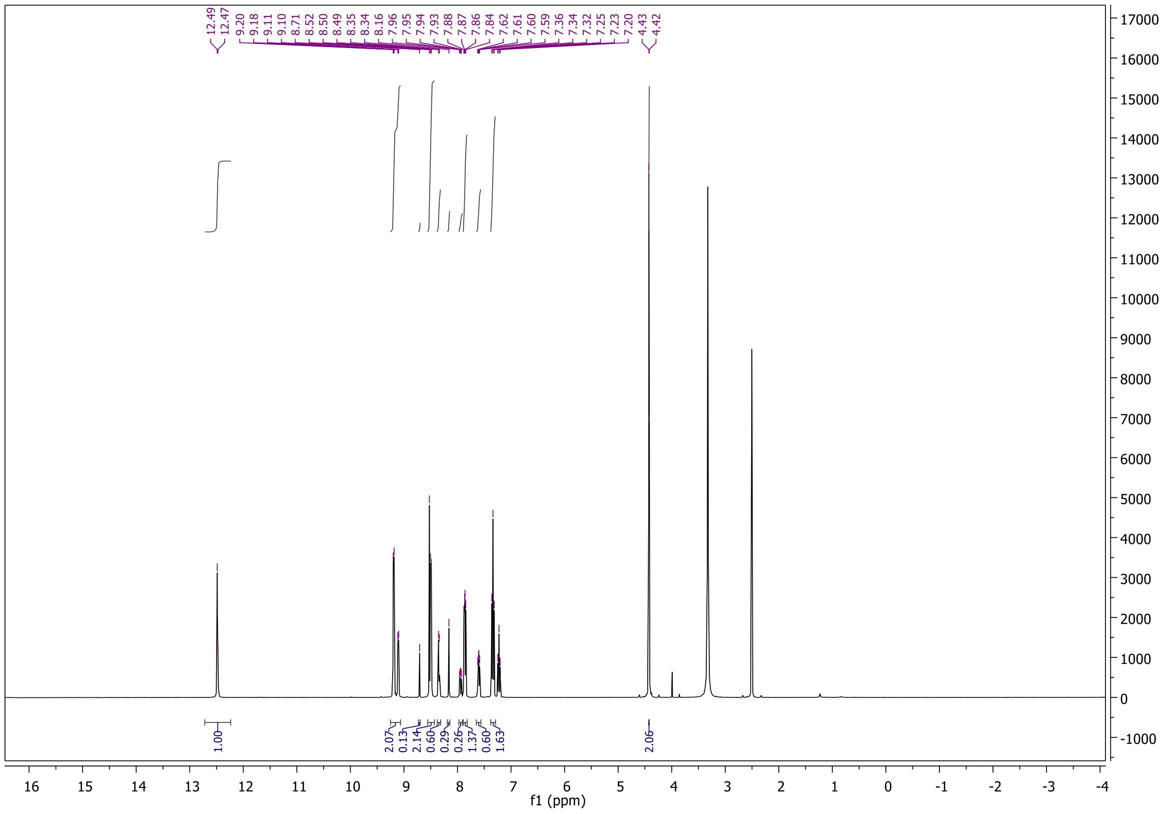

Figure S44. ^{1}HNMR of Compound 12

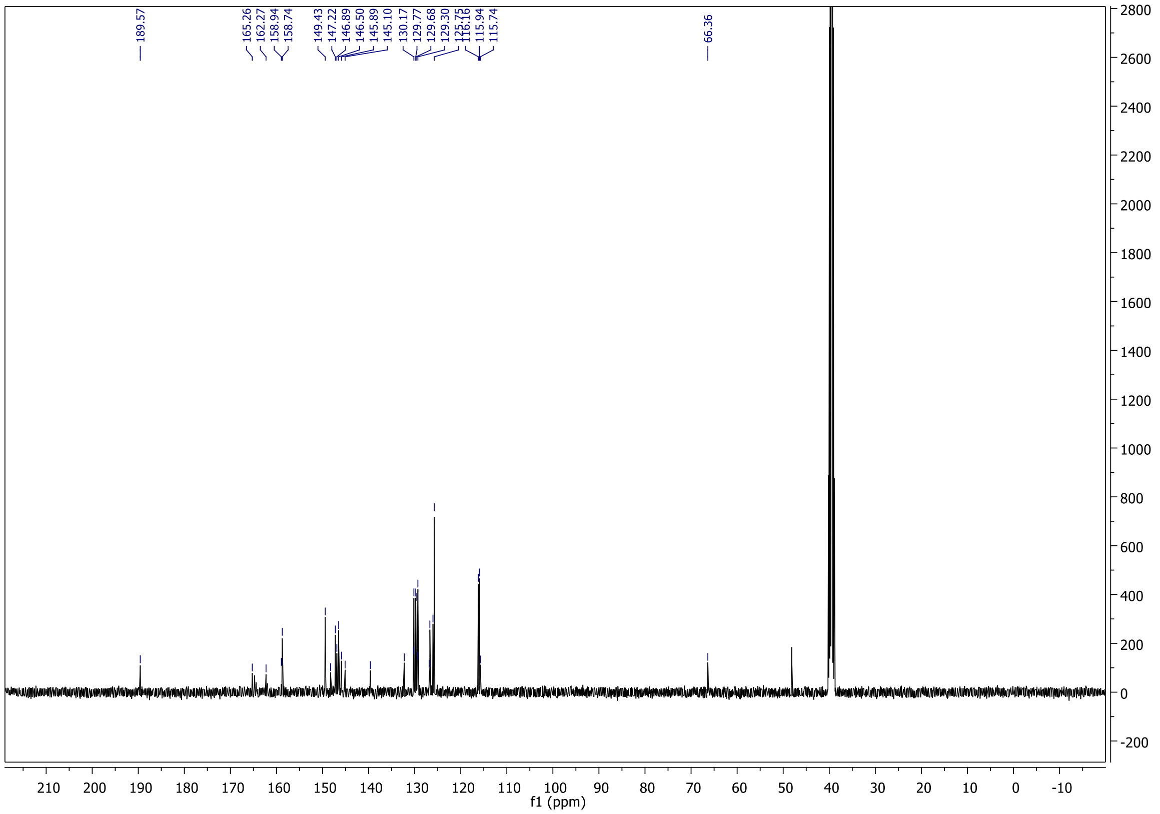

Figure S45. ^{13}CNMR of Compound 12

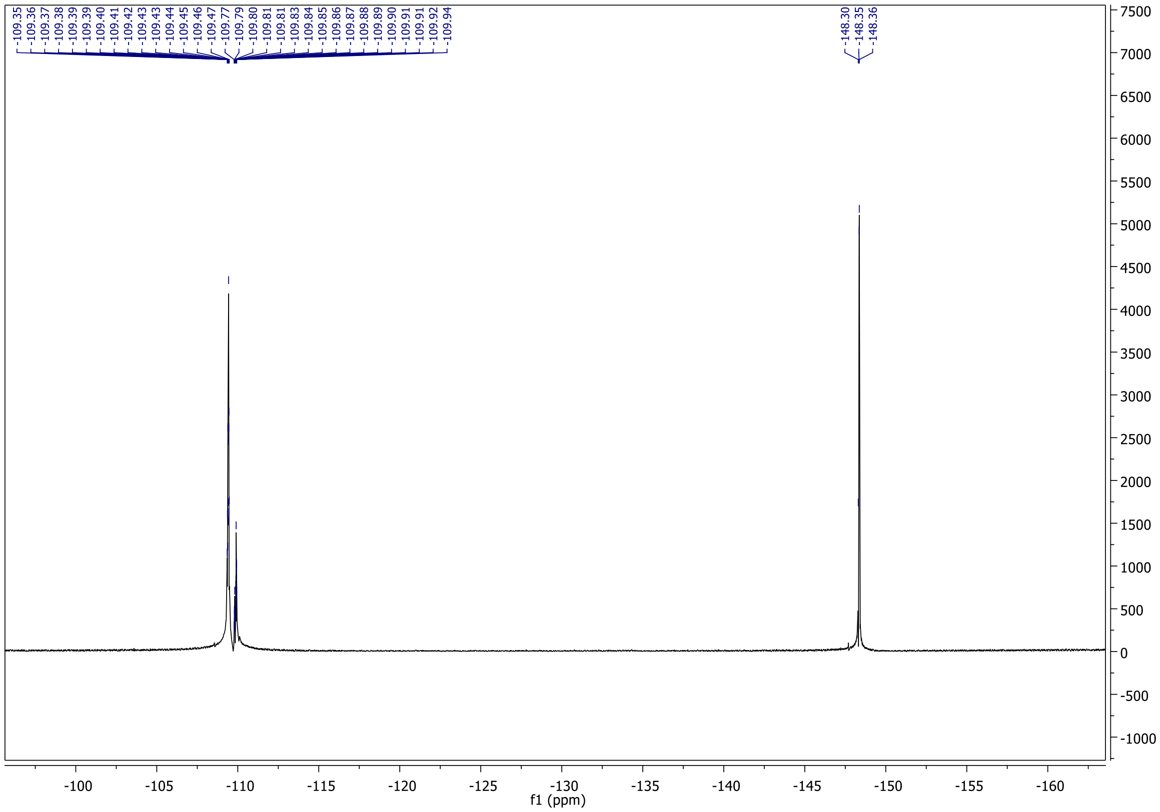

Figure S46. ^{19}FNMR of Compound 12

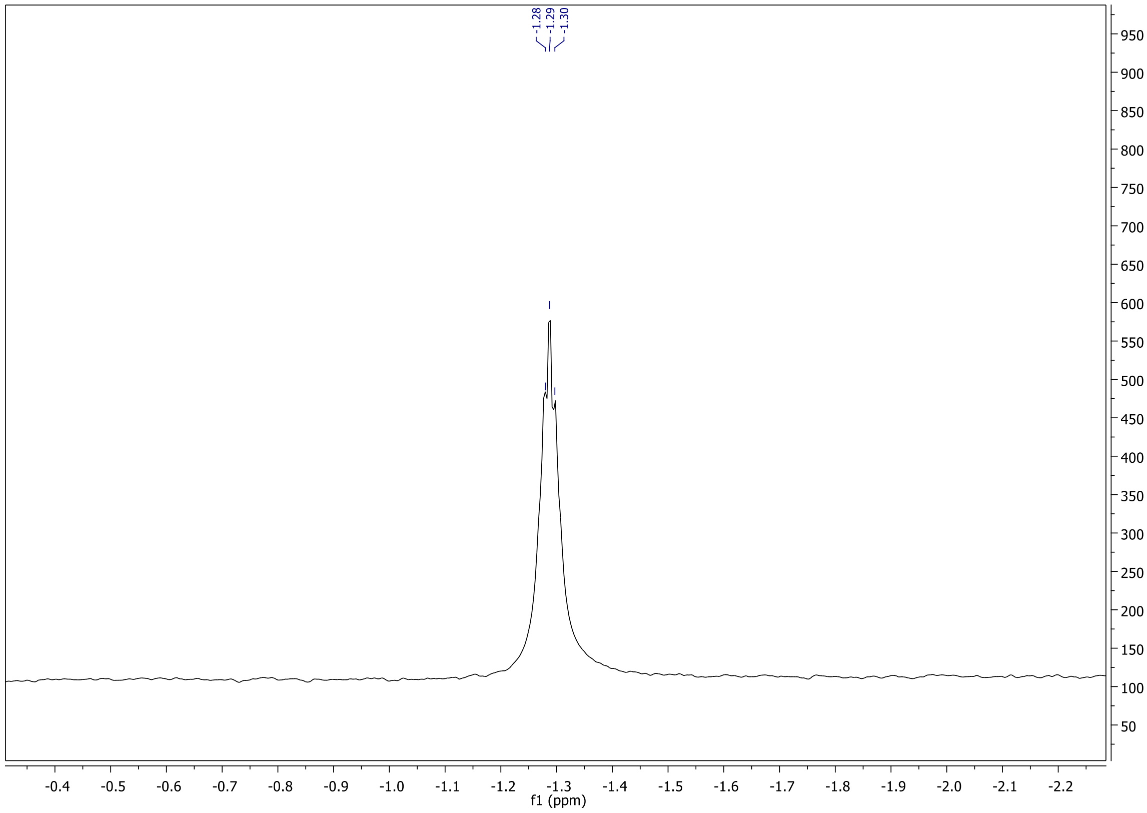

Figure S47. ^{11}BNMR of Compound 12

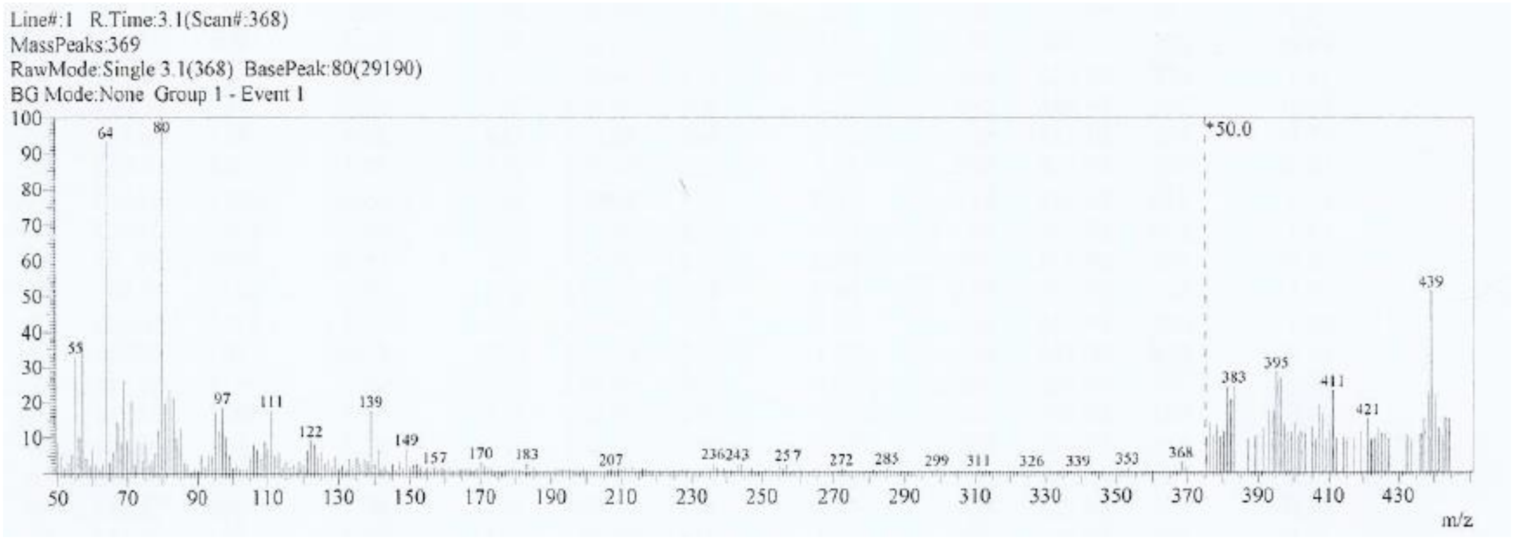

Figure S48. MS (ESI) of Compound 12

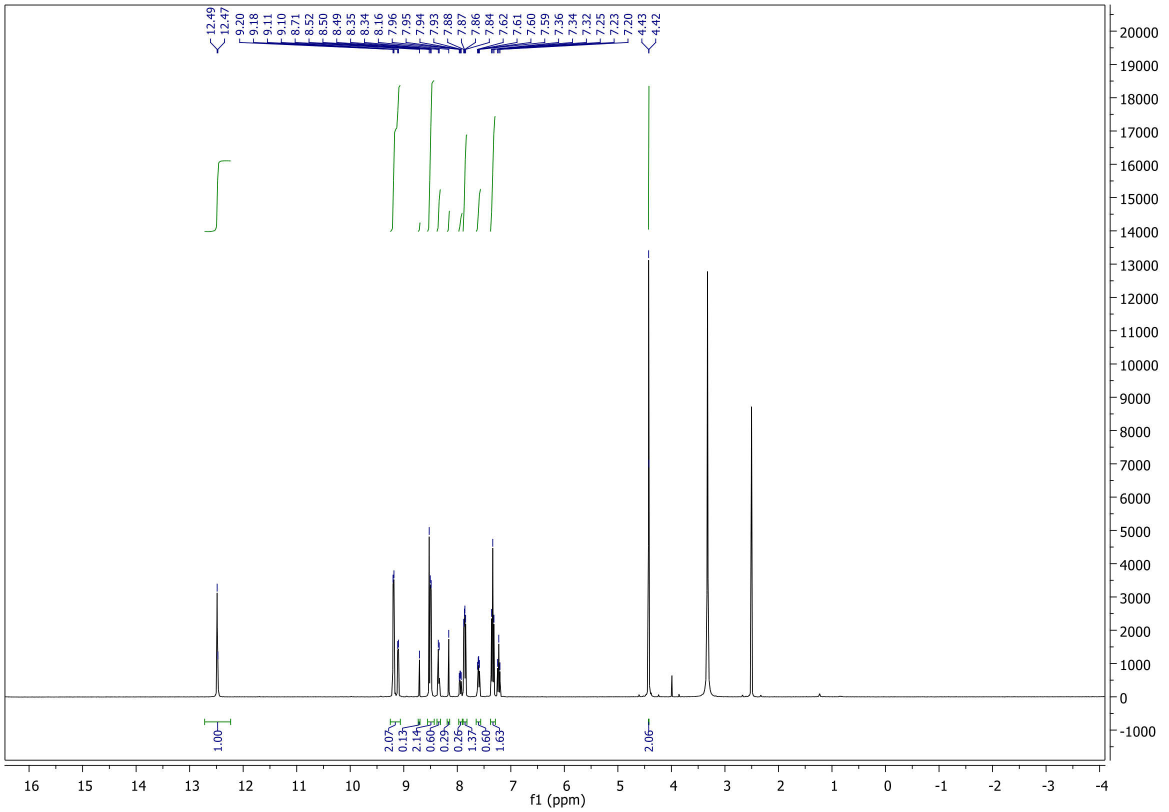

Figure S49. ^{1}HNMR of Compound 13

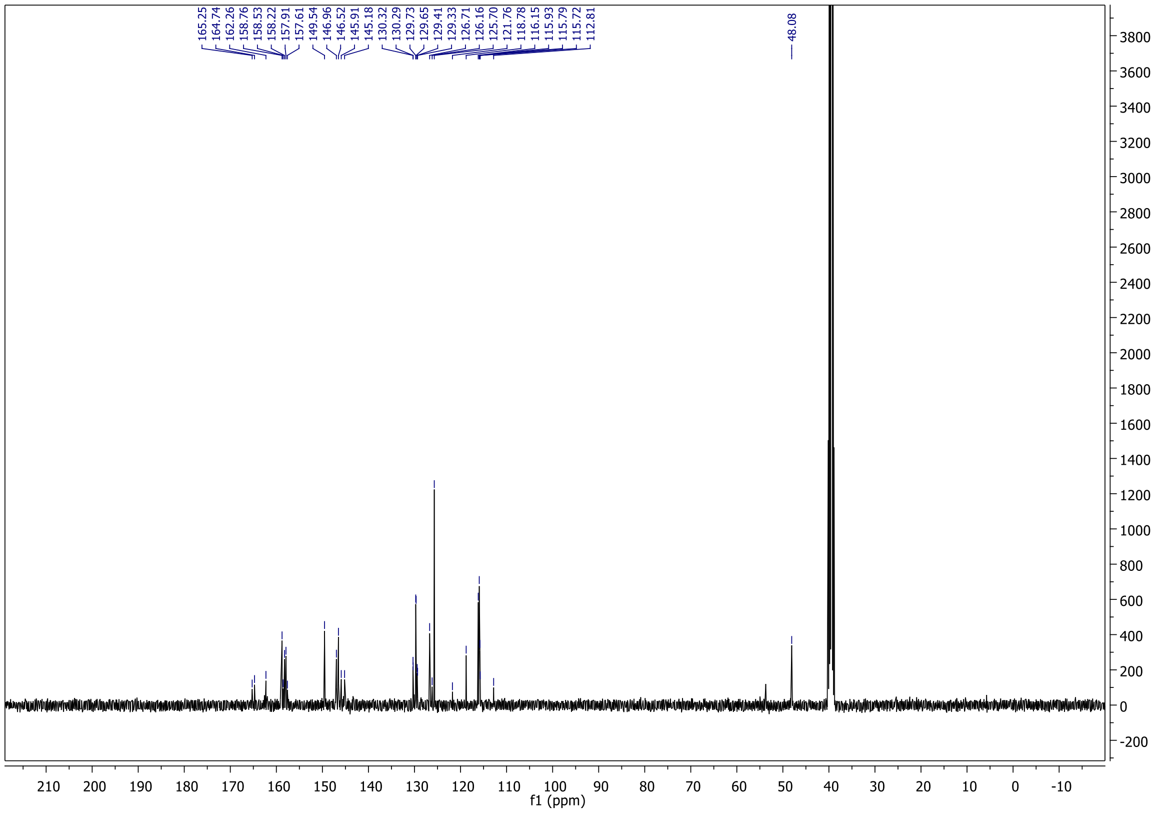

Figure S50. ^{13}CNMR of Compound 13

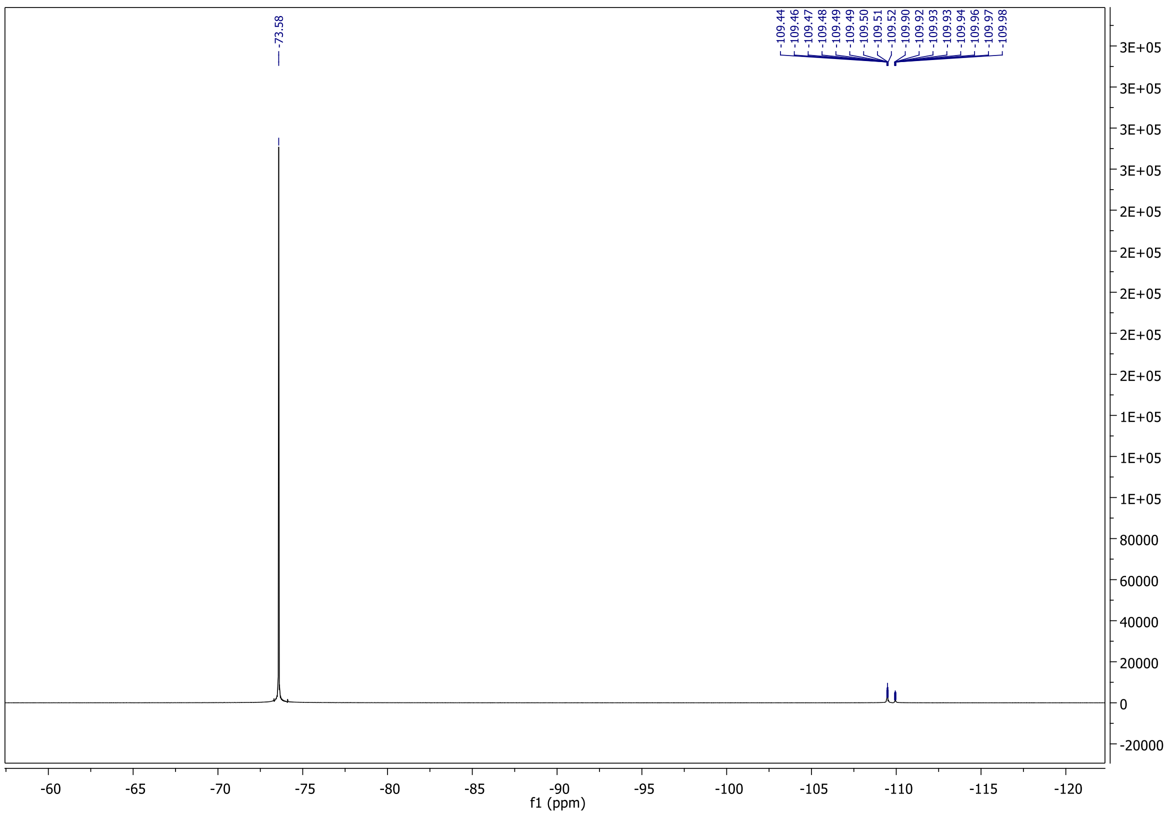

Figure S51. ^{19}FNMR of Compound 13

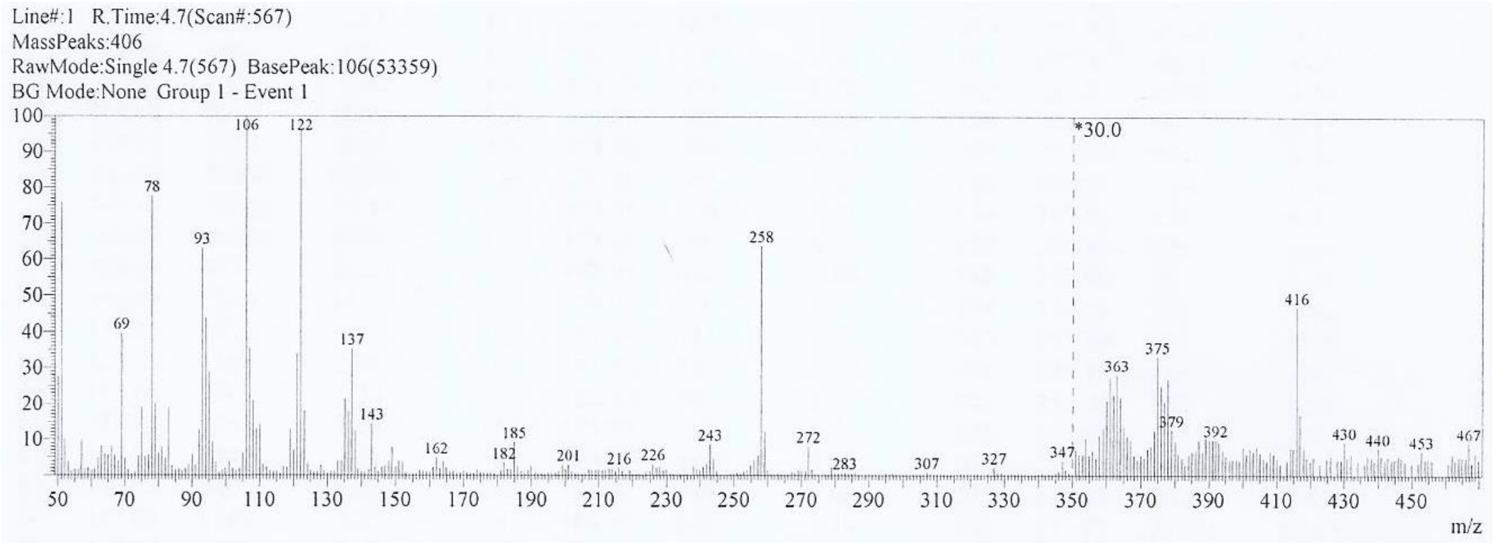

Figure S52. MS (ESI) of Compound 13

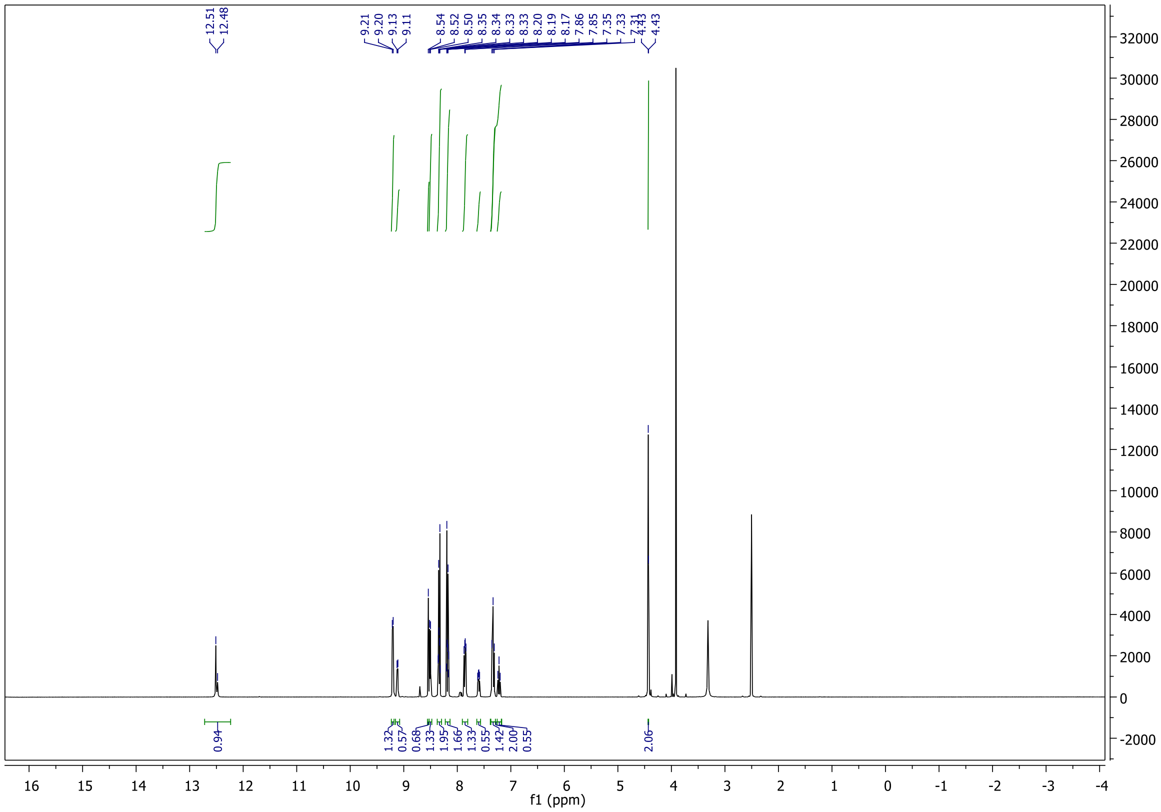

Figure S53. ^{1}HNMR of Compound 14

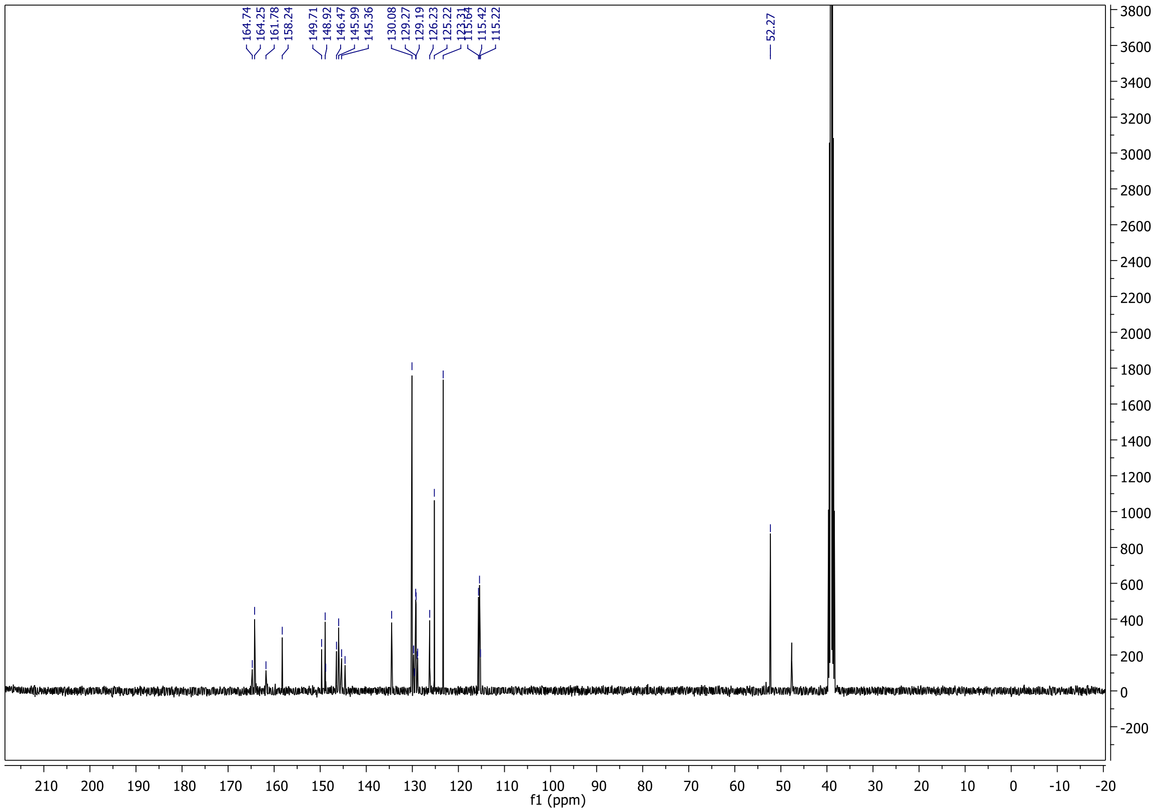

Figure S54. ^{13}CNMR of Compound 14

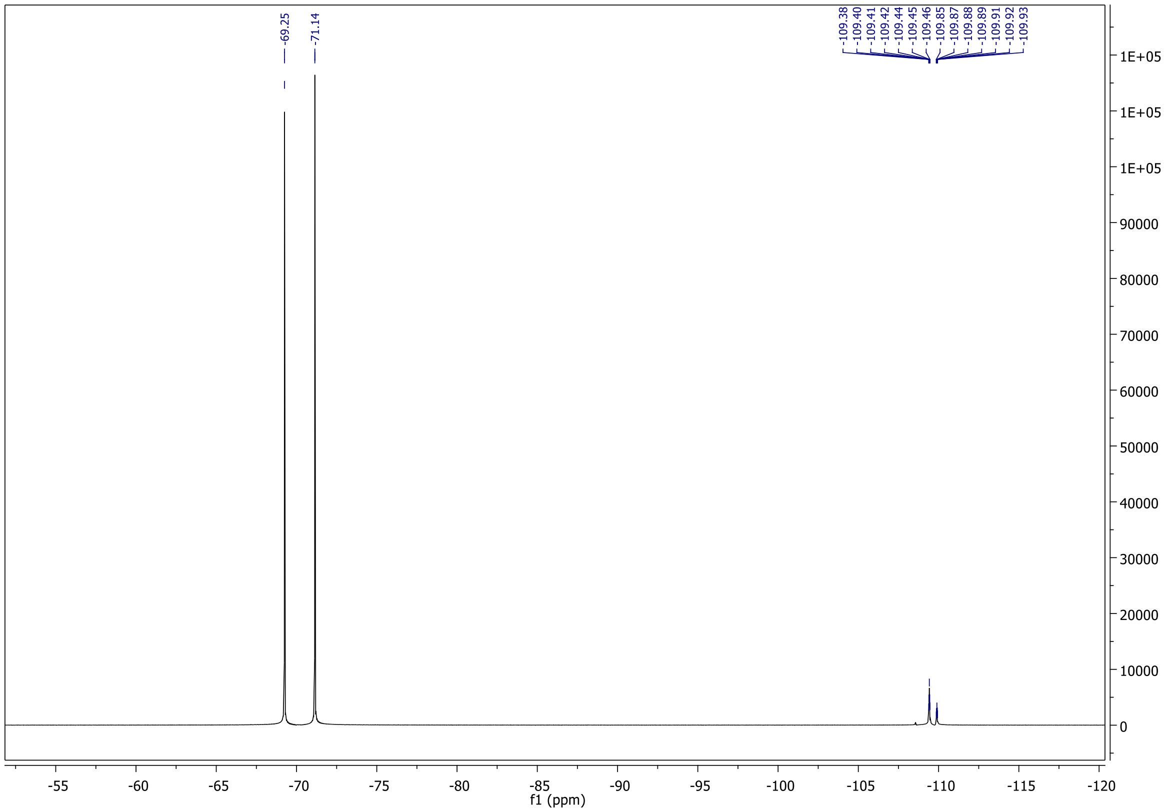

Figure S55. ^{19}FNMR of Compound 14

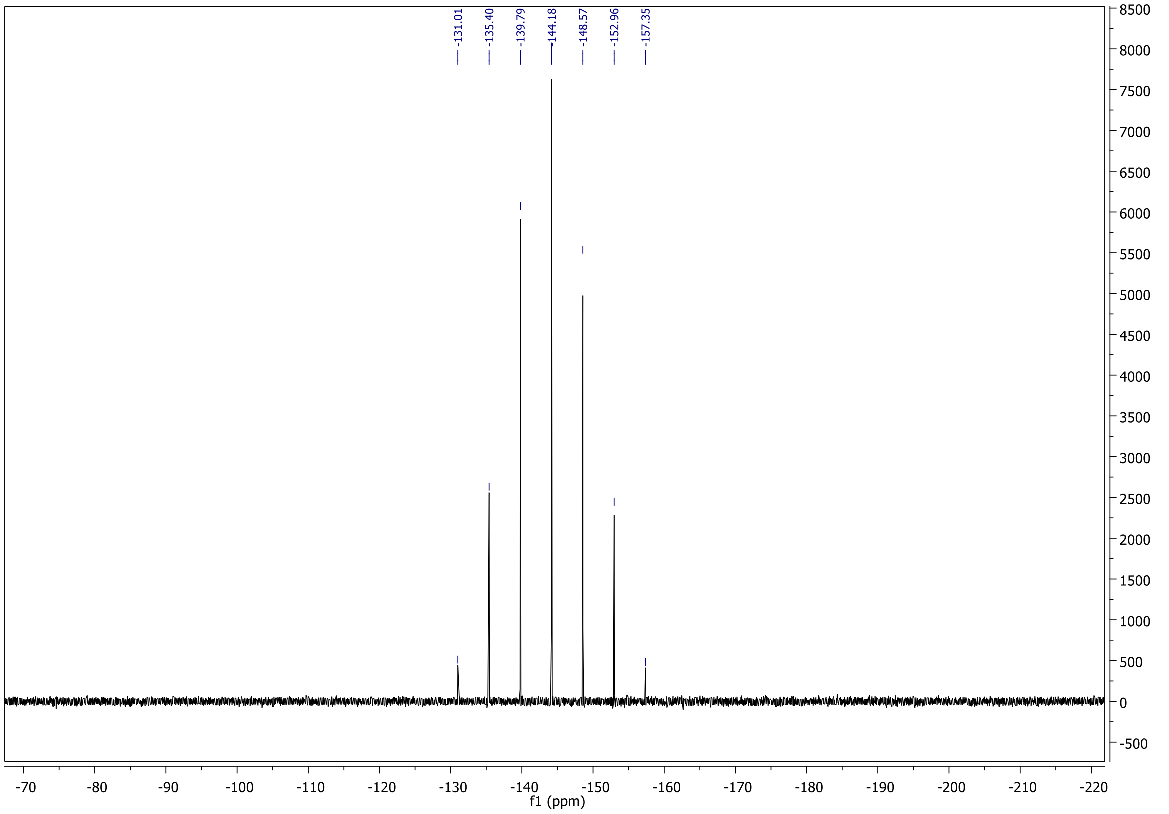

Figure S56. ^{31}PNMR of Compound 14

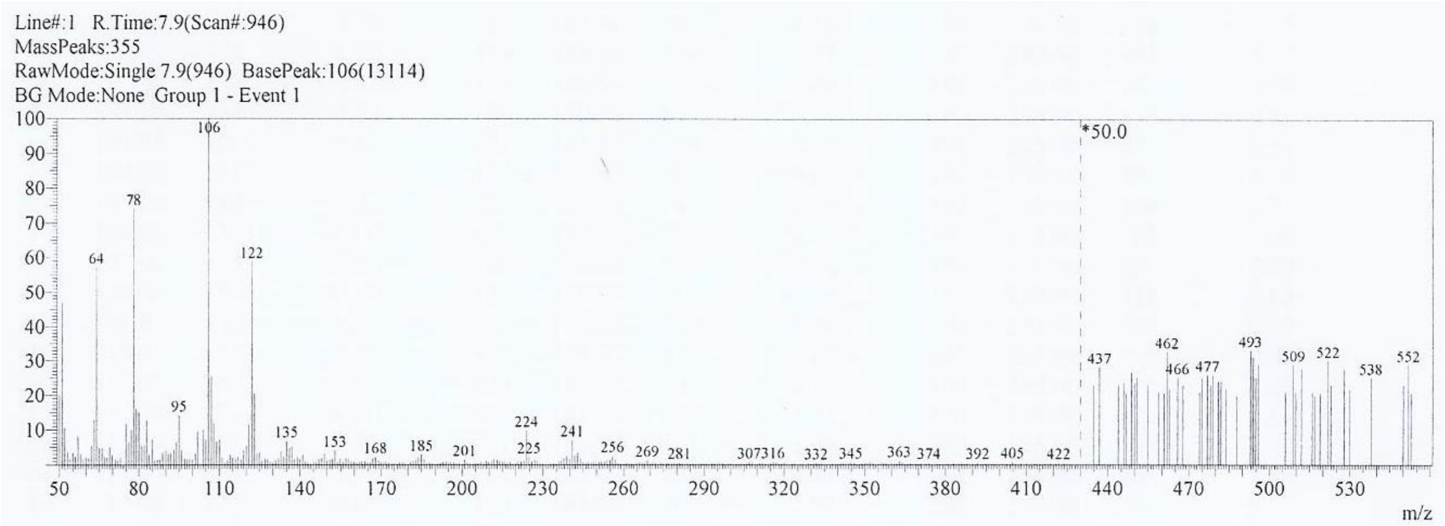

Figure S57. MS (ESI) of Compound 14

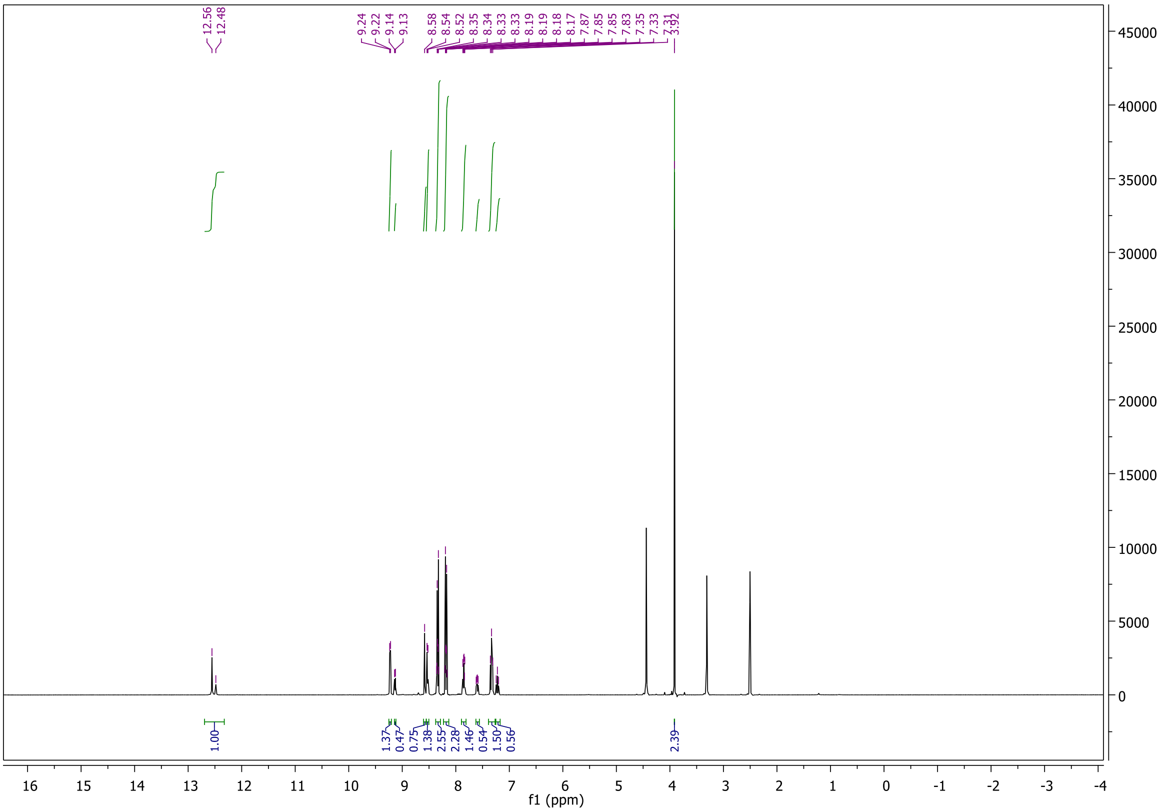

Figure S58. ^{1}HNMR of Compound 15

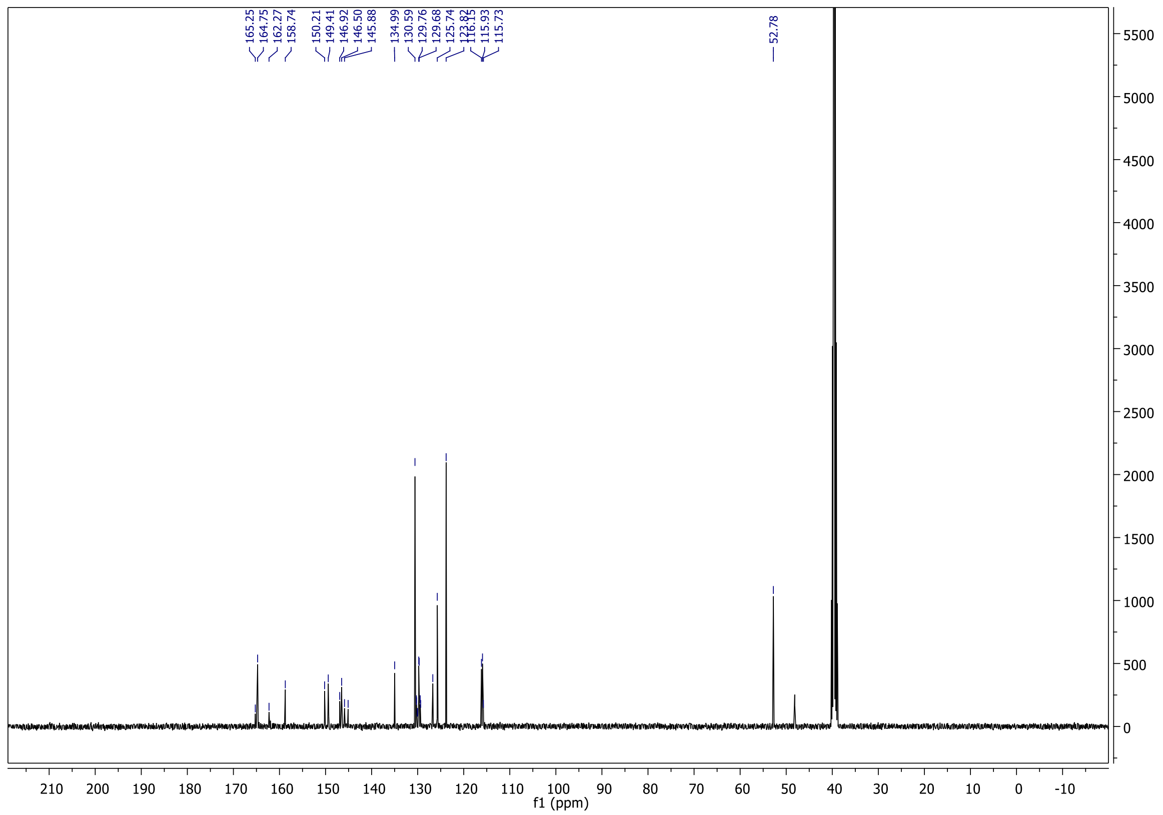

Figure S59. ^{13}CNMR of Compound 15

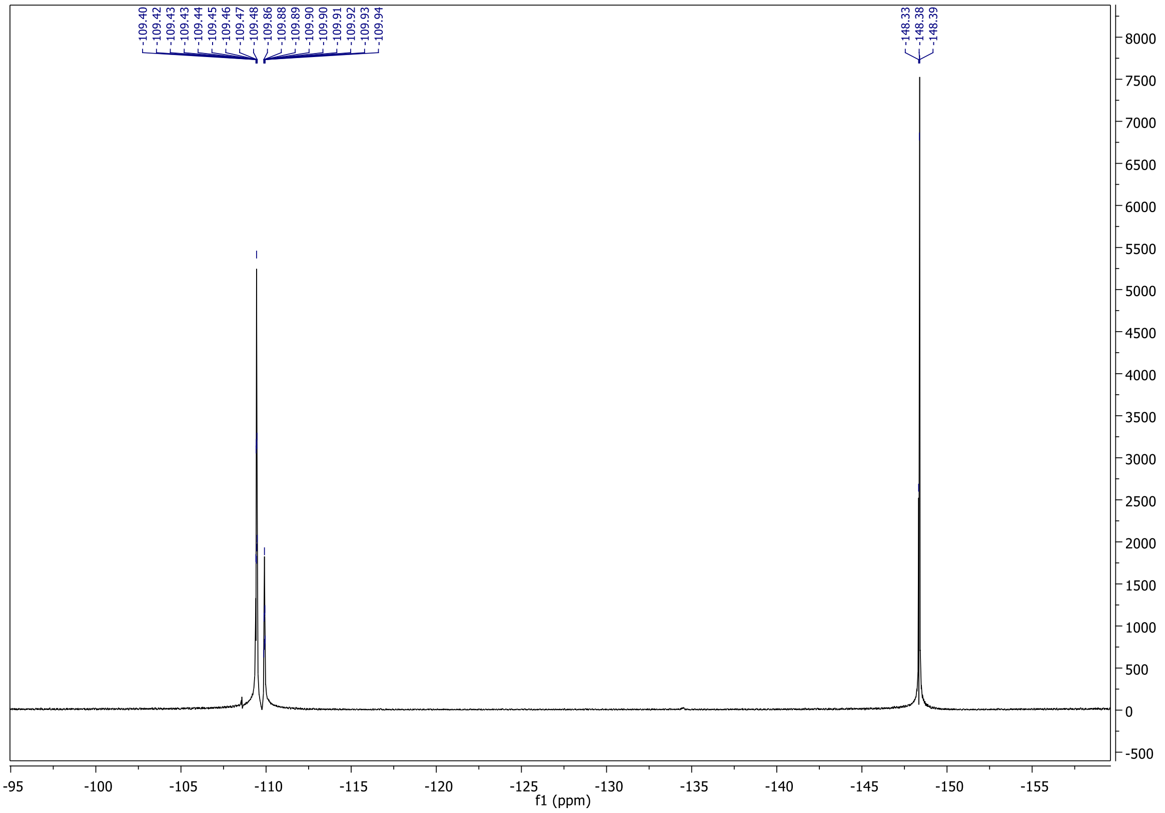

Figure S60. ^{19}FNMR of Compound 15

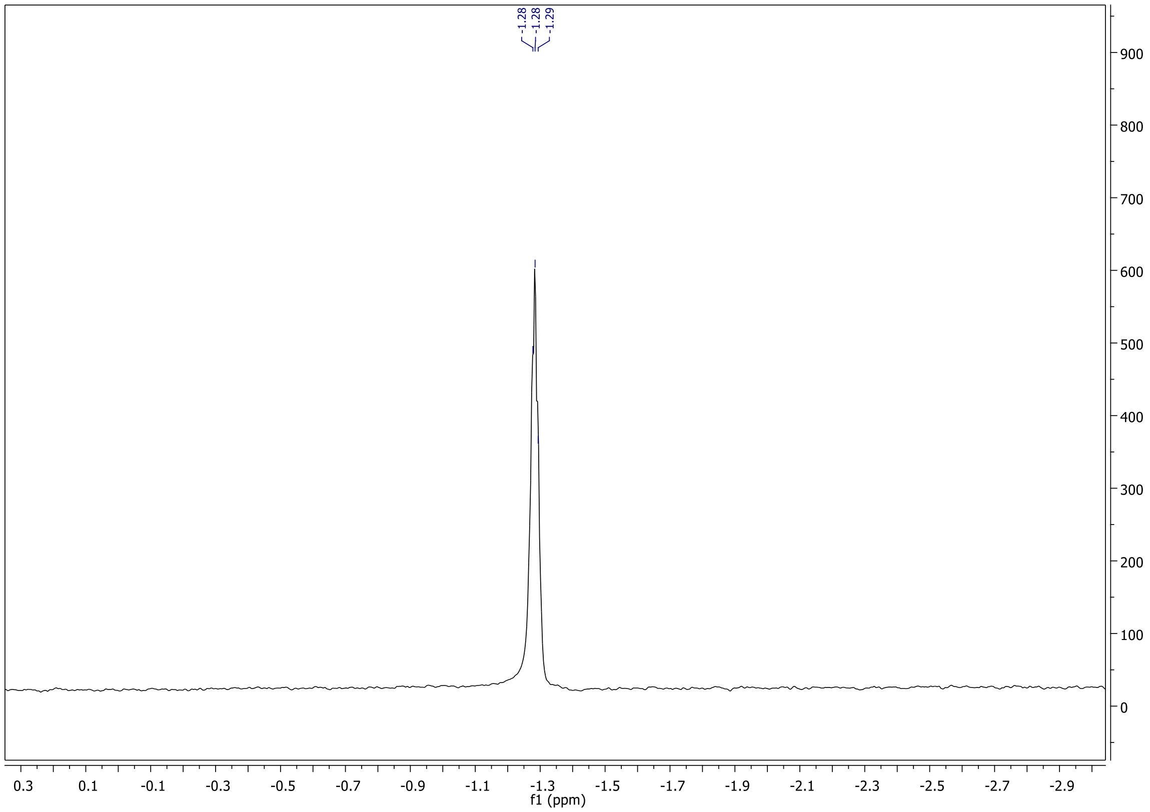

Figure S61. ^{11}BNMR of Compound 15

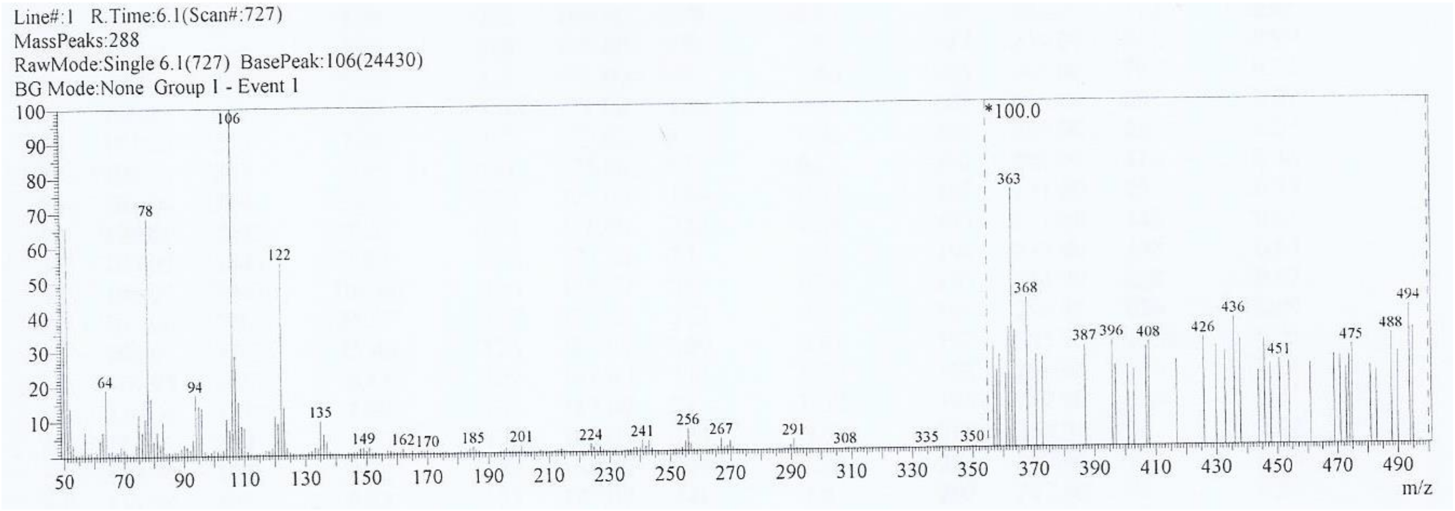

Figure S62. MS (ESI) of Compound 15

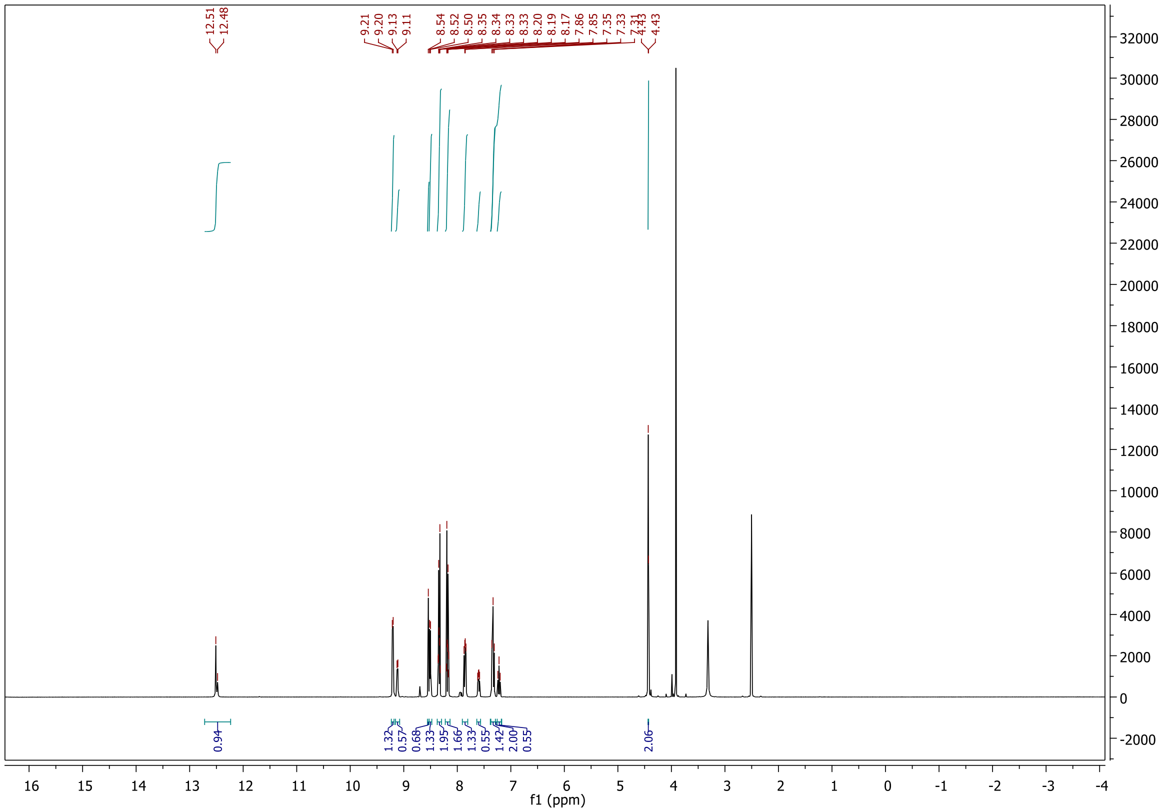

Figure S63. ^{1}HNMR of Compound 16

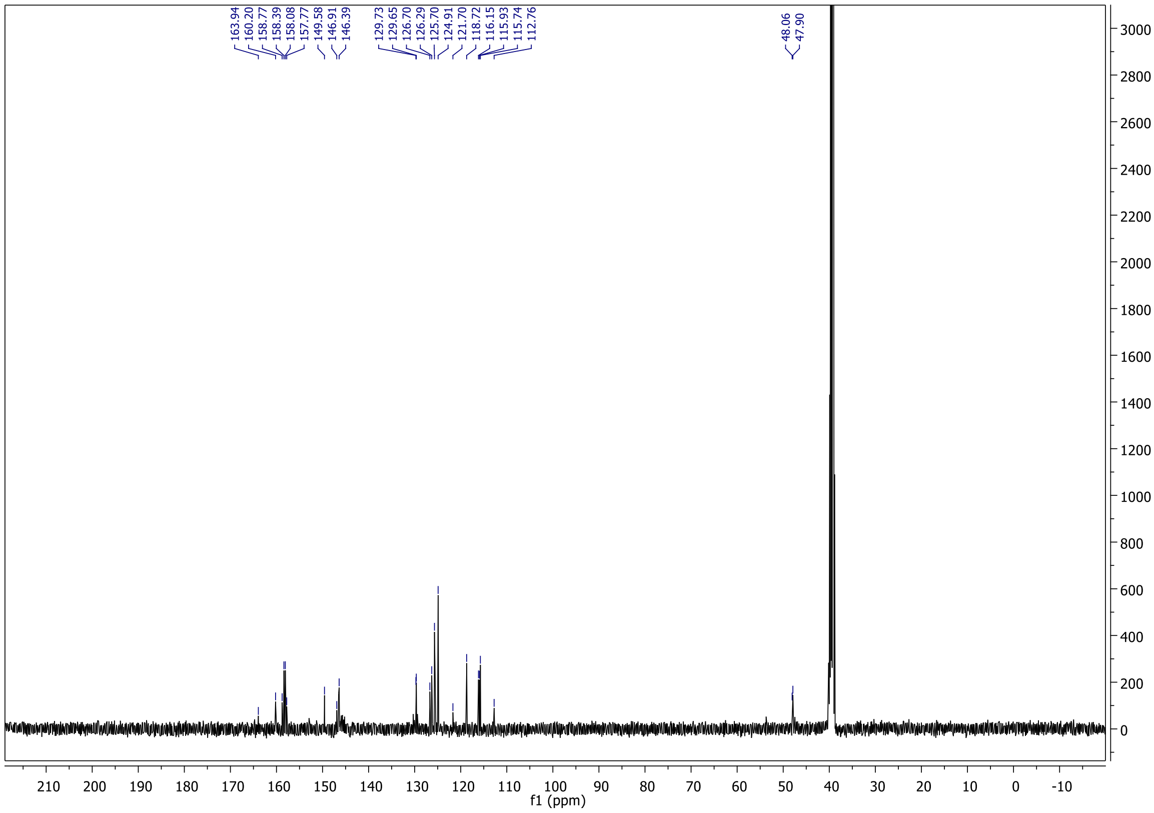

Figure S64. ^{13}CNMR of Compound 16

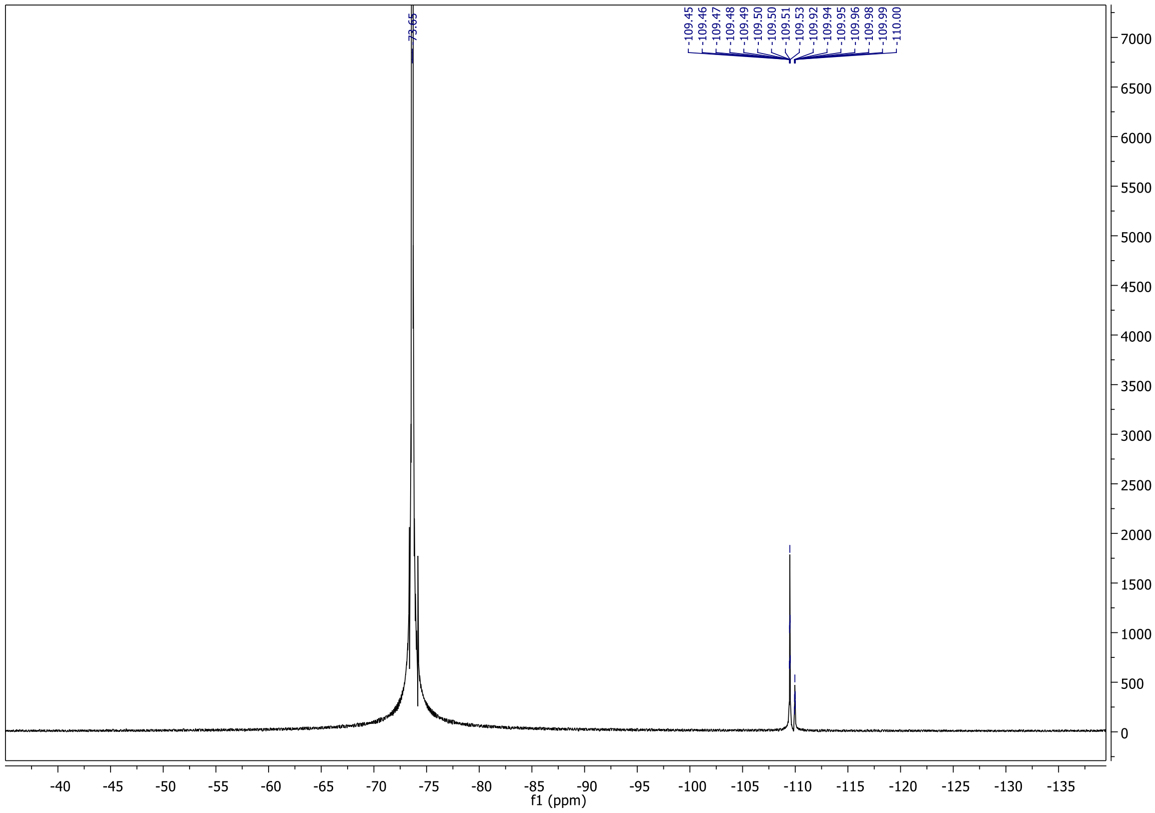

Figure S65. ^{19}FNMR of Compound 16

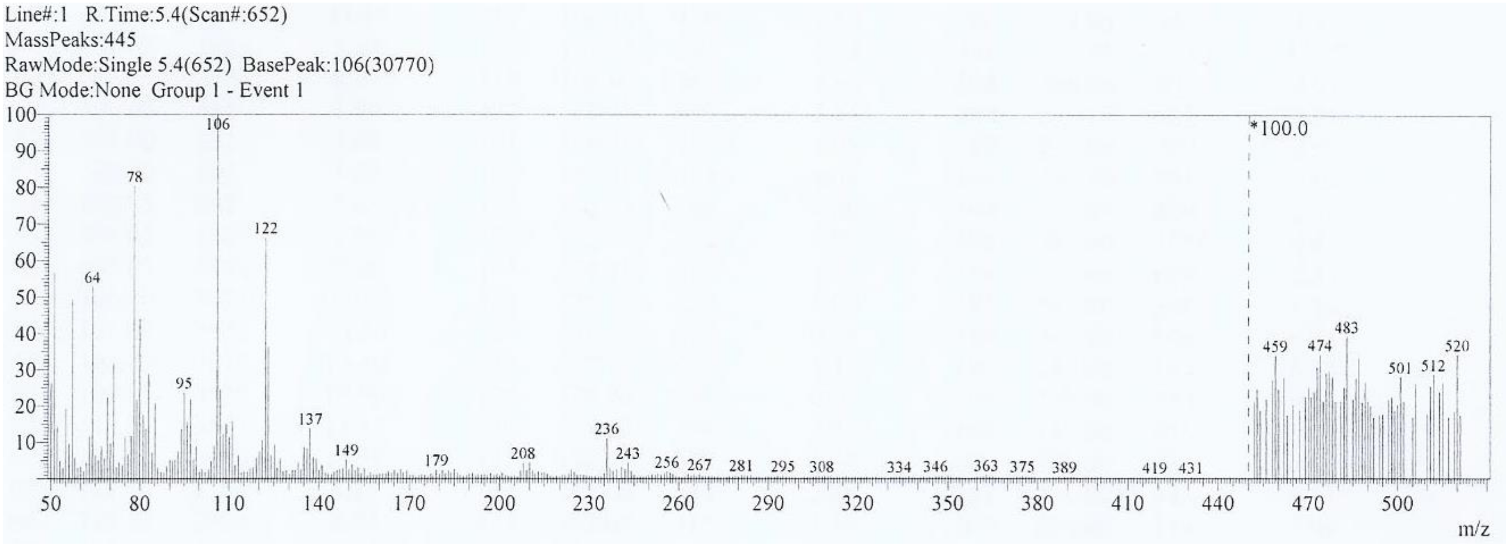

Figure S66. MS (ESI) of Compound 16

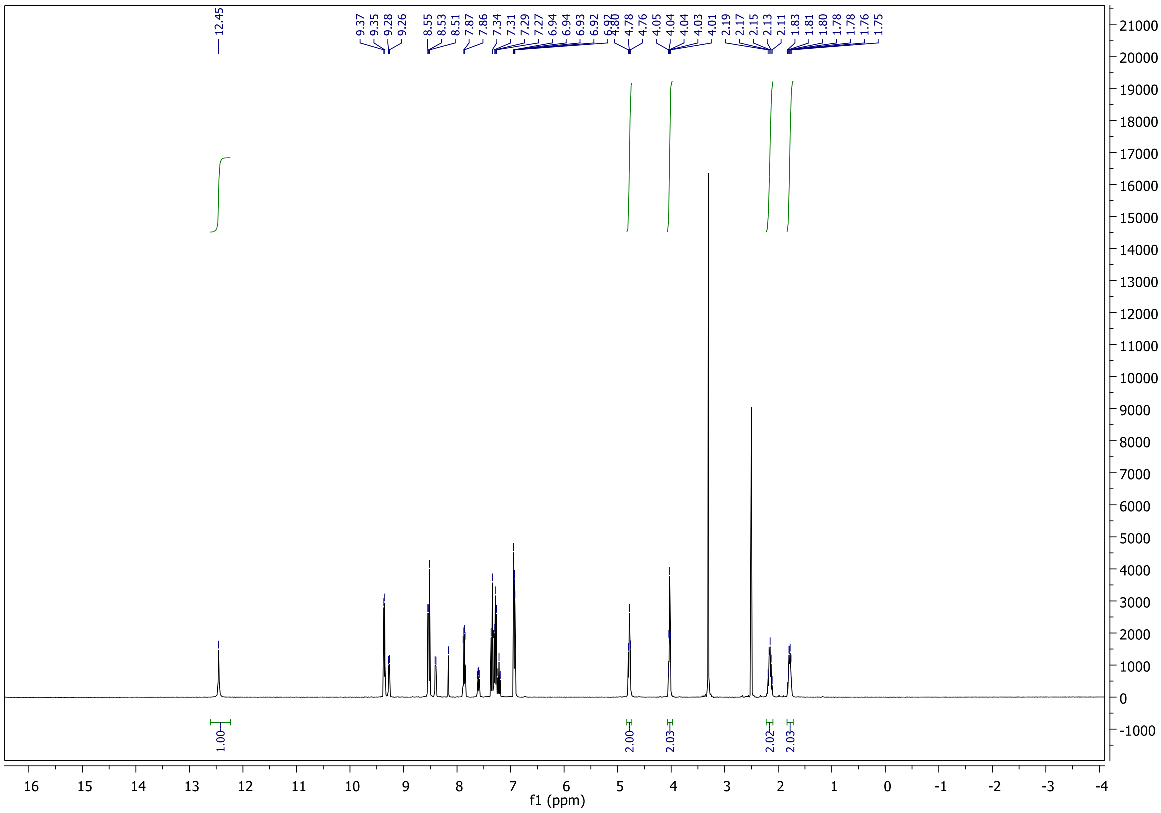

Figure S67. ^{1}HNMR of Compound 17

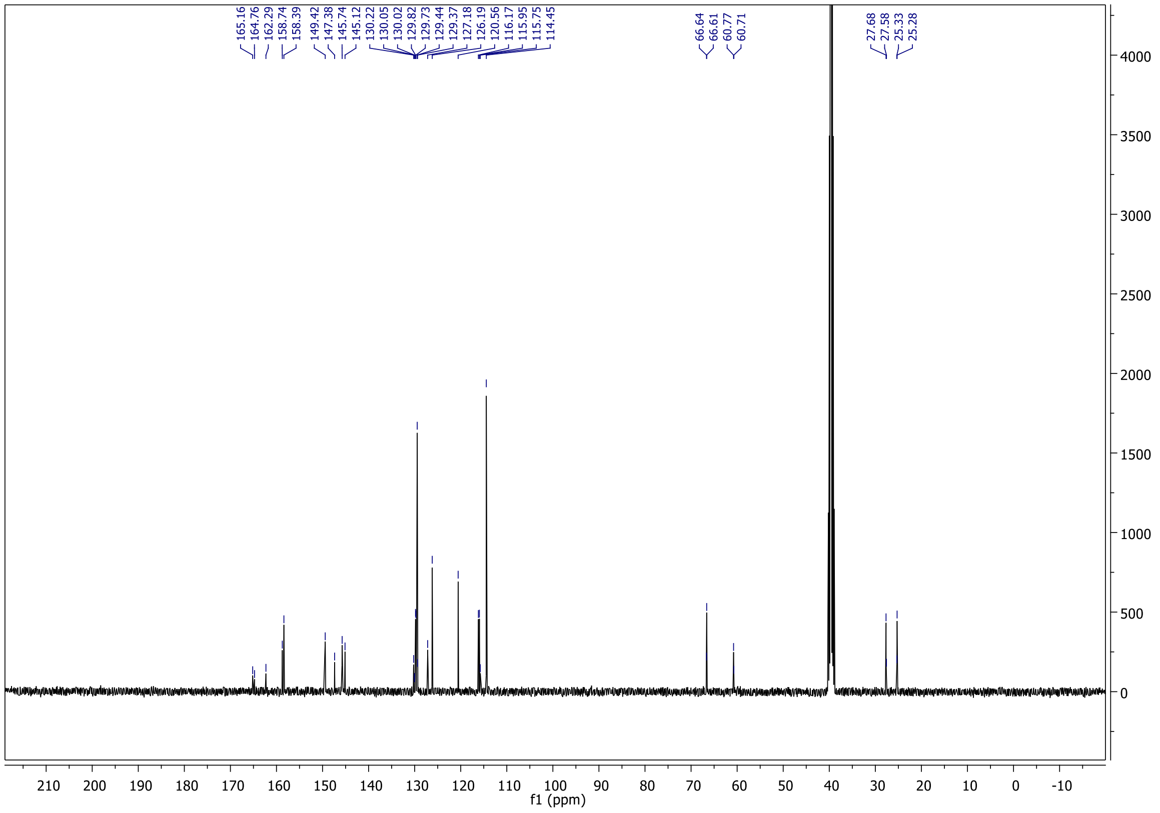

Figure S68. ^{13}CNMR of Compound 17

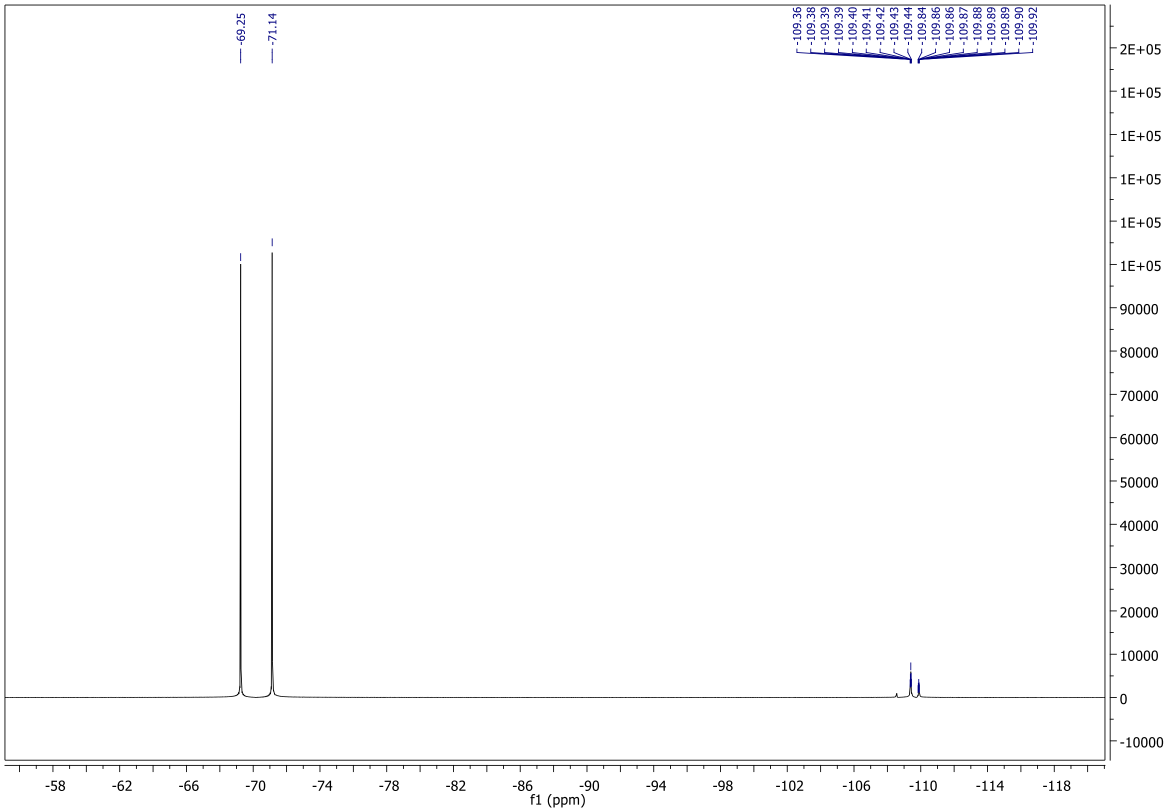

Figure S69. ^{19}FNMR of Compound 17

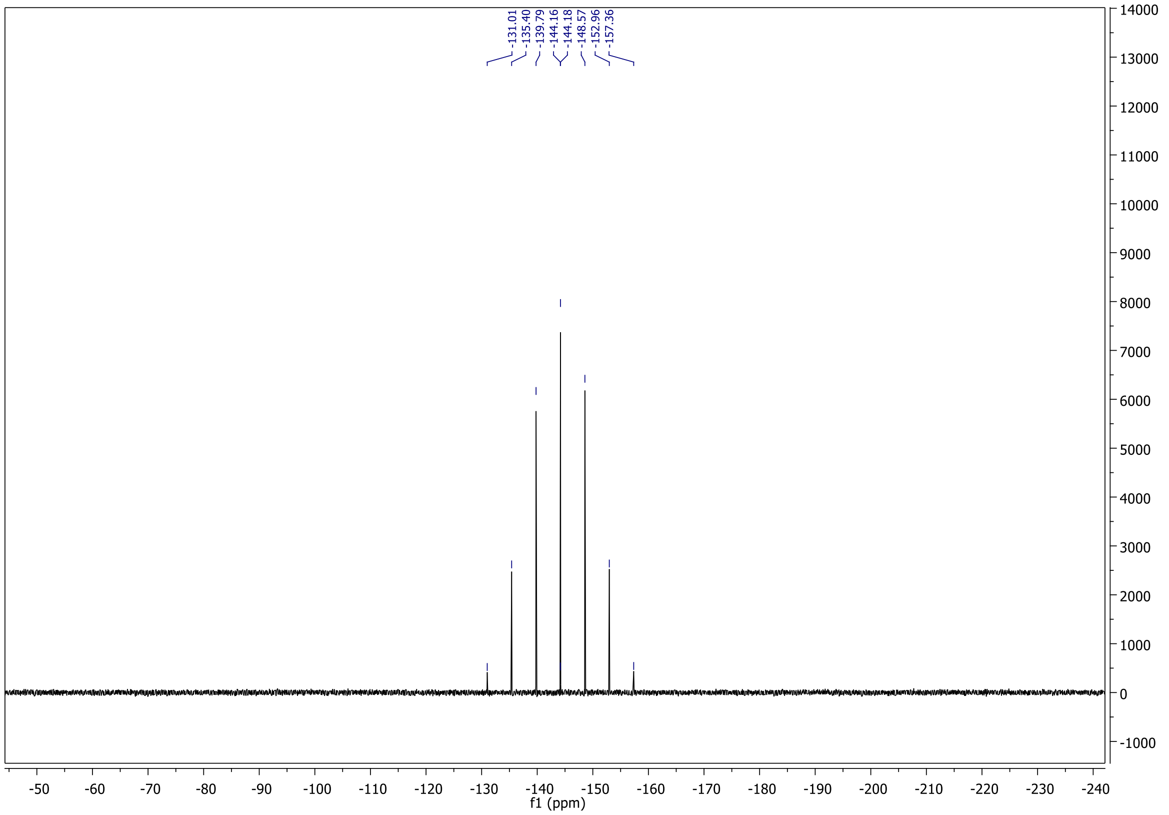

Figure S70. ^{31}PNMR of Compound 17

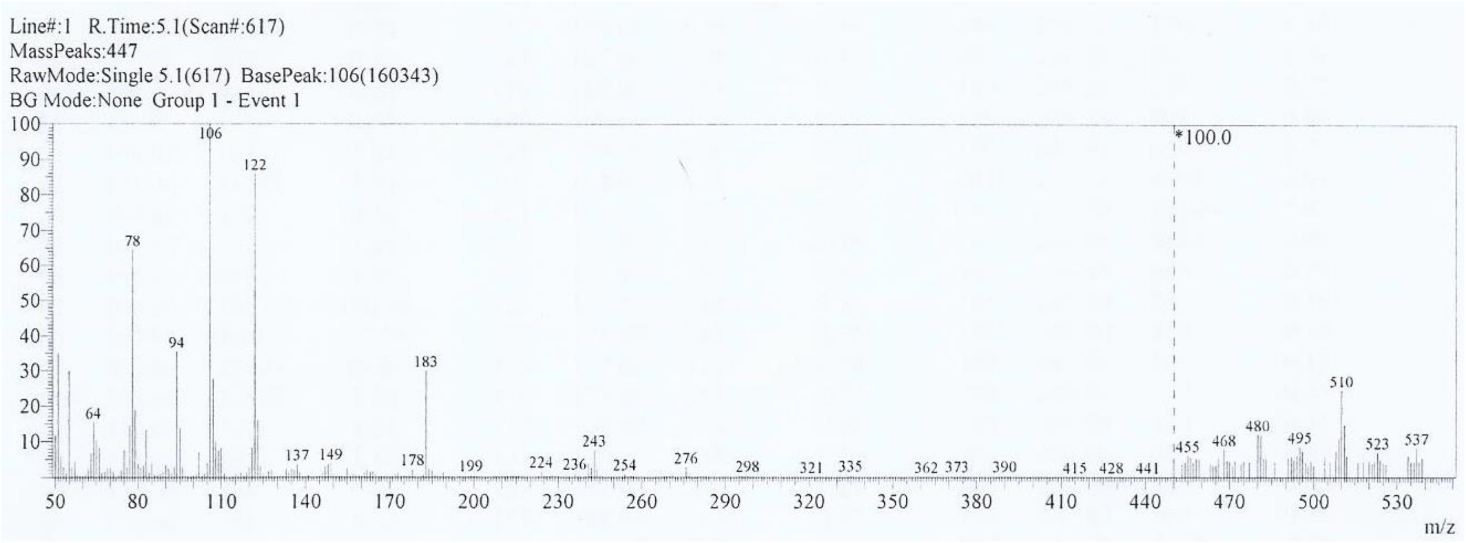

Figure S71. MS (ESI) of Compound 17

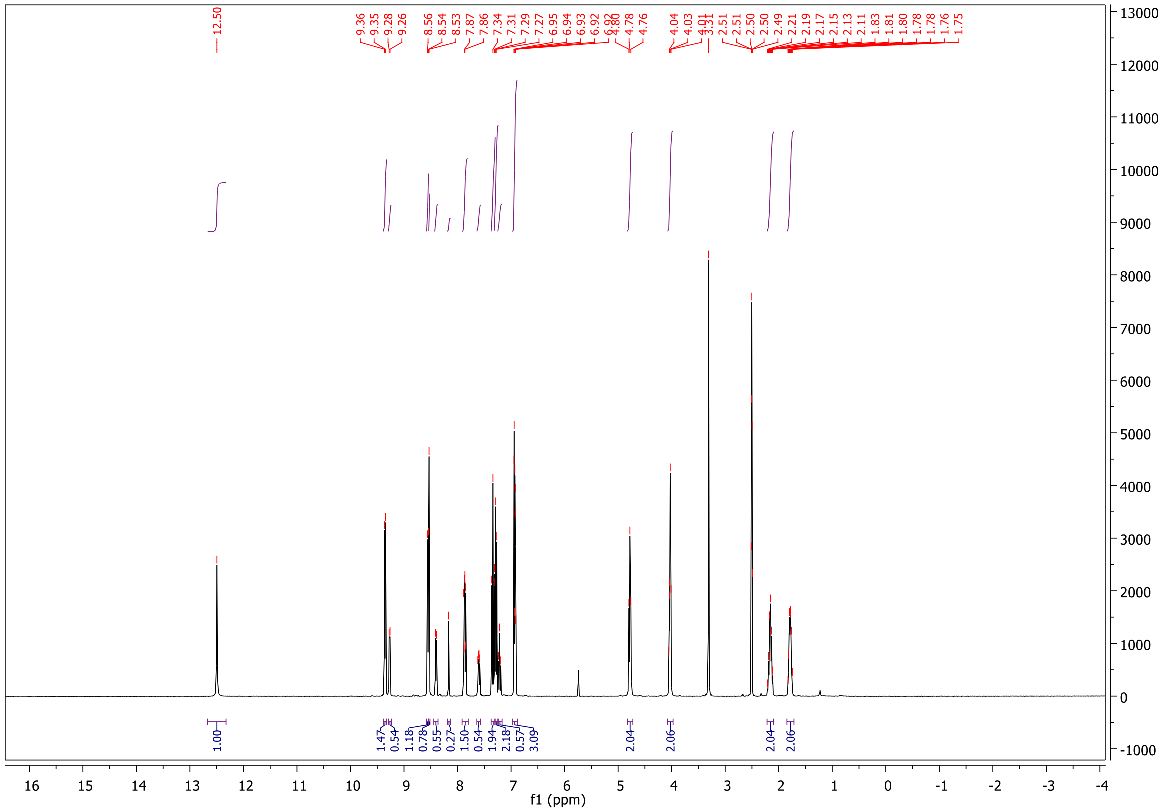

Figure S72. ^{1}HNMR of Compound 18

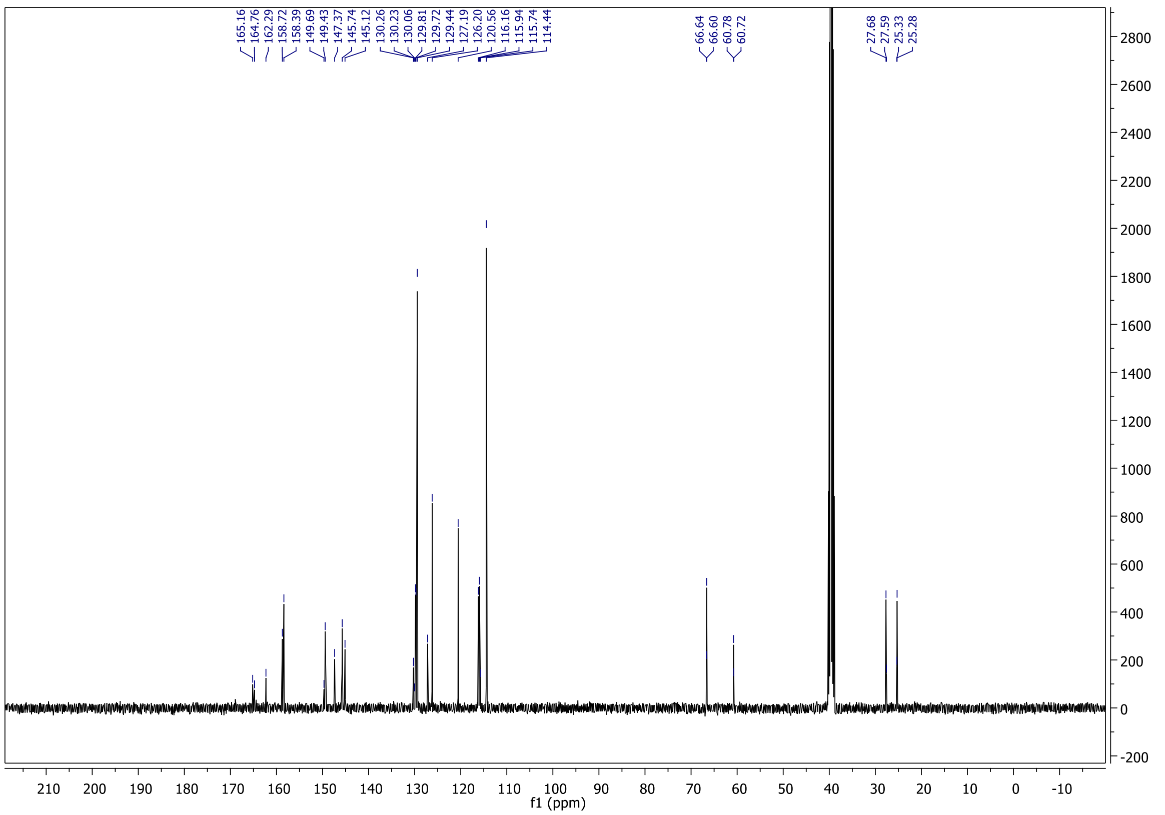

Figure S73. ^{13}CNMR of Compound 18

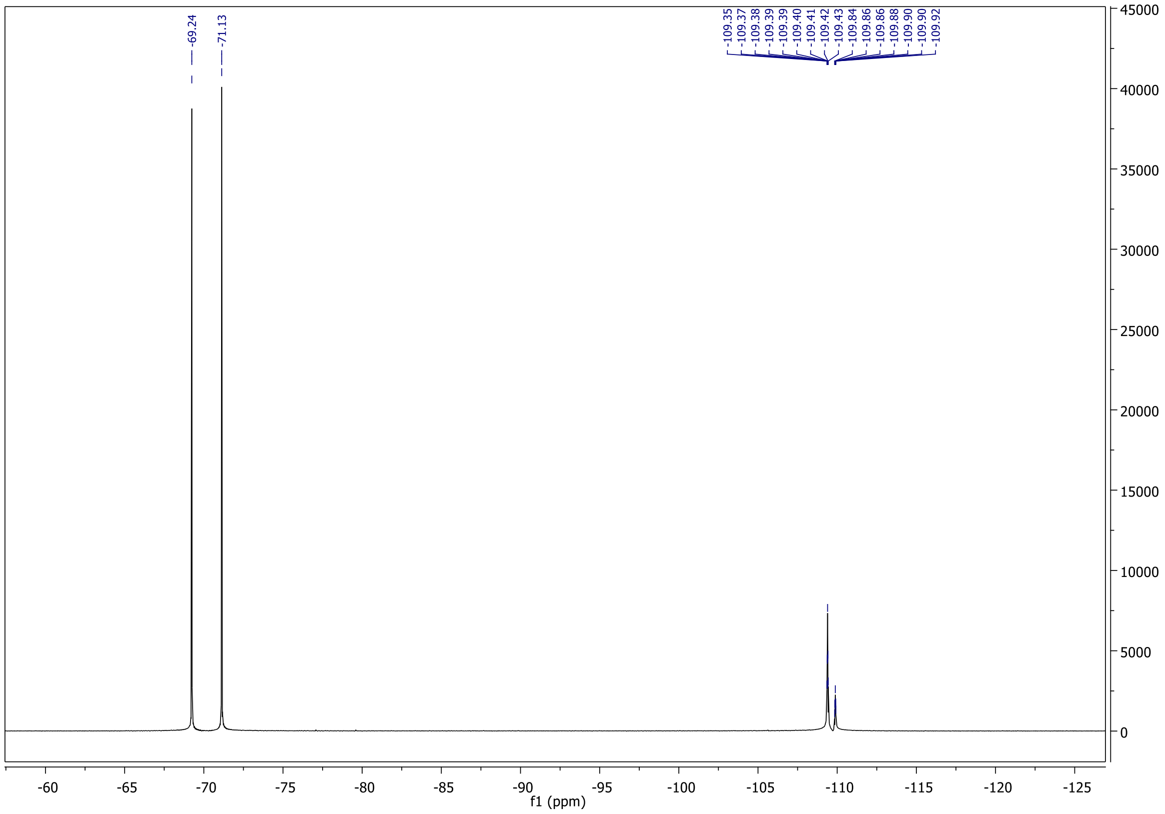

Figure S74. ^{19}FNMR of Compound 18

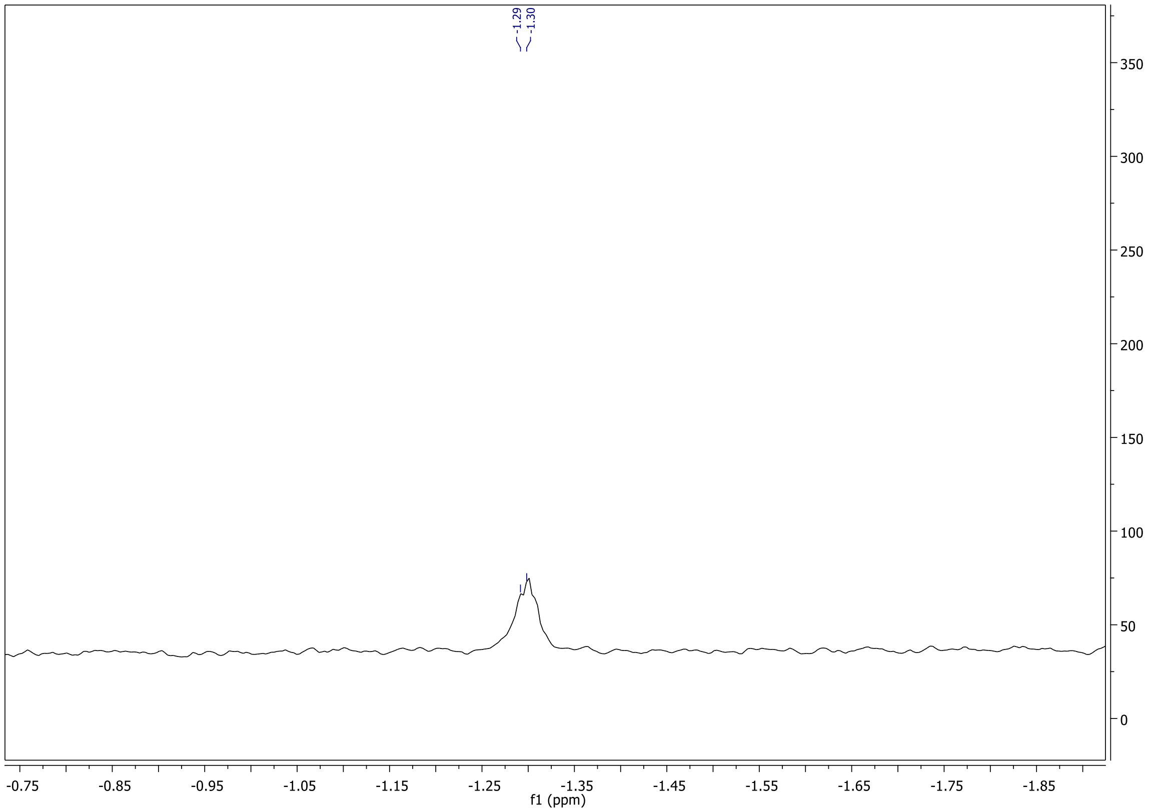

Figure S75. ^{11}BNMR of Compound 18

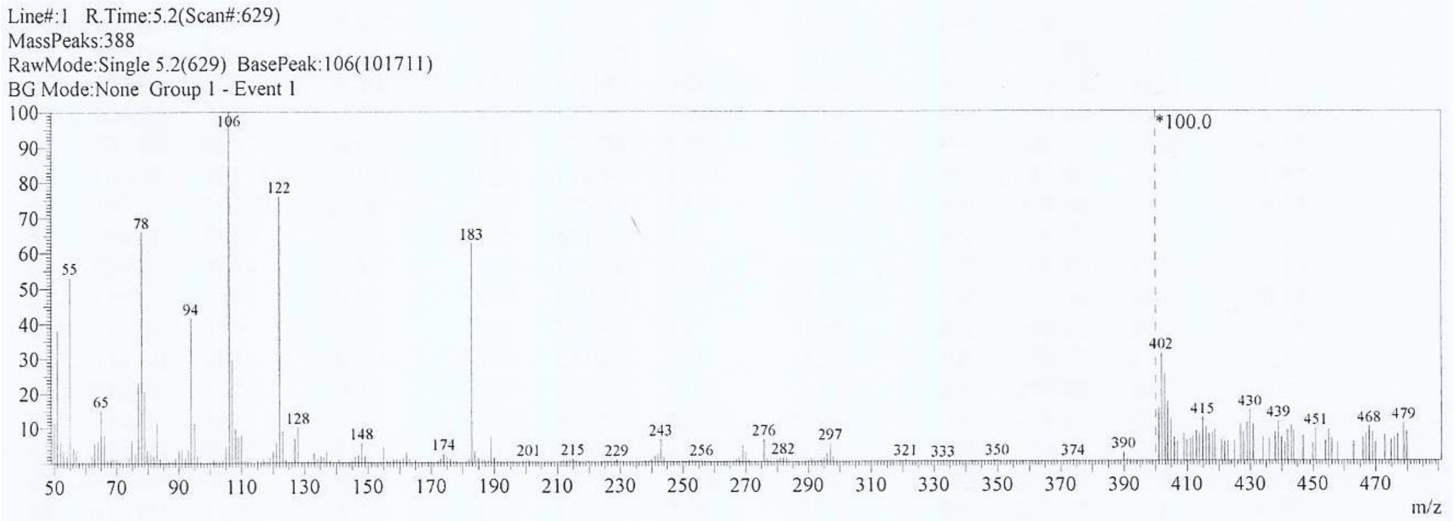

Figure S76. MS (ESI) of Compound 18

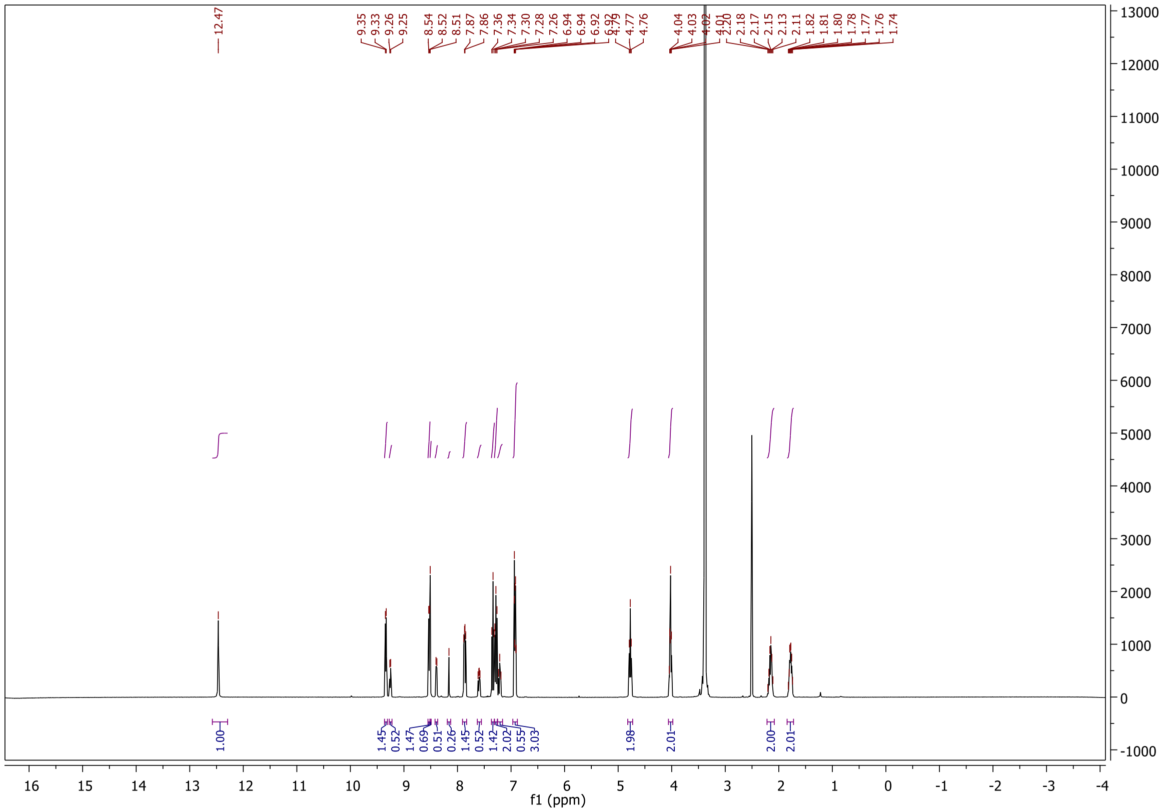

Figure S77. ^{1}HNMR of Compound 19

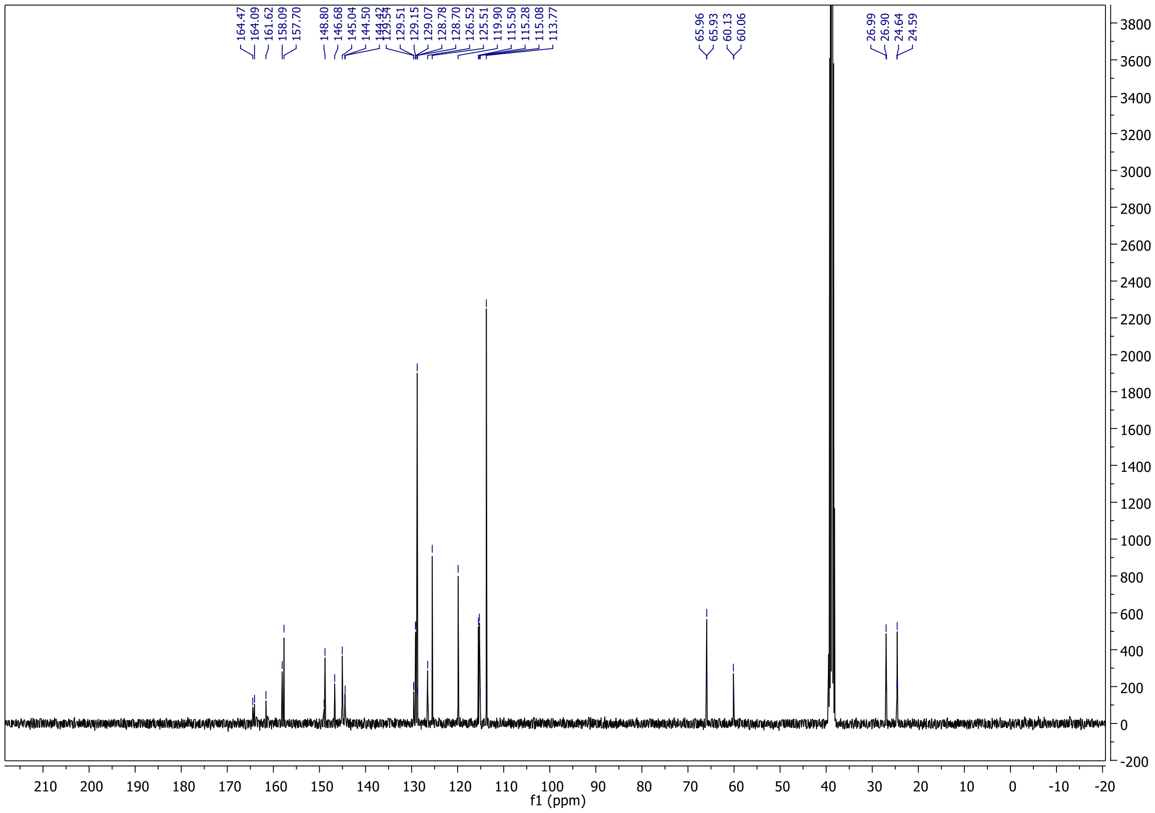

Figure S78. ^{13}CNMR of Compound 19

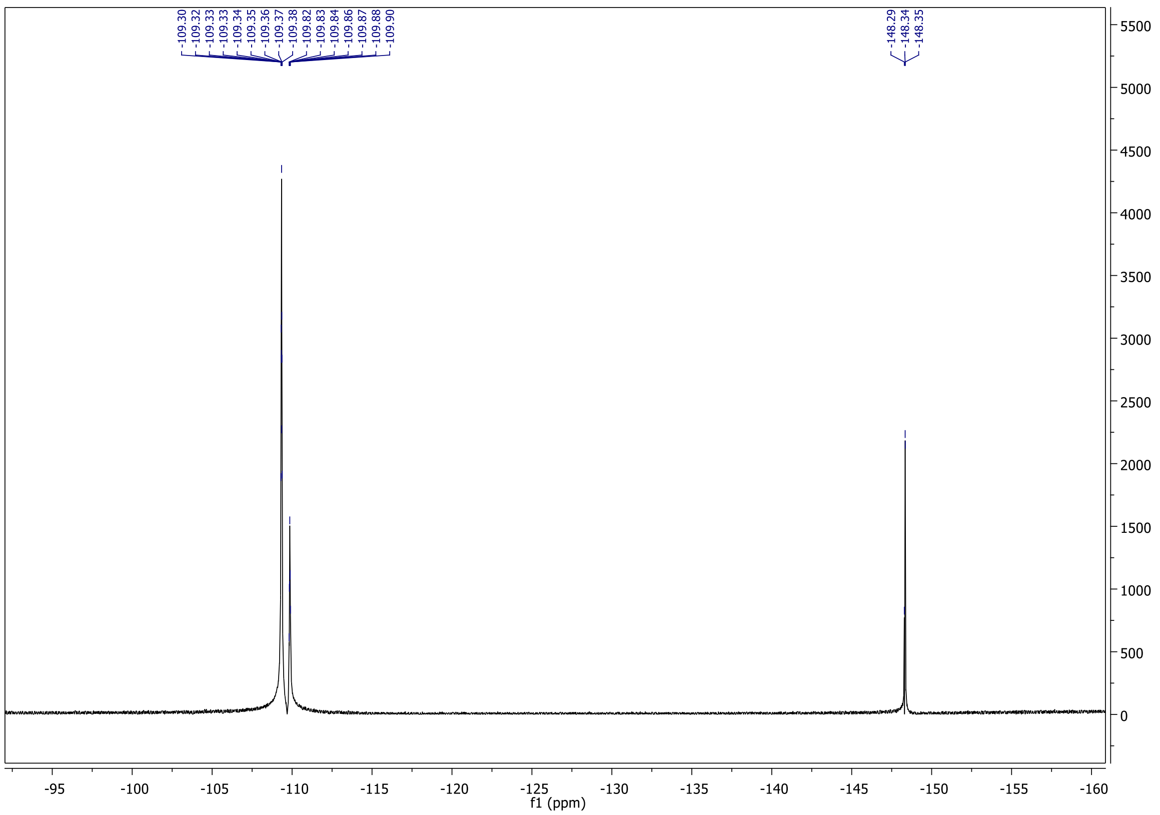

Figure S79. ^{19}FNMR of Compound 19

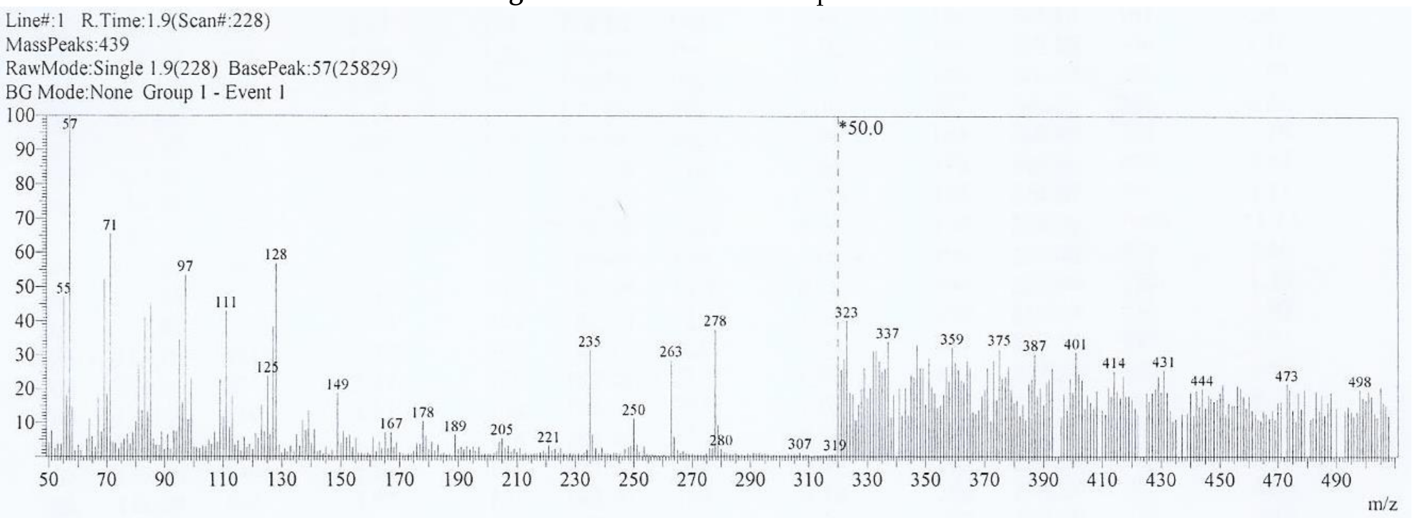

Figure S80. MS (ESI) of Compound 19

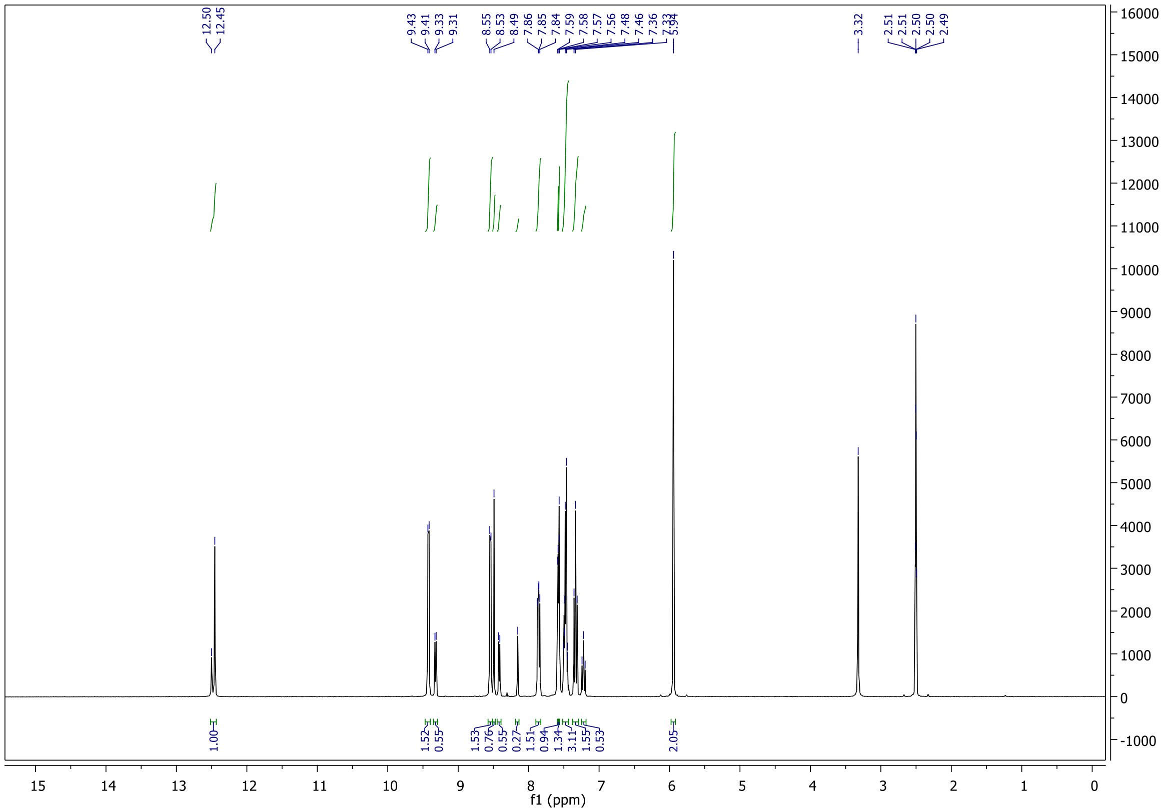

Figure S81. ^{1}HNMR of Compound 20

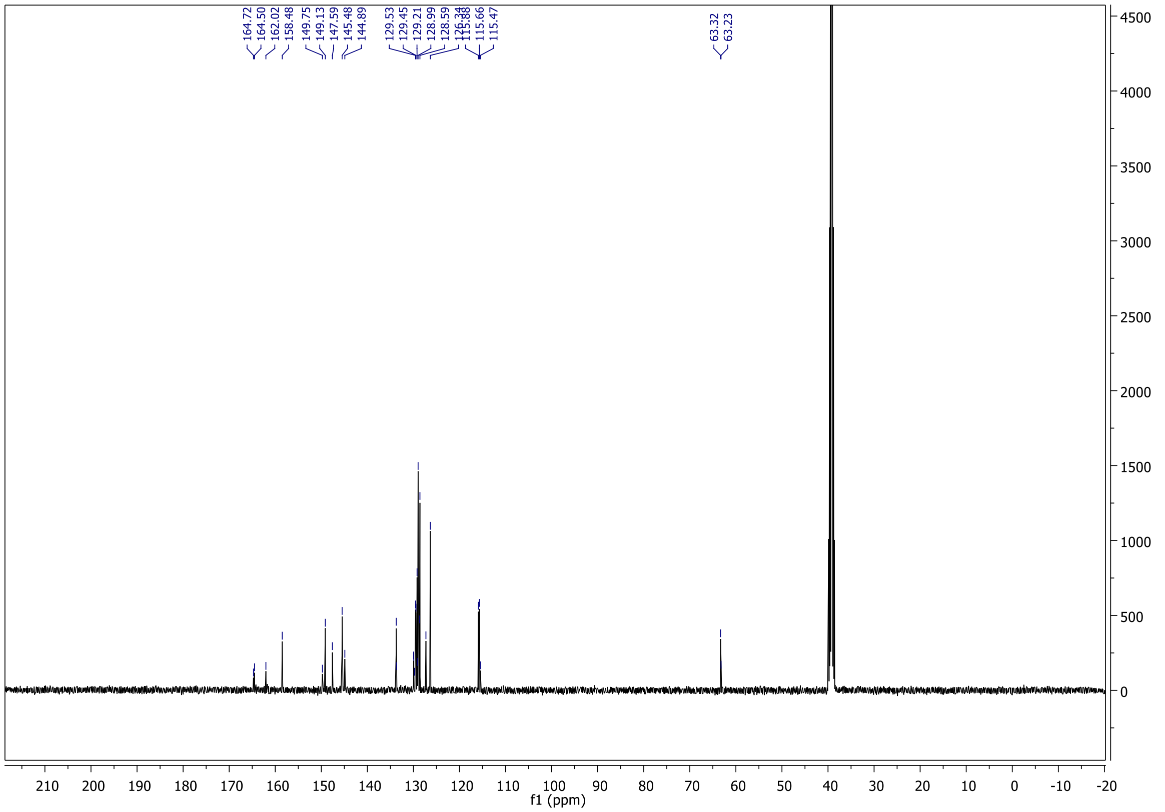

Figure S82. ^{13}CNMR of Compound 20

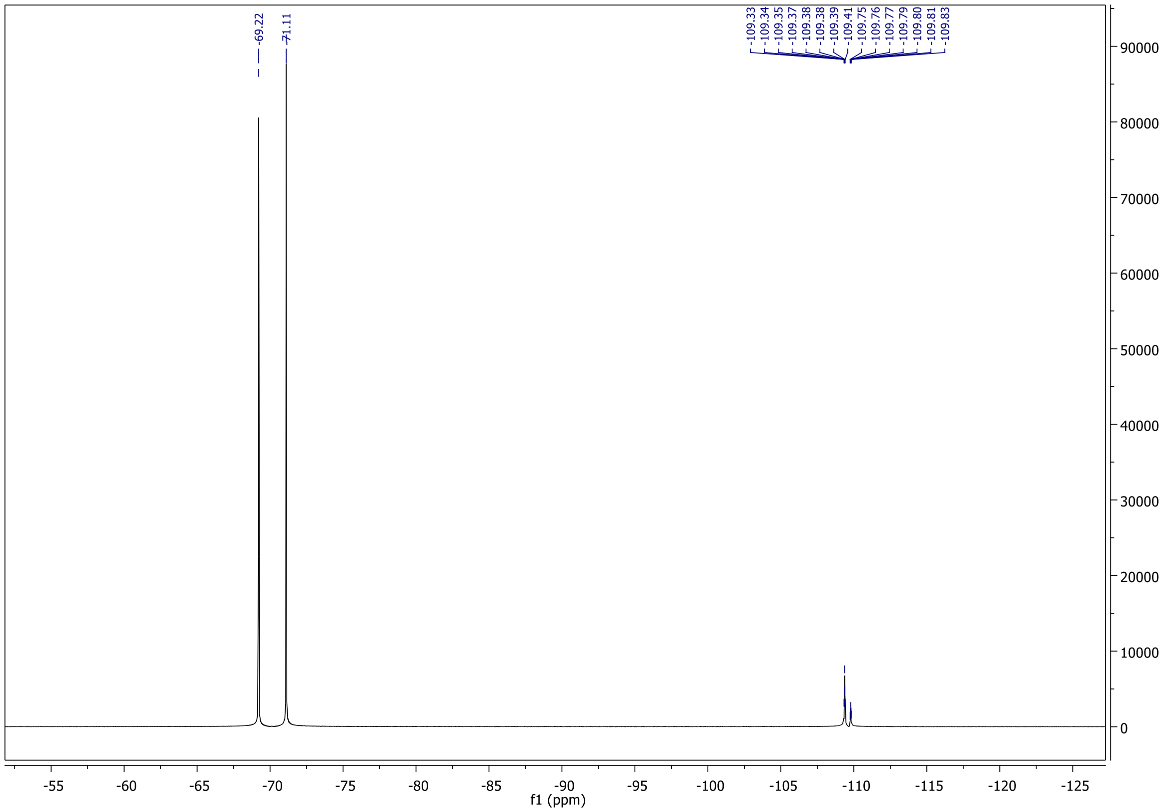

Figure S83. ^{19}FNMR of Compound 20

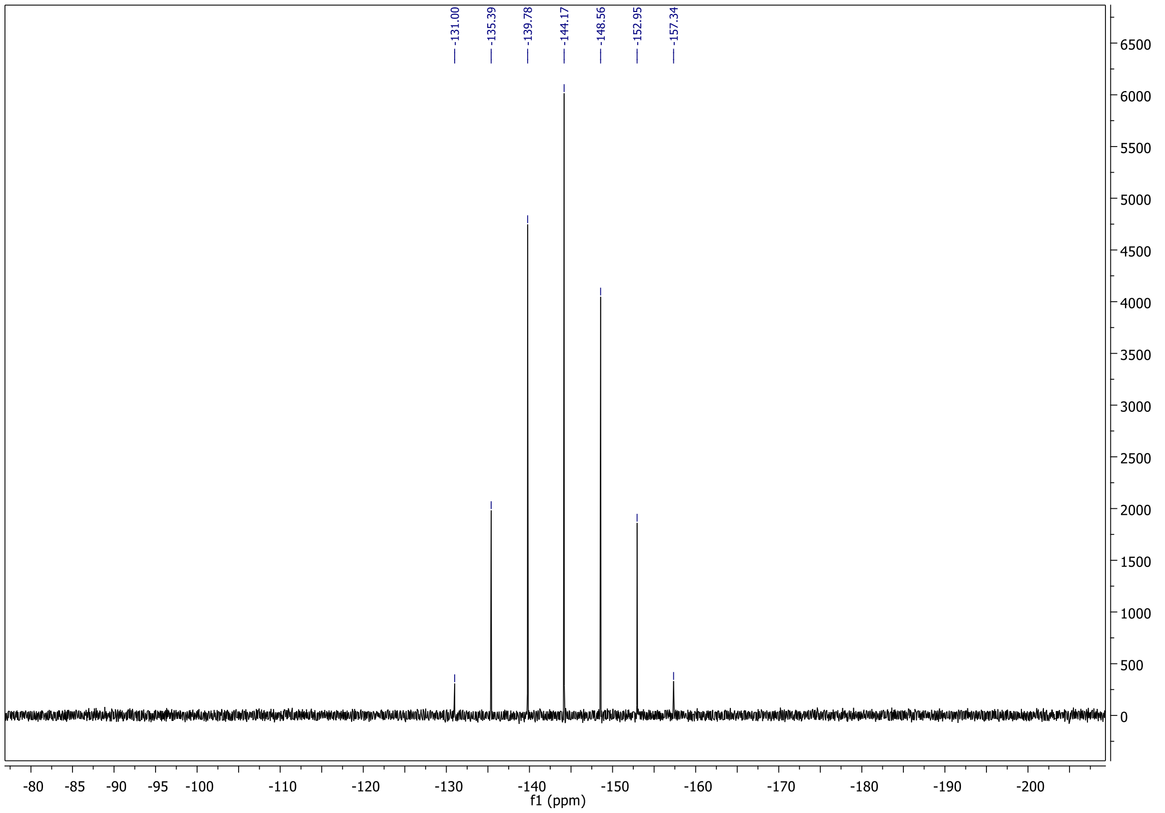

Figure S84. ^{31}PNMR of Compound 20

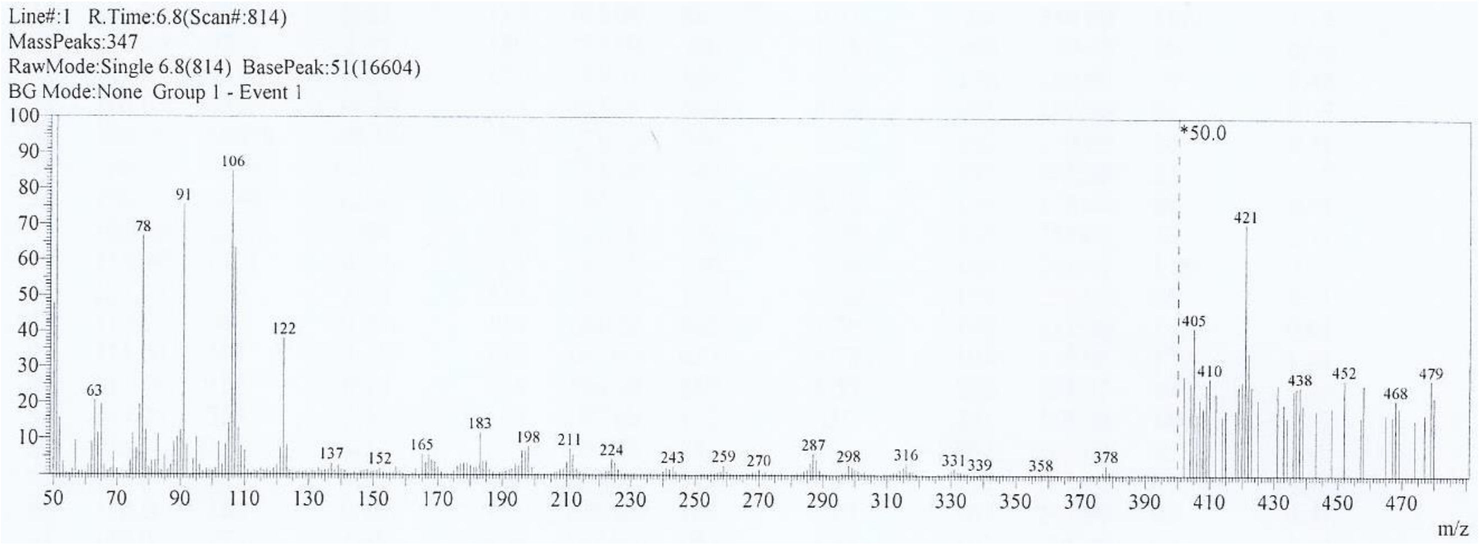

Figure S85. MS (ESI) of Compound 20

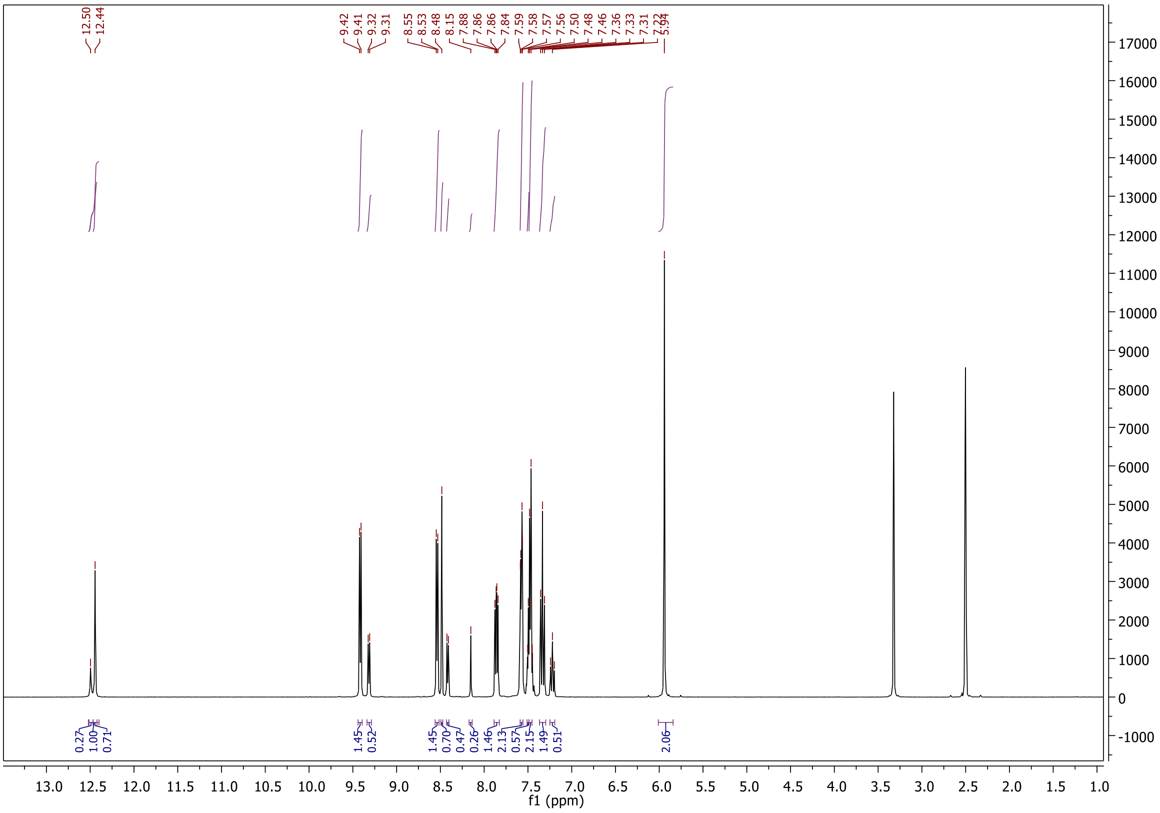

Figure S86. ^{1}HNMR of Compound 21

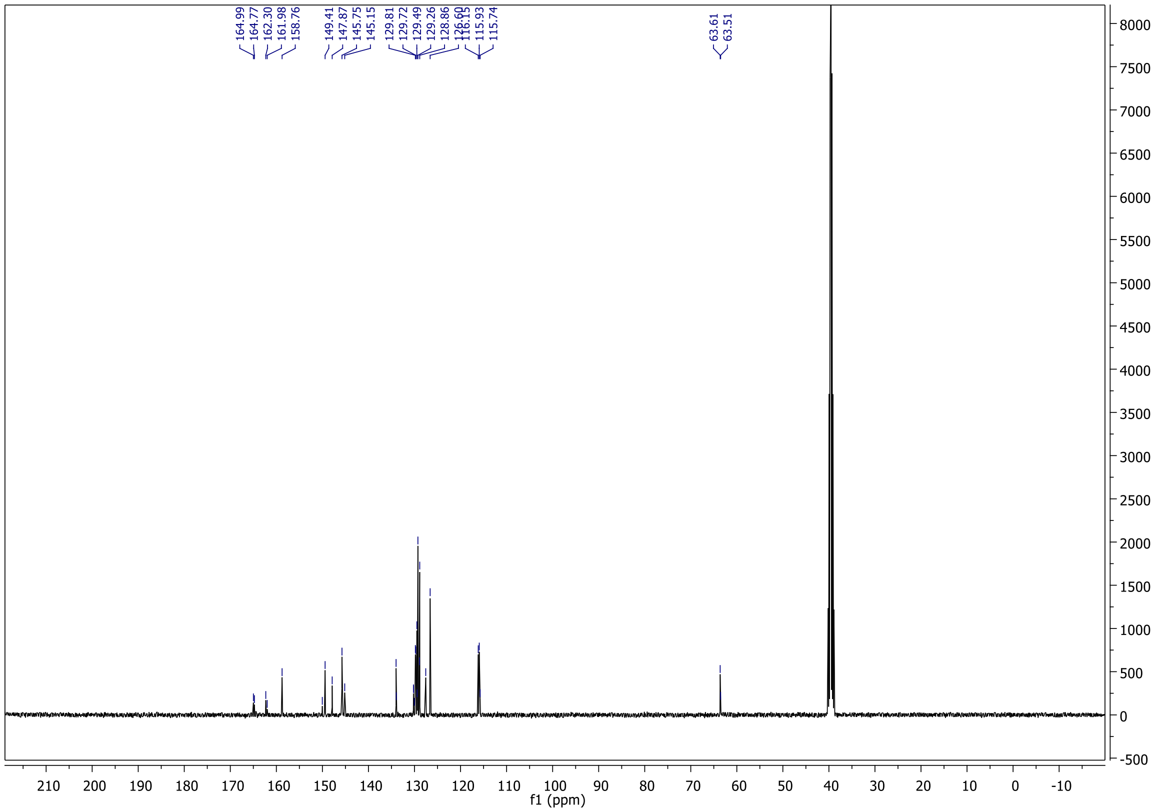

Figure S87. ^{13}CNMR of Compound 21

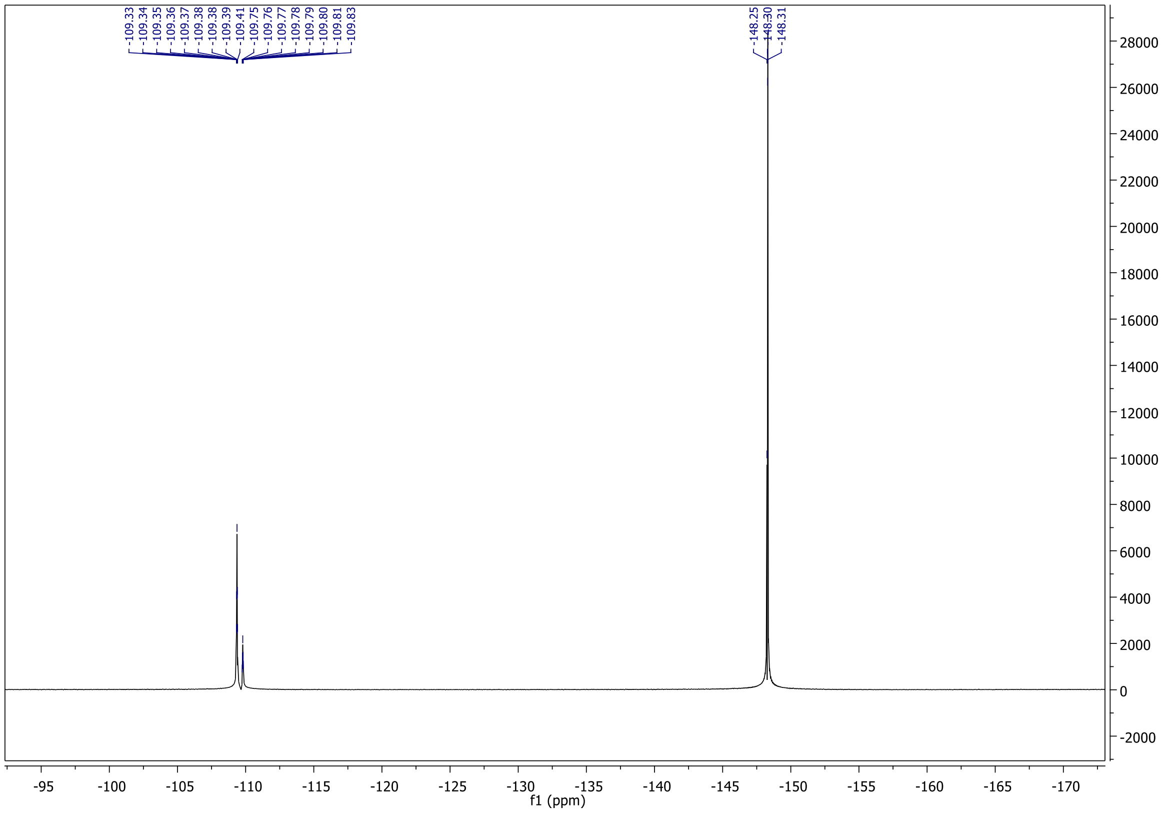

Figure S88. ^{19}FNMR of Compound 21

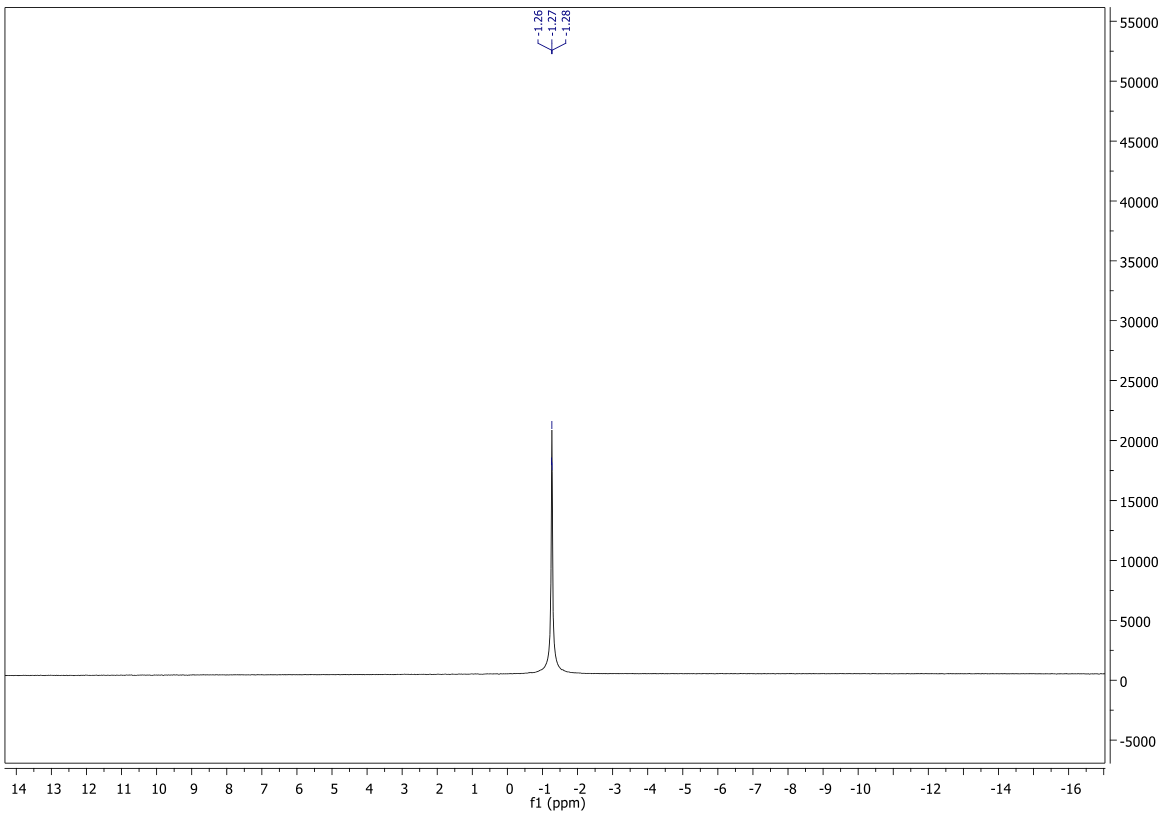

Figure S89. ^{11}BNMR of Compound 21

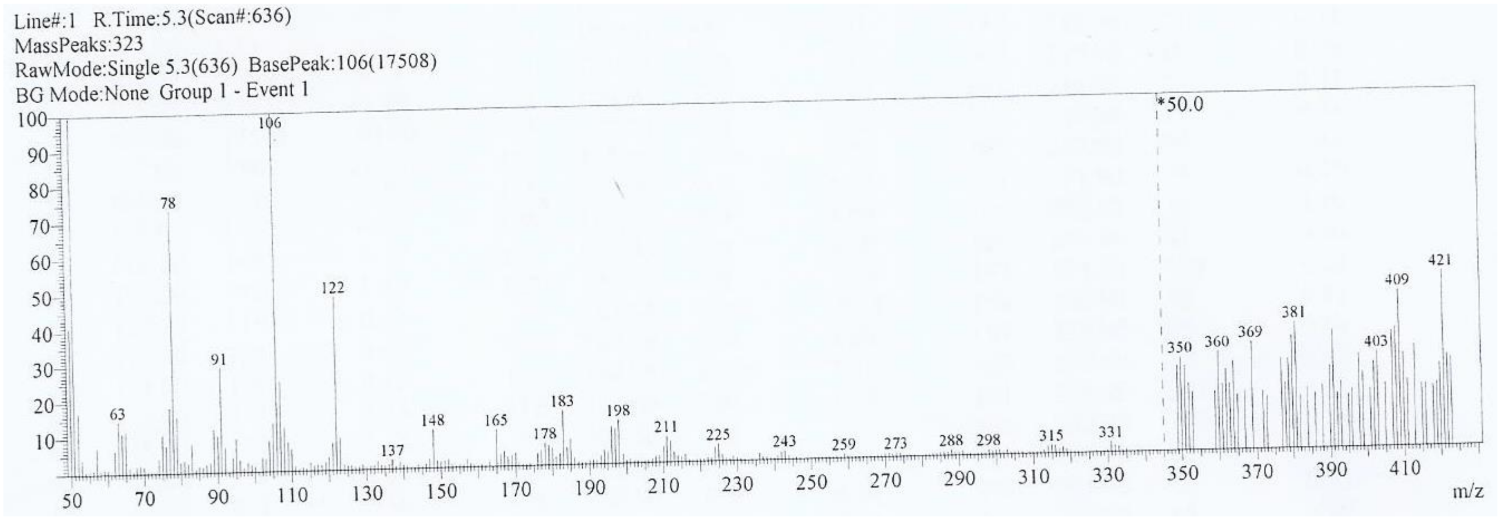

Figure S90. MS (ESI) of Compound 21

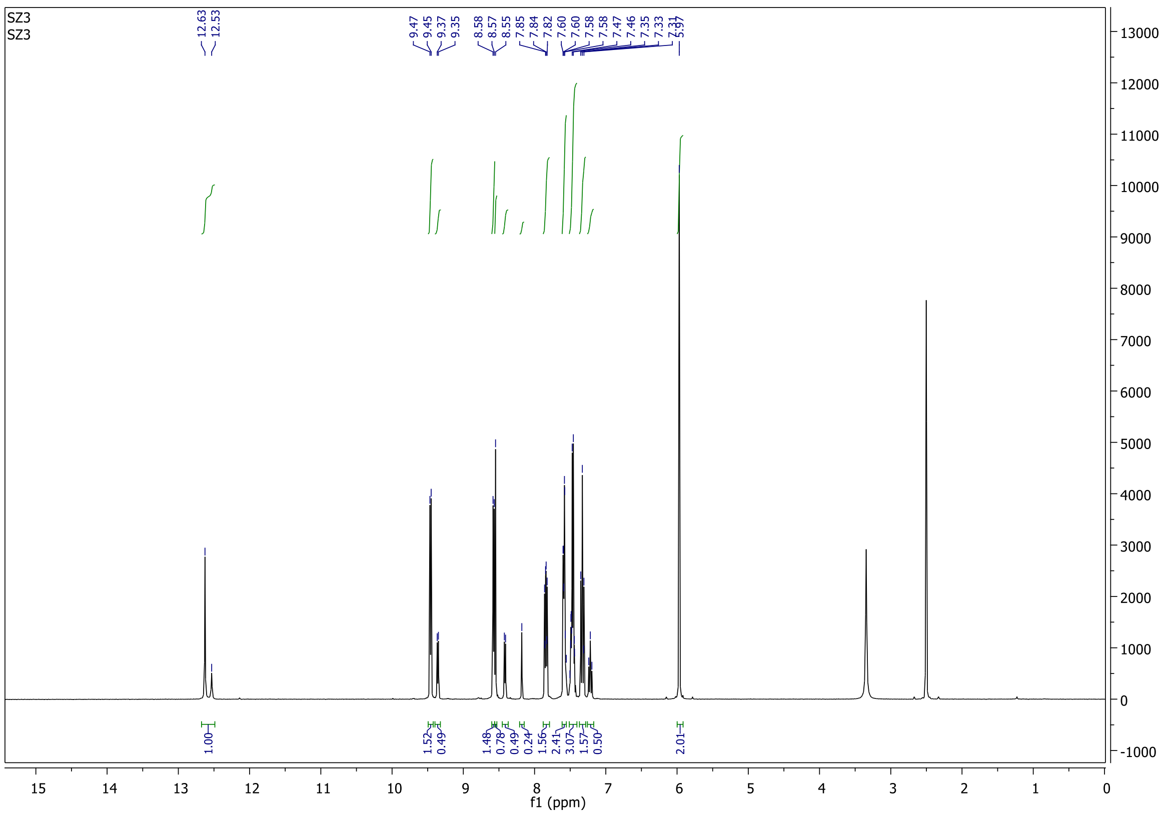

Figure S91. ^{1}HNMR of Compound 22

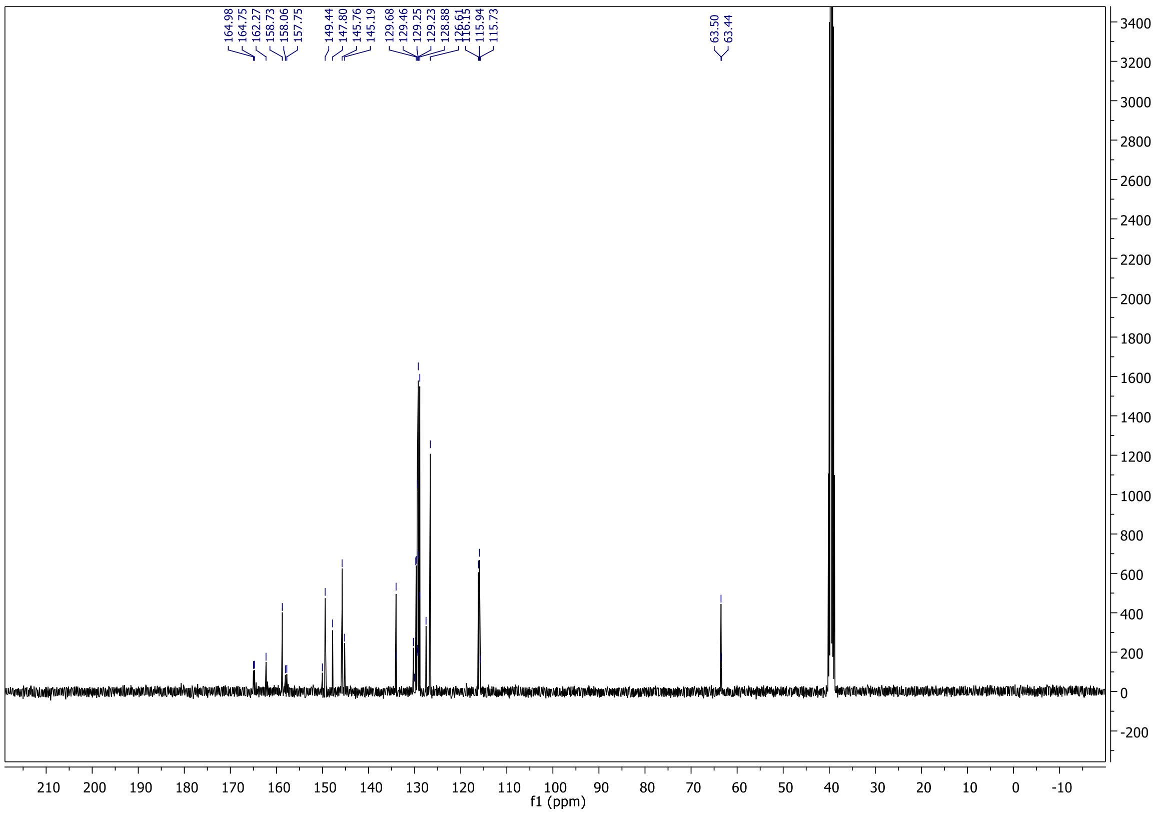

Figure S92. ^{13}CNMR of Compound 22

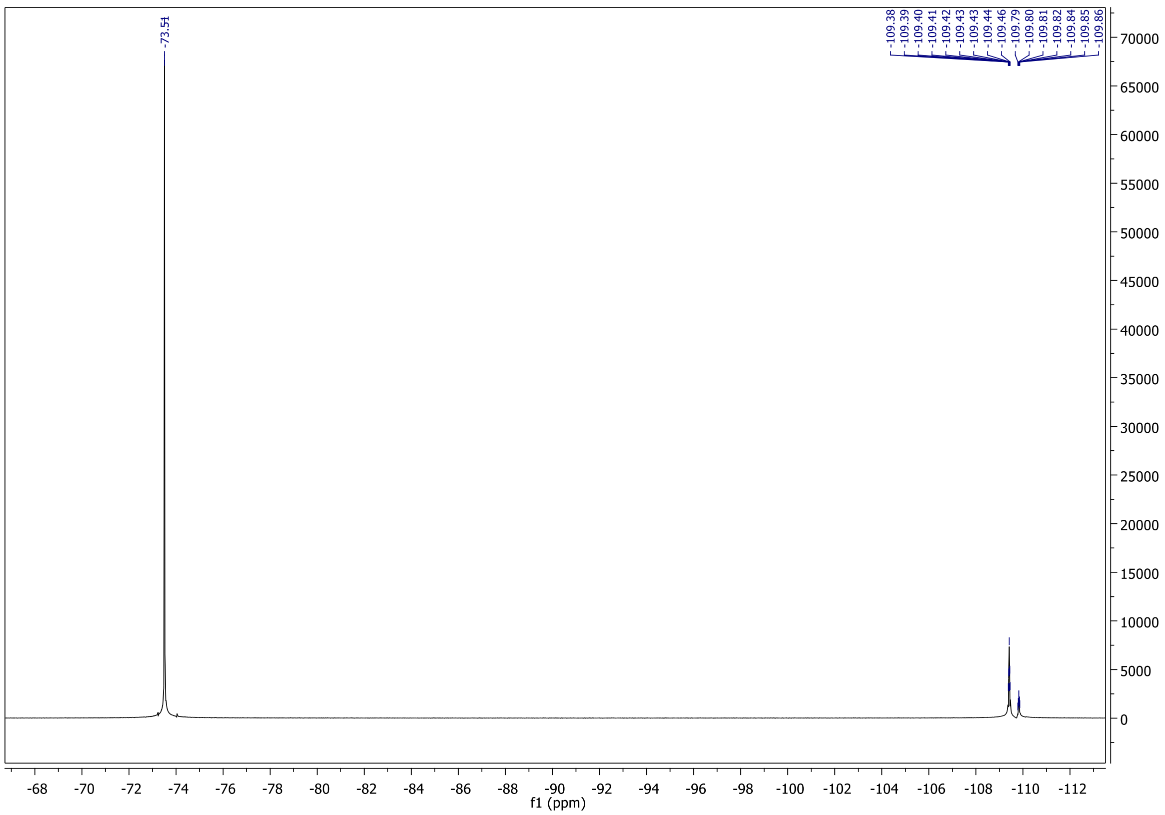

Figure S93. ^{19}FNMR of Compound 22

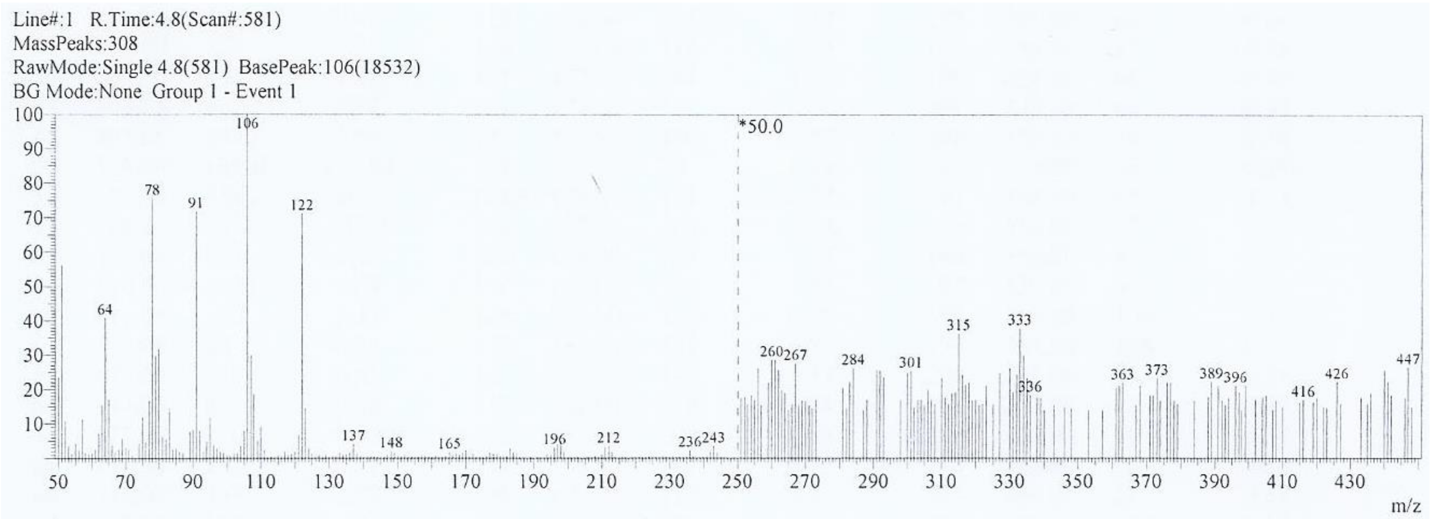

Figure S94. MS (ESI) of Compound 22

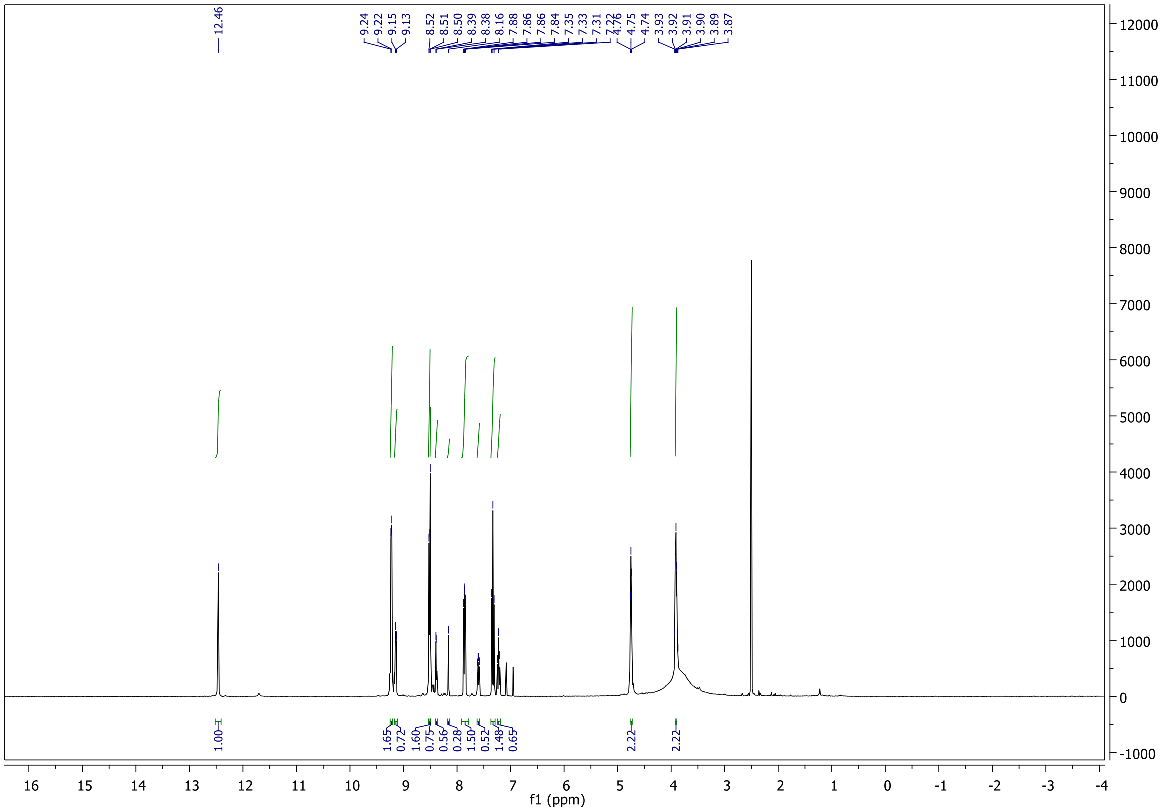

Figure S95. ^{1}HNMR of Compound 23

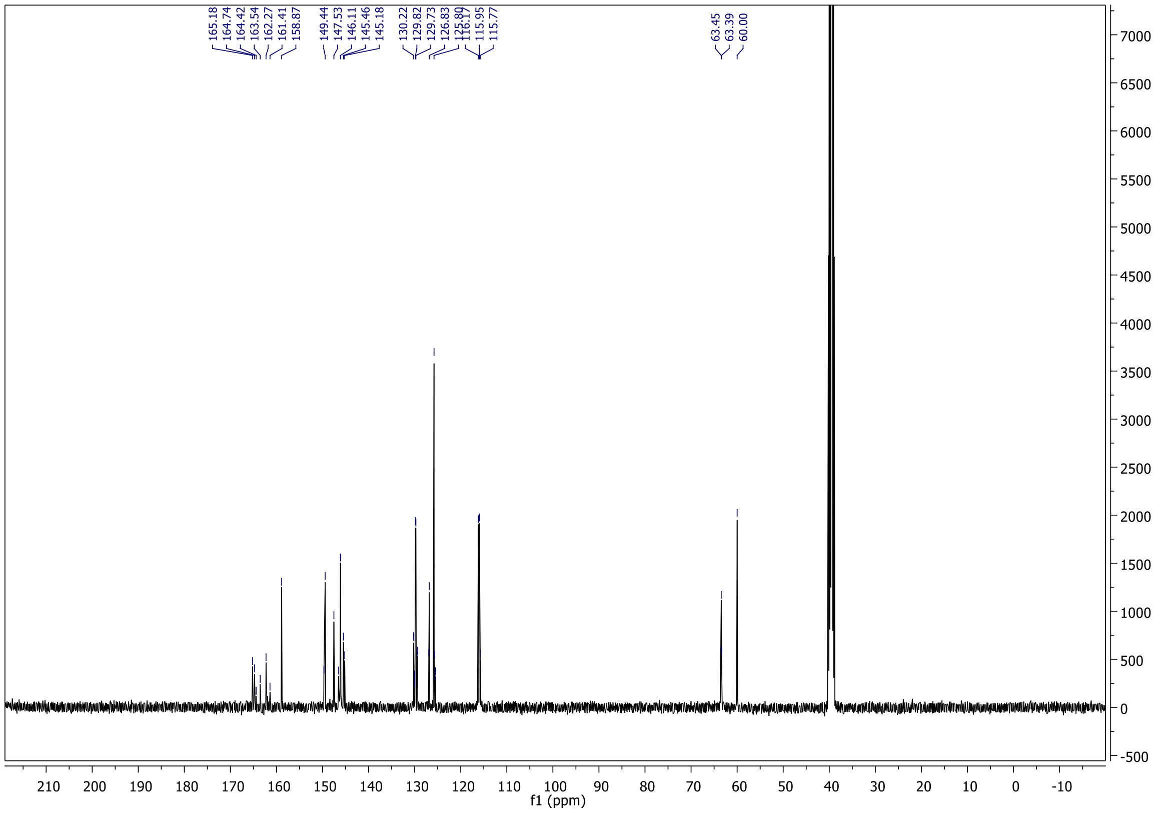

Figure S96. ^{13}CNMR of Compound 23

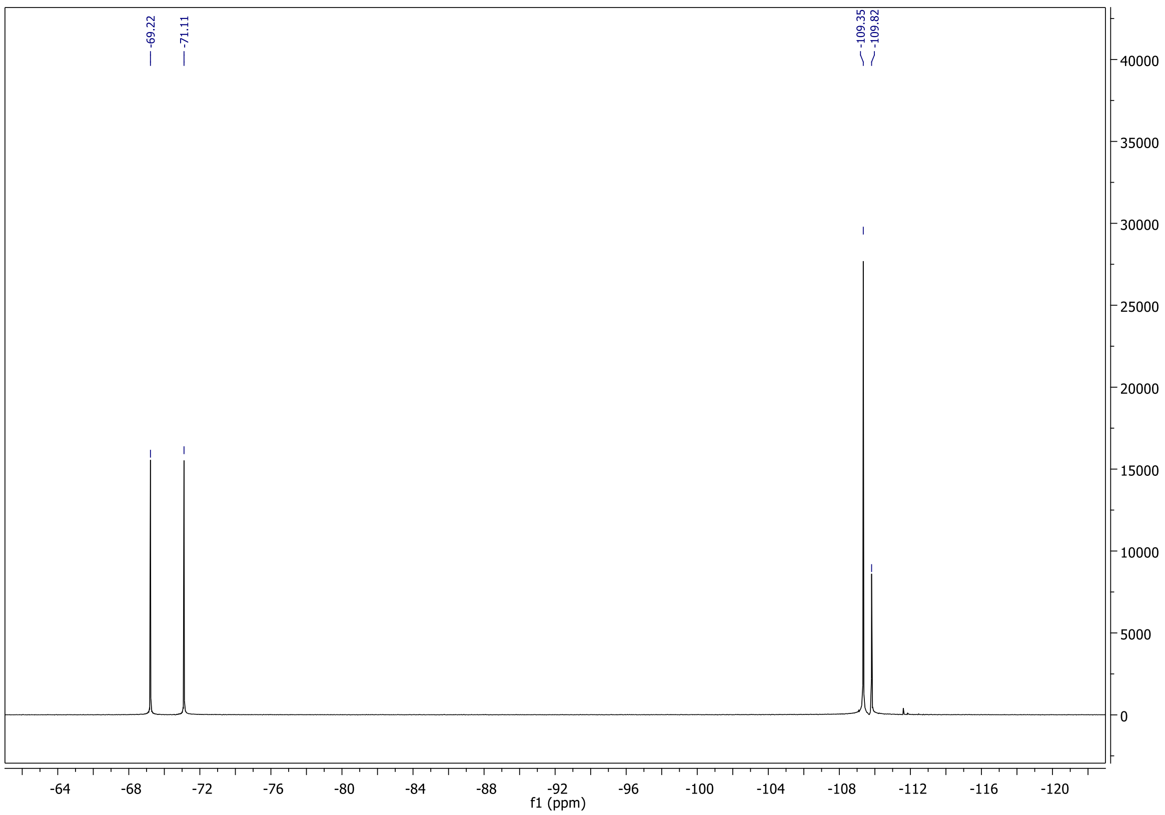

Figure S97. ^{19}FNMR of Compound 23

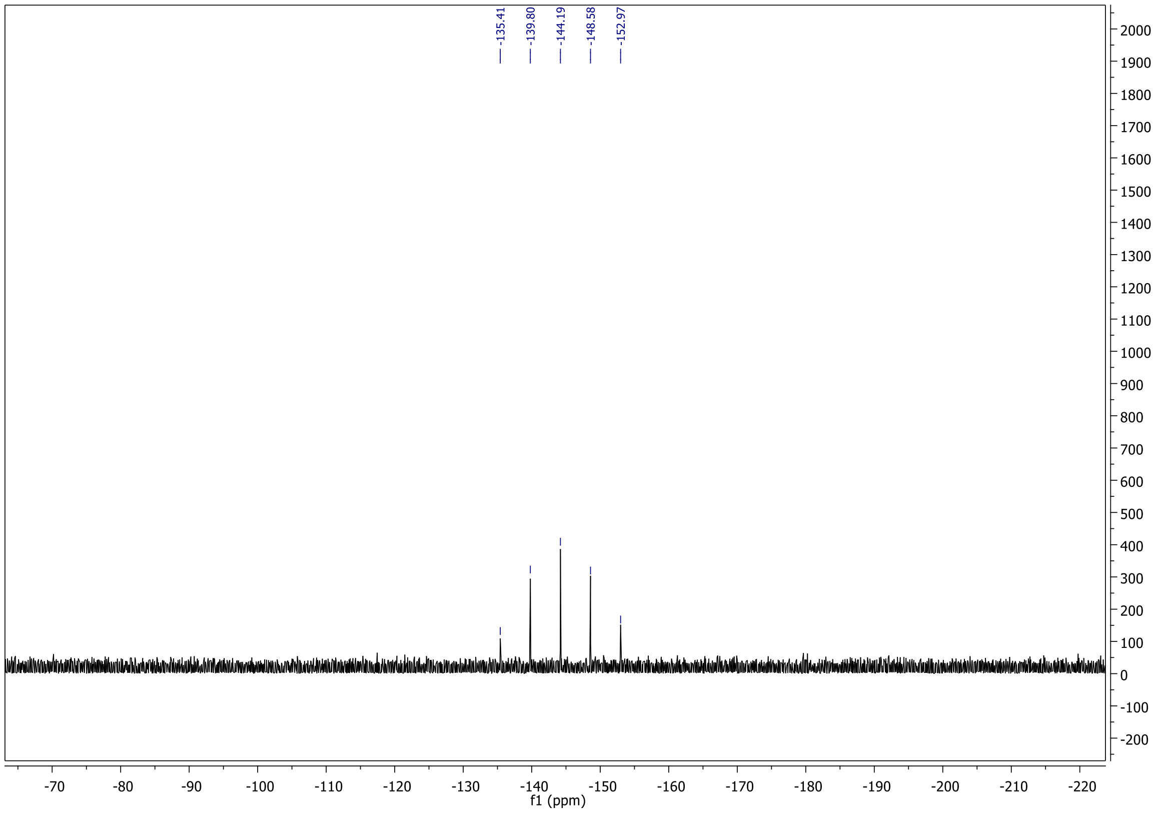

Figure S98. ^{31}PNMR of Compound 23

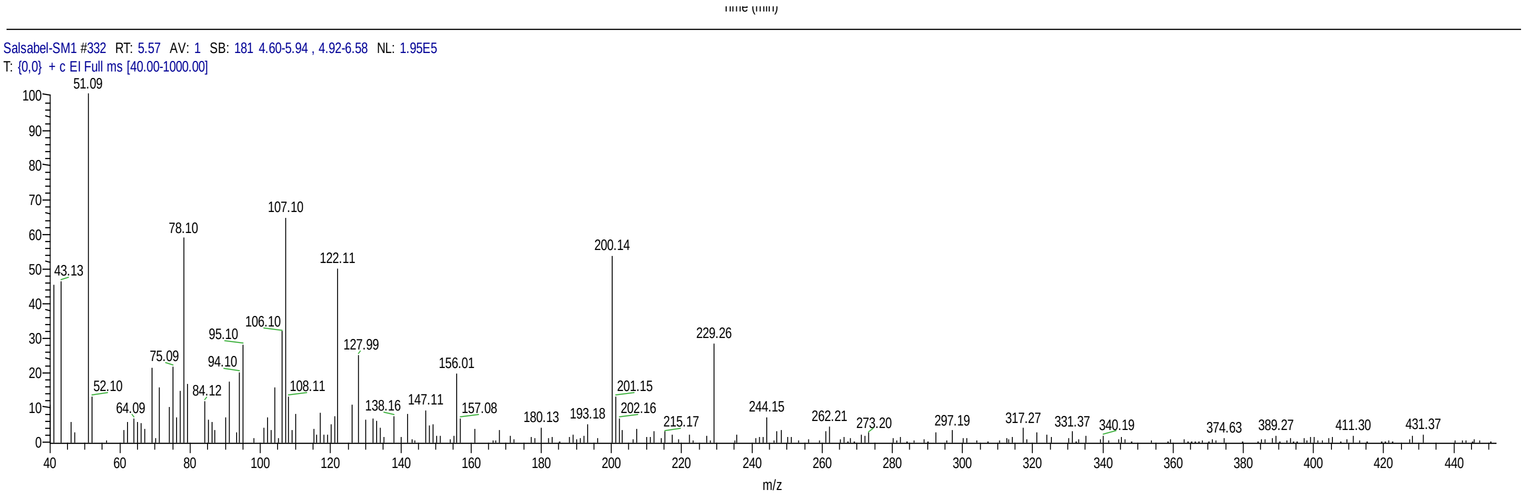

Figure S99. MS (ESI) of Compound 23

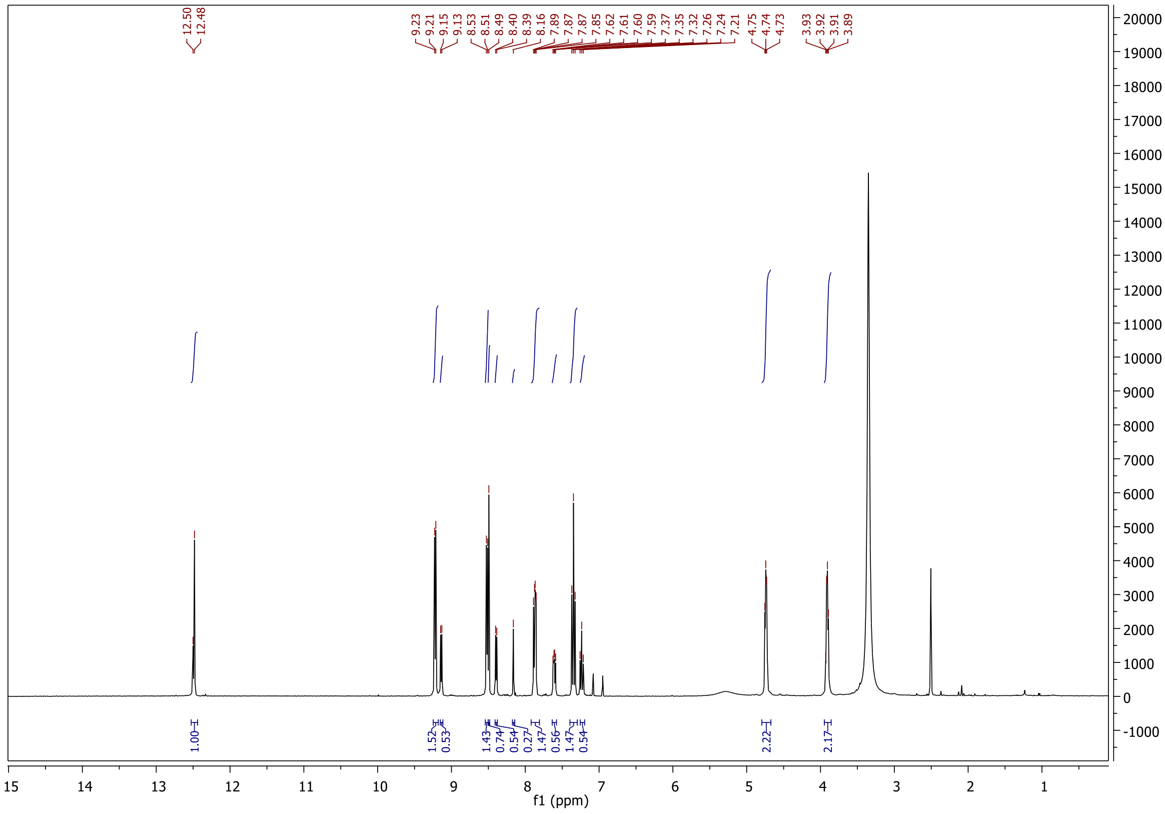

Figure S100. ^{1}HNMR of Compound 24

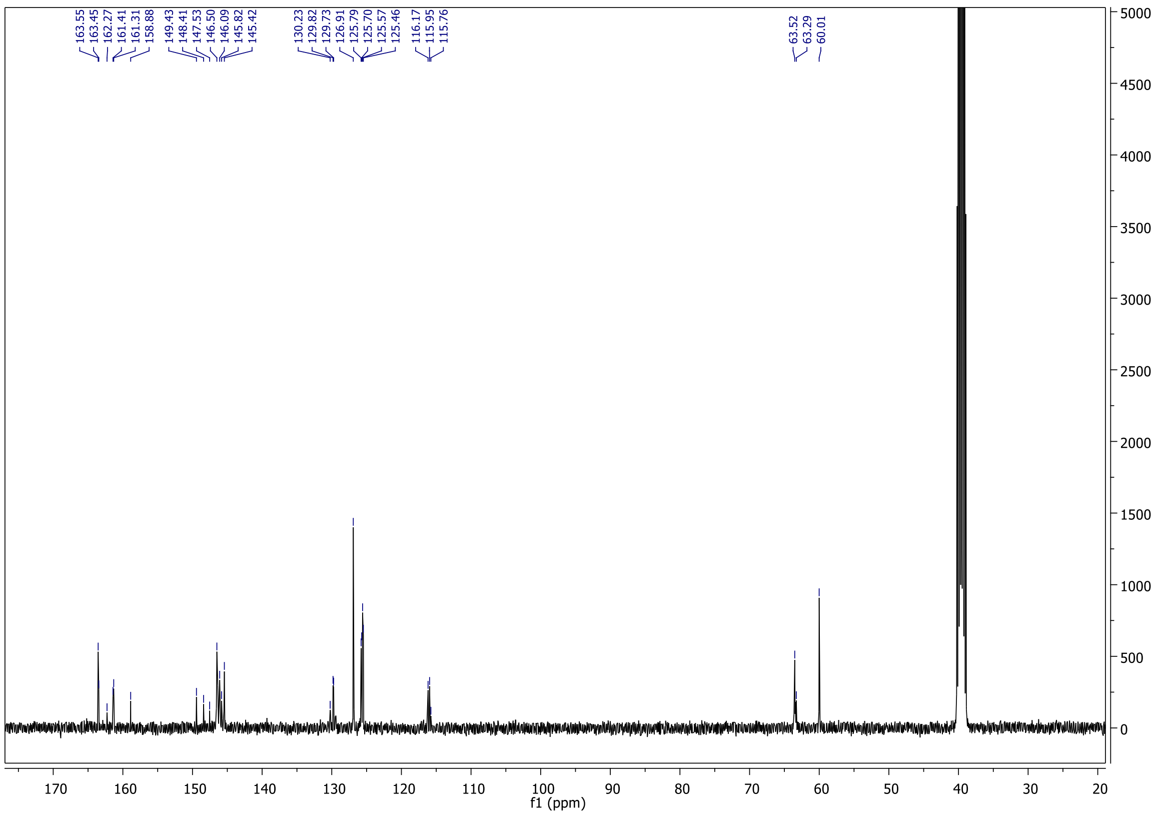

Figure S101. ^{13}CNMR of Compound 24

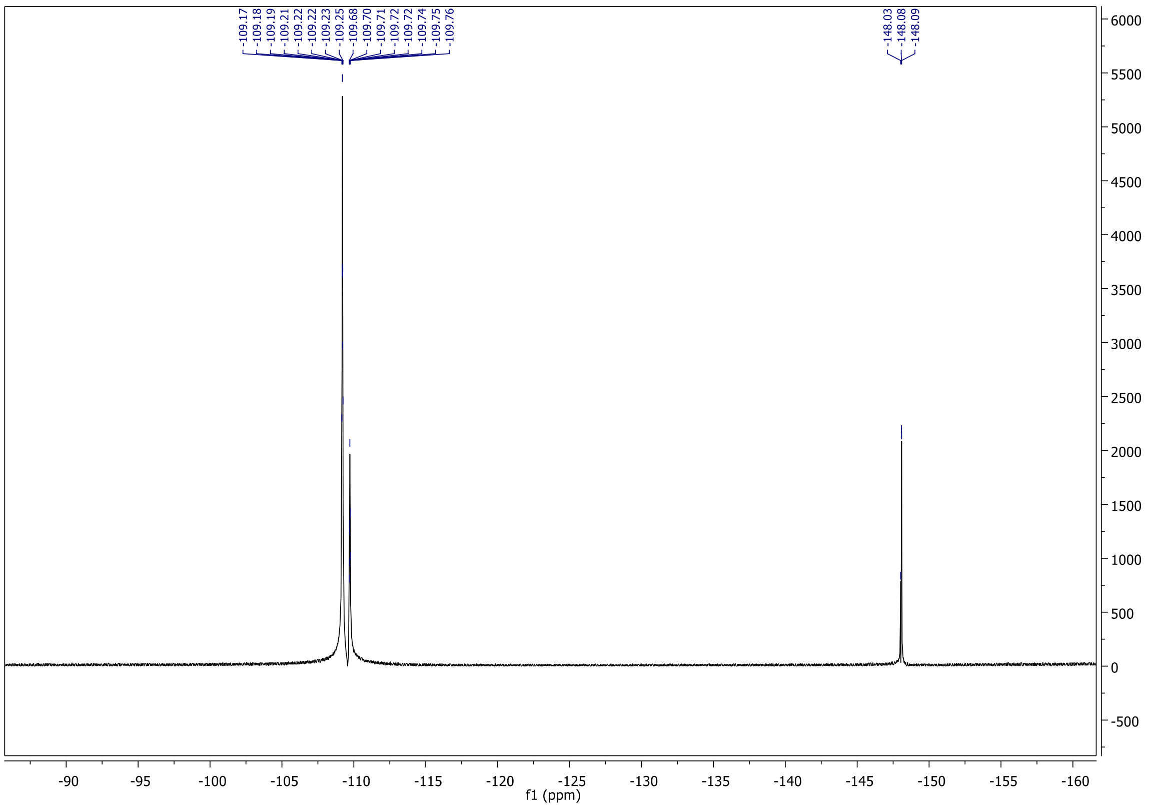

Figure S102. ^{19}FNMR of Compound 24

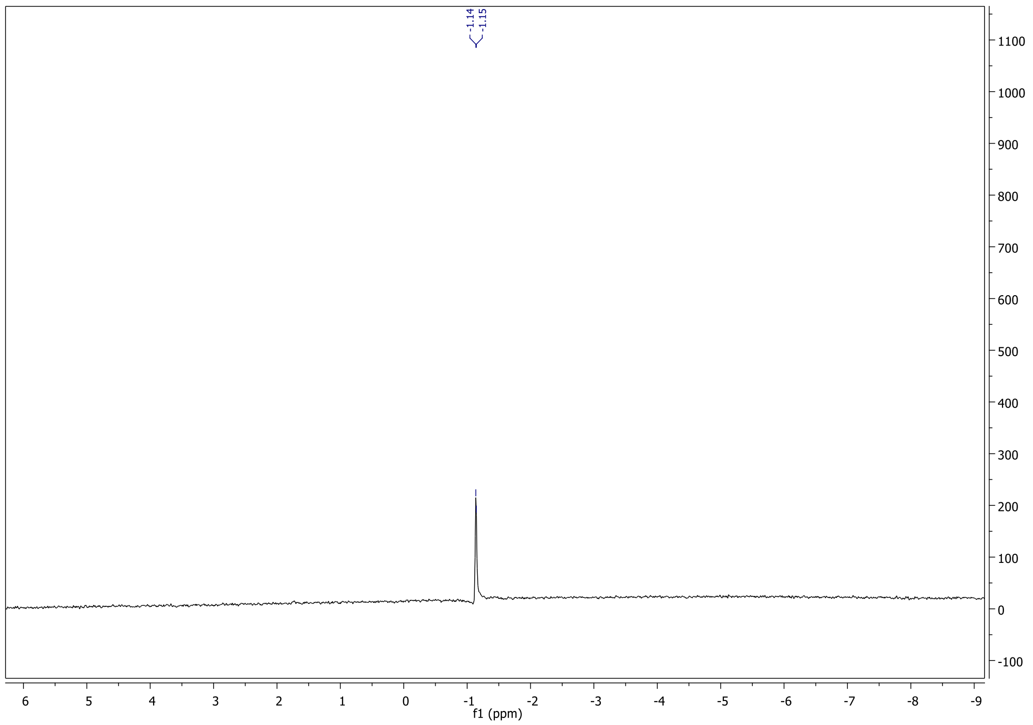

Figure S103. ^{11}BNMR of Compound 24

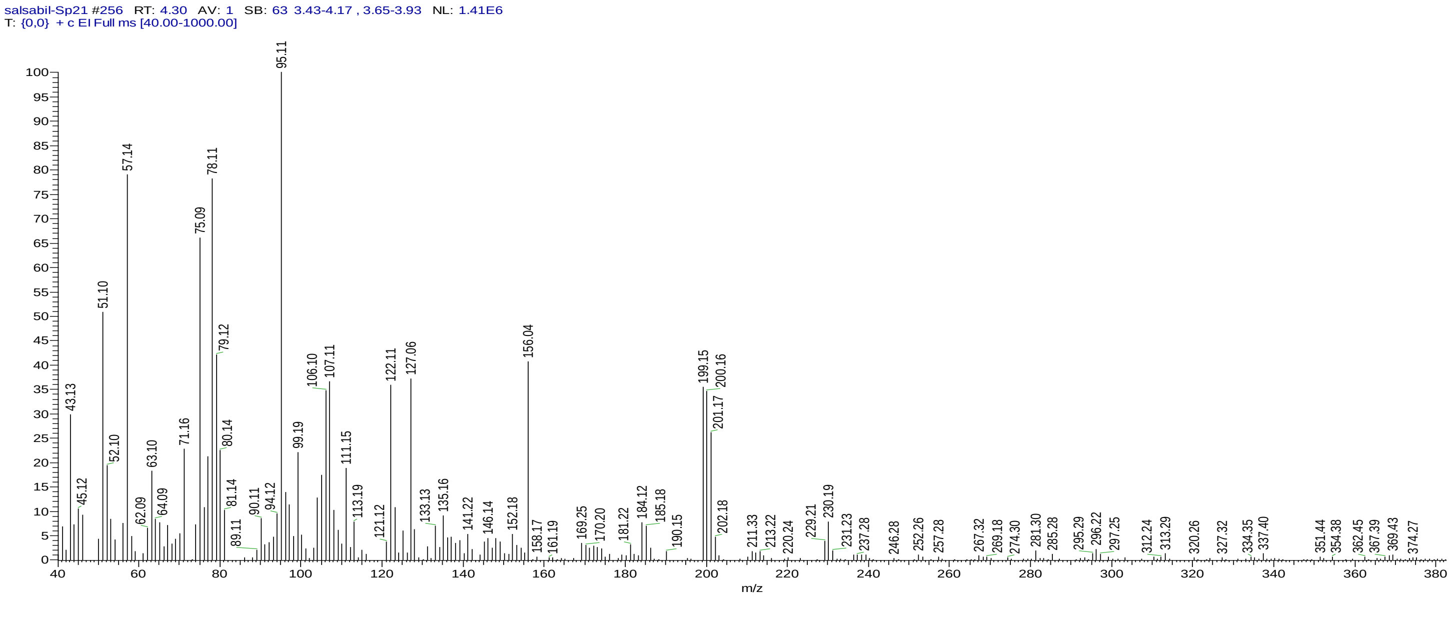

Figure S104. MS (ESI) of Compound 24

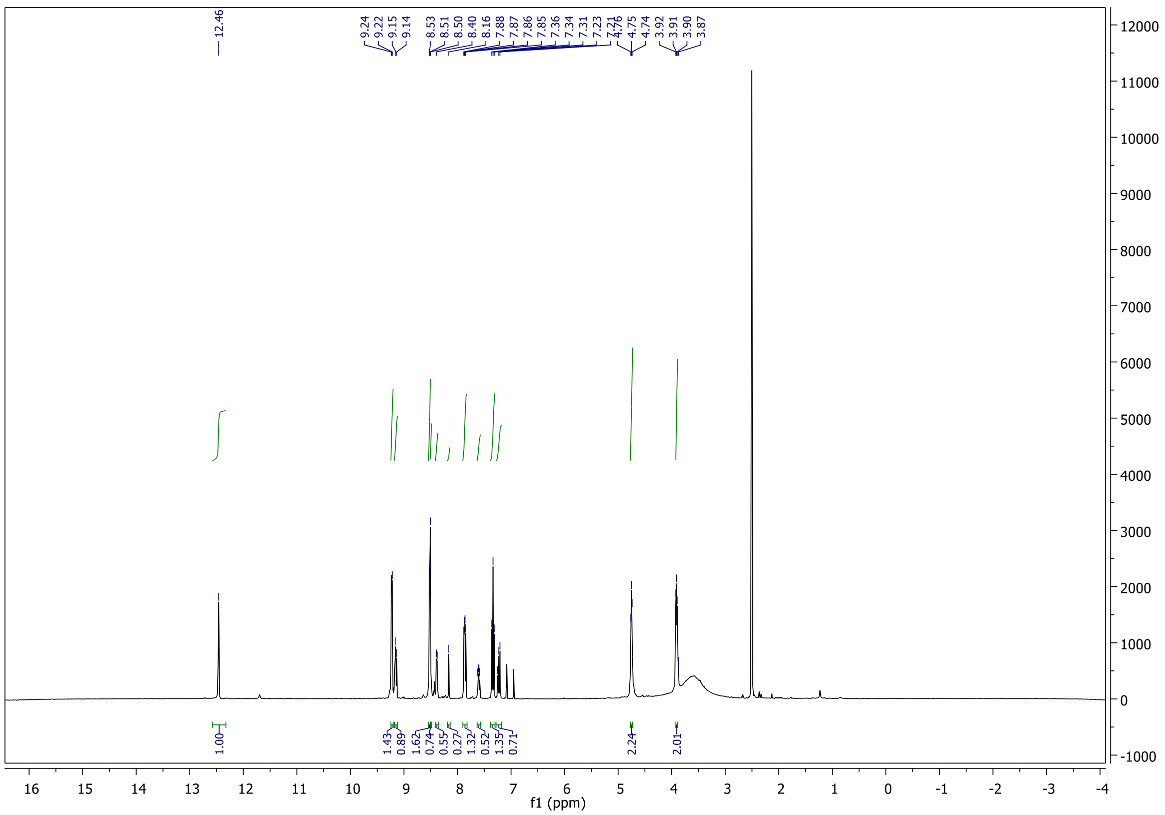

Figure S105. ^{1}HNMR of Compound 25

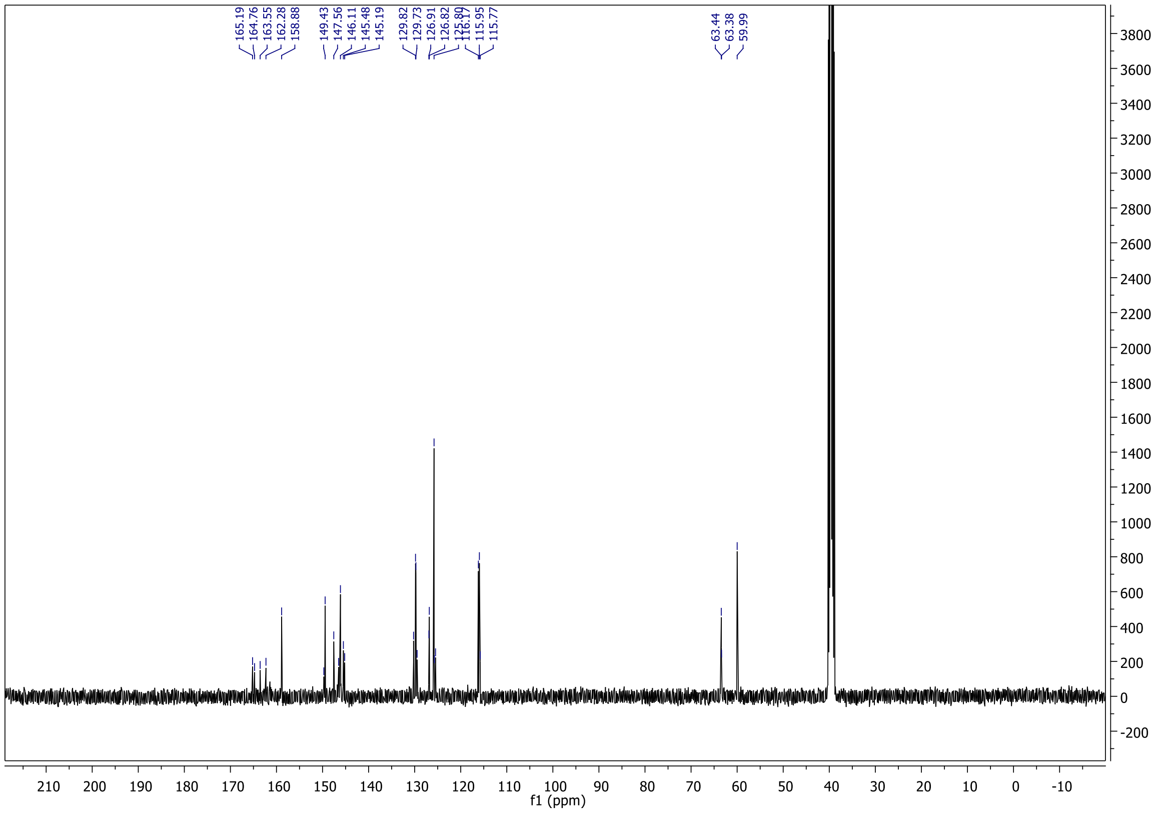

Figure S106. ^{13}CNMR of Compound 25

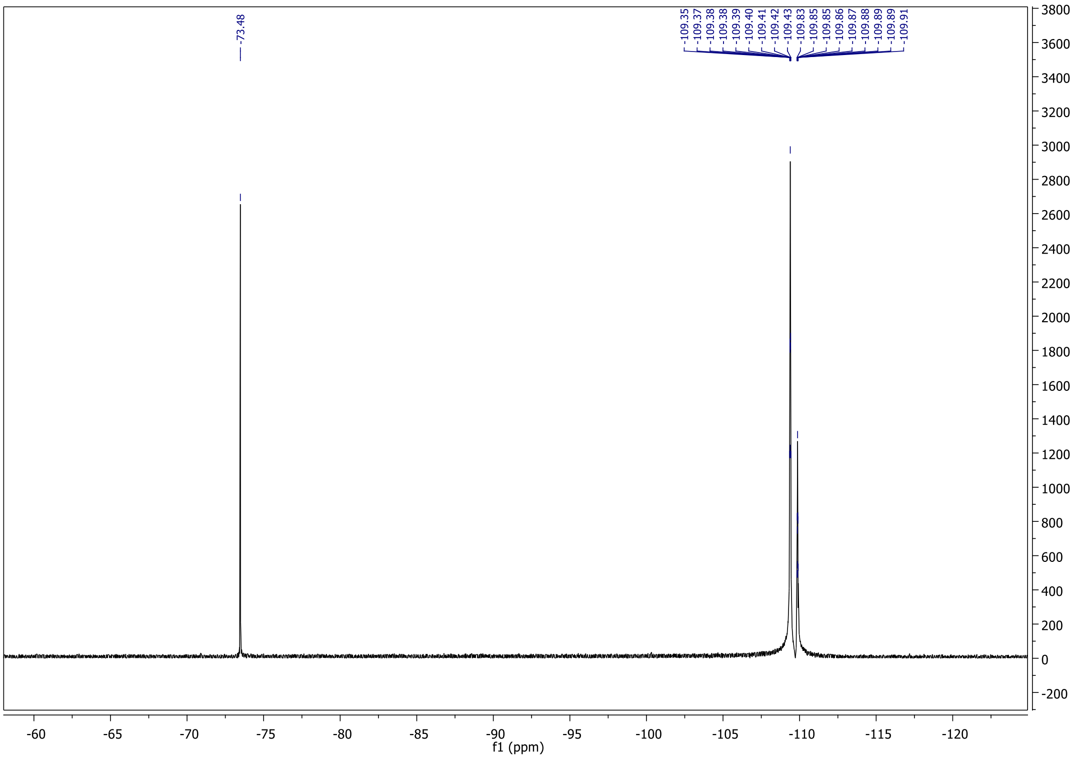

Figure S107. ^{19}FNMR of Compound 25

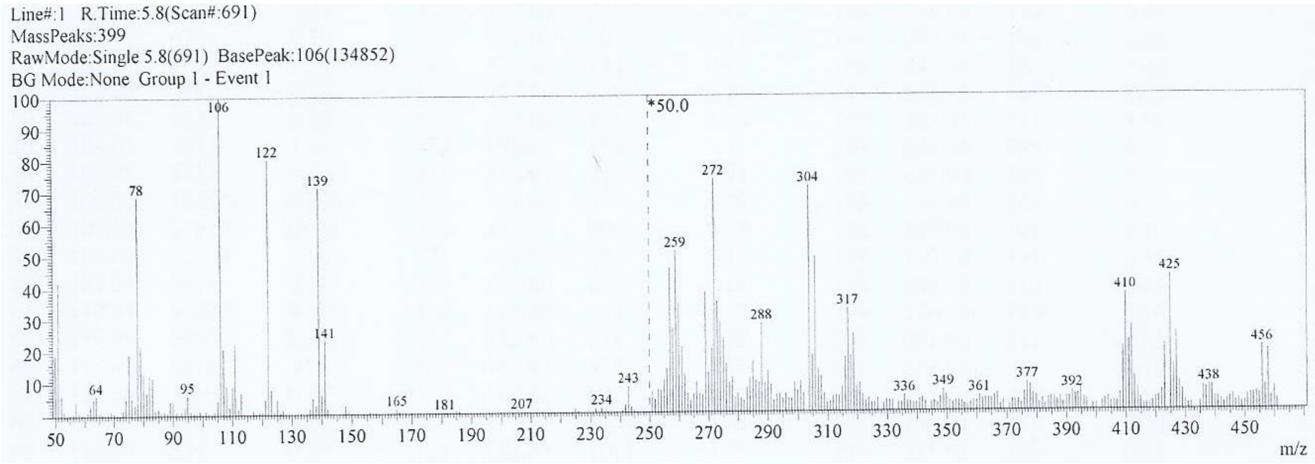

Figure S108. MS (ESI) of Compound 25
